# Supplementary material for: Evaluation of an automated feedback intervention to improve antimicrobial prescribing among primary care physicians (OPEN Stewardship): protocol for an interrupted time-series and usability analysis in Ontario, Canada and Southern Israel
Source: BMJ Open. 2021 Jan 13;11(1):e039810. doi: 10.1136/bmjopen-2020-039810 (PMC7812099; doi:10.1136/bmjopen-2020-039810)
Supplement: Supplementary data [file bmjopen-2020-039810supp001.pdf]

Table S1. ICD-9 codes for indications identified as acute sinusitis and viral respiratory conditions in the OPEN Stewardship platform.

**OPEN Stewardship category**	**Indication**	**ICD-9 codes**
Acute sinusitis	Acute sinusitis	461 Acute sinusitis
Viral respiratory conditions	Asthma/allergy	477 Allergic rhinitis, 493 Asthma, 995.3 Allergy, unspecified
	Bronchitis/bronchiolitis	466 Acute bronchitis and bronchiolitis, 490 Bronchitis, not specified as acute or chronic, 492 Emphysema, 496 Chronic airway obstruction, not elsewhere classified
	Influenza	487 Influenza, 488 Influenza due to identified avian influenza virus
	Viral pneumonia	480 Viral pneumonia
	Viral upper respiratory tract infection	460 Acute nasopharyngitis [common cold], 464 Acute laryngitis and tracheitis, 465 Acute upper respiratory infections of multiple or unspecified sites, 786.2 Cough

Appendix 1. Survey questions for participants in the OPEN Stewardship trial (English).

Introductory text: Thank you for agreeing to receive the OPEN Stewardship report on antibiotic prescribing. Help us improve future reports by filling out this short survey. It will only take 3-5 minutes. Questions are multiple choice with the option to provide comments at the end of the survey. Your feedback is important to us and your opinion matters!

Survey 1 (t_0)

Q1. Is the purpose of the OPEN Stewardship report clear?

- Yes
- No
- Don’t know

Q2. How informative did you find the following item in the OPEN Stewardship report: Your Prescribing Rate per Visit Compared to Peers (example below - not your data)?

Number of Prescriptions of Any Antibiotic (Per 100 Visits) for Any Indication

✓ You prescribed 11% below average

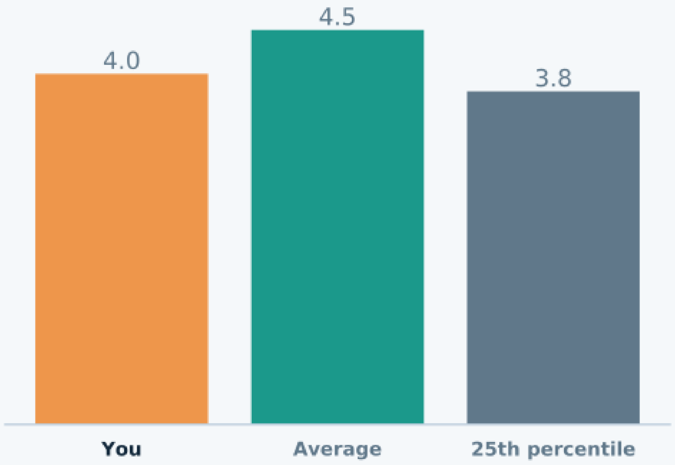

You had 5001 patient visits from Jan 01, 2019 to Dec 31, 2019 .

This figure shows how frequently you prescribed antibiotics for any indication in the previous year (2019). Your prescribing rate (prescriptions per 100 visits) is compared to the average (mean) of your colleagues and participants in Southern Ontario as well as showing your position relative to the 25th percentile. Generally, it is better to be below the average rate of prescribing and within the lowest 25th percentile.

These data are not adjusted for physician's practice characteristics, nor do they differentiate between delayed or non-filled prescriptions. These results are generated from claims data, and misclassification of the diagnosis may be present. As a result, our prescribing targets are referenced to peer benchmarks. Learn more about the data and its interpretation.

- Very informative
- Somewhat informative
- Not so informative
- Not at all informative
- Don’t know

Q3. Was it straightforward how to navigate through the OPEN Stewardship report?

- Yes
- No
- Don't know

Q4. Is it useful to include *Best Practice Guidelines* in the OPEN Stewardship report (sample from most recent report below)?

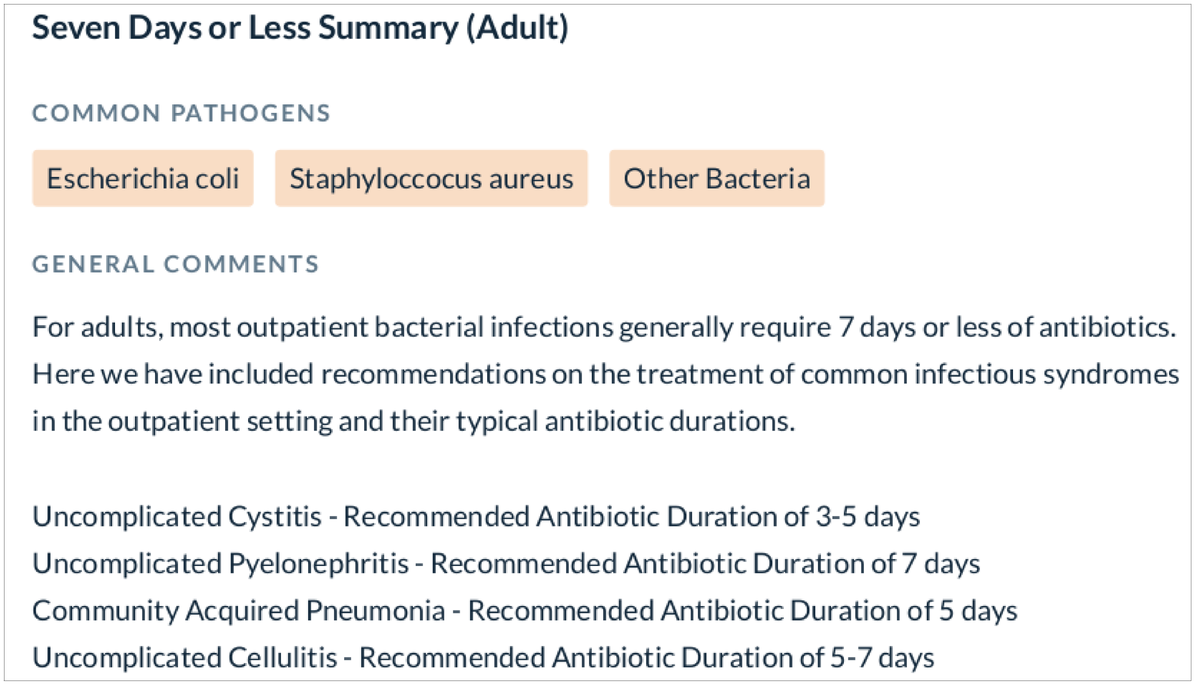

Seven Days or Less Summary (Adult)

COMMON PATHOGENS

Escherichia coli | Staphyloccocus aureus | Other Bacteria

GENERAL COMMENTS

For adults, most outpatient bacterial infections generally require 7 days or less of antibiotics. Here we have included recommendations on the treatment of common infectious syndromes in the outpatient setting and their typical antibiotic durations.

Uncomplicated Cystitis - Recommended Antibiotic Duration of 3-5 days
Uncomplicated Pyelonephritis - Recommended Antibiotic Duration of 7 days
Community Acquired Pneumonia - Recommended Antibiotic Duration of 5 days
Uncomplicated Cellulitis - Recommended Antibiotic Duration of 5-7 days

- Yes
- No
- Don't know

Q5. Rate each of the following items according to its potential usefulness to you in an OPEN Stewardship report. Note that not all of these items were included in the report sent to you. (Very useful, Somewhat useful, Not so useful, Not at all useful, Don't know)

- See how my prescribing changes over time
- Compare my own prescribing with peers
- Get an overview of local prescribing patterns
- Get an overview of local resistance levels
- Compare my own prescribing with guidelines
- Have discussions with peers on the local situation regarding prescribing patterns

Q6. Overall, how would you rate the usefulness of the OPEN Stewardship report?

- Very useful
- Somewhat useful
- Not so useful

- Not at all useful
- Don’t know

Q7. Please suggest how to improve the OPEN Stewardship report (such as layout of the graphs and information included).

[Free text box]

Q8. Any additional comments?

[Free text box]

Survey 2 (t_3)

Q1. How informative did you find the following item in the OPEN Stewardship report: Your Prescribing Rate per Visit Compared to Peers (example below - not your data)?

Number of Prescriptions of Any Antibiotic (Per 100 Visits) for Any Indication

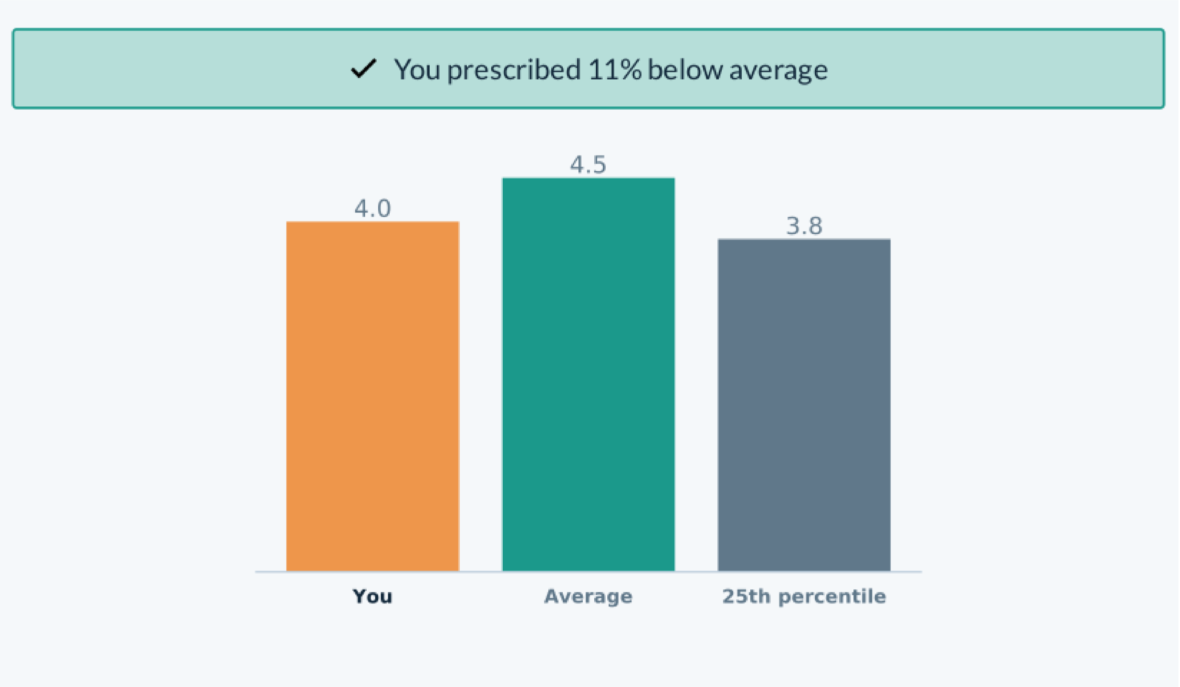

You had 5001 patient visits from Jan 01, 2019 to Dec 31, 2019 .

This figure shows how frequently you prescribed antibiotics for any indication in the previous year (2019). Your prescribing rate (prescriptions per 100 visits) is compared to the average (mean) of your colleagues and participants in Southern Ontario as well as showing your position relative to the 25th percentile. Generally, it is better to be below the average rate of prescribing and within the lowest 25th percentile.

These data are not adjusted for physician's practice characteristics, nor do they differentiate between delayed or non-filled prescriptions. These results are generated from claims data, and misclassification of the diagnosis may be present. As a result, our prescribing targets are referenced to peer benchmarks. Learn more about the data and its interpretation.

- Very informative
- Somewhat informative
- Not so informative
- Not at all informative
- Don’t know

Q2. In the past three months, did you ever consider the previous OPEN Stewardship report when making an antibiotic prescribing decision?

- Yes
- No
- Don't know

Q3. Rate each of the following items according to its potential usefulness to you in an OPEN Stewardship report. Note that not all of these items were included in the report sent to you. (Very useful, Somewhat useful, Not so useful, Not at all useful, Don't know)

- See how my prescribing changes over time
- Compare my own prescribing with peers
- Get an overview of local prescribing patterns
- Get an overview of local resistance levels
- Compare my own prescribing with guidelines
- Have discussions with peers on the local situation regarding prescribing patterns

Q4. Overall, how would you rate the usefulness of the OPEN Stewardship report?

- Very useful
- Somewhat useful
- Not so useful
- Not at all useful
- Don't know

Q5. Please suggest how to improve the OPEN Stewardship report (such as layout of the graphs and information included).

[Free text box]

Q6. Any additional comments?

[Free text box]

Survey 3 (t_6)

Q1. How informative did you find the following item in the OPEN Stewardship report: Your Prescribing Rate per Visit Compared to Peers (example below - not your data)?

Number of Prescriptions of Any Antibiotic (Per 100 Visits) for Any Indication

✓ You prescribed 11% below average

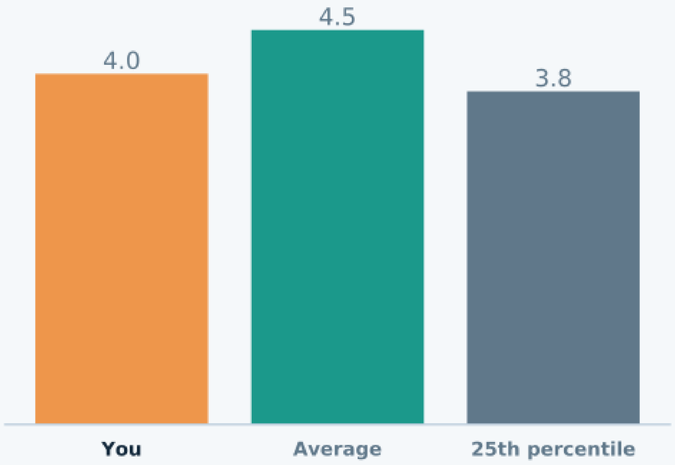

You had 5001 patient visits from Jan 01, 2019 to Dec 31, 2019 .

This figure shows how frequently you prescribed antibiotics for any indication in the previous year (2019). Your prescribing rate (prescriptions per 100 visits) is compared to the average (mean) of your colleagues and participants in Southern Ontario as well as showing your position relative to the 25th percentile. Generally, it is better to be below the average rate of prescribing and within the lowest 25th percentile.

These data are not adjusted for physician's practice characteristics, nor do they differentiate between delayed or non-filled prescriptions. These results are generated from claims data, and misclassification of the diagnosis may be present. As a result, our prescribing targets are referenced to peer benchmarks. Learn more about the data and its interpretation.

- Very informative
- Somewhat informative
- Not so informative
- Not at all informative
- Don't know

Q2. In the past three months, did you ever consider the previous OPEN Stewardship report when making an antibiotic prescribing decision?

- Yes
- No
- Don’t know

Q3. Overall, how would you rate the usefulness of the OPEN Stewardship report?

- Very useful
- Somewhat useful
- Not so useful
- Not at all useful
- Don’t know

Q4. Hypothetically, how often do you think it would be appropriate to receive OPEN Stewardship reports?

- Monthly
- Quarterly
- Every six months
- Annually
- Not at all
- Don’t know

Q5. Would a central repository of local treatment guidelines be useful for your practice?

- Yes
- No
- Don’t know

Q6. Do you think it would be useful to have access to a personalized web-portal where you could browse your own and local patterns of antibiotic use, as well as best practice guidelines?

- Yes
- No
- Don’t know

Q7. Would you be willing to participate in a follow-up interview where we would ask in more detail about your experiences with the OPEN Stewardship reports? If so, please send an email to [study email address].

Q8. Please suggest how to improve the OPEN Stewardship report (such as layout of the graphs and information included).

[Free text box]

Q9. Any additional comments?

[Free text box]

Appendix 2. Survey questions for participants in the OPEN Stewardship trial (Hebrew).

Introductory text:

רופא יקר,

אנו מודים לך שוב על הסכמתך לקחת חלק במחקר המשותף של אגף רפואה בחטיבת הקהילה בשירותי בריאות כללית והמחלקה לניהול מערכות בריאות באוניברסיטת בן גוריון בנגב בנושא "דפוסי רישום תרופות אנטיביוטיות על-ידי רופאי משפחה".

נודה לך אם תוכל להקדיש שלוש עד חמש דקות מזמנך כדי לענות על השאלון שלהלן, המתייחס לדו"ח האישי שנשלח אליך לאחרונה בנושא מרשמי האנטיביוטיקה של מטופליך. תשובותיך יעזרו לנו לשפר את אופן העברת המידע לרופאים בהמשך.

השאלון נכתב בלשון זכר מטעמי נוחות, אך מיועד לשני המינים.

Survey 1 (t_0)

שאלה 1: האם מטרת הדו"ח האישי ששלחנו לך, ברורה, לדעתך?

- כן
- לא
- לא יודע

שאלה 2: באיזו מידה הפריט הבא - "שיעור מרשמי האנטיביוטיקה שרשמת פר ביקור מטופל בהשוואה לעמיתיך" - סיפק לך את המידע הדרוש לך (הנתונים למטה לצורך דוגמא בלבד - אינם הנתונים שלך)?

Number of Prescriptions of Any Antibiotic (Per 100 Visits) for Any Indication

✓ You prescribed 11% below average

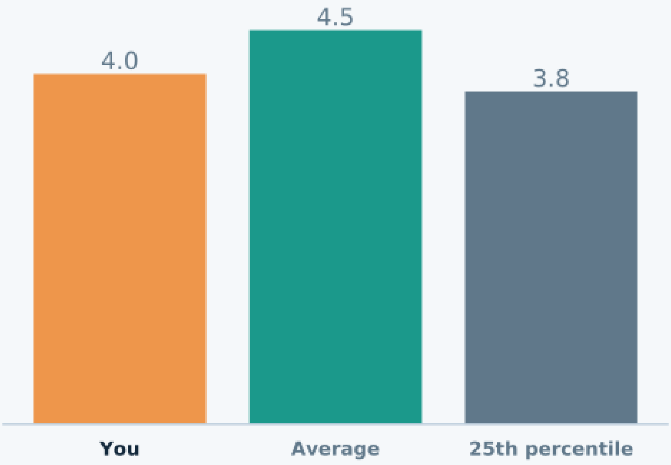

You had 5001 patient visits from Jan 01, 2019 to Dec 31, 2019 .

This figure shows how frequently you prescribed antibiotics for any indication in the previous year (2019). Your prescribing rate (prescriptions per 100 visits) is compared to the average (mean) of your colleagues and participants in Southern Ontario as well as showing your position relative to the 25th percentile. Generally, it is better to be below the average rate of prescribing and within the lowest 25th percentile.

These data are not adjusted for physician's practice characteristics, nor do they differentiate between delayed or non-filled prescriptions. These results are generated from claims data, and misclassification of the diagnosis may be present. As a result, our prescribing targets are referenced to peer benchmarks. Learn more about the data and its interpretation.

- מידה רבה
- במידה מסוימת
- במידה מועטה
- בכלל לא
- לא יודע

שאלה 3: האם הדוח האישי היה קל להבנה?

- כן

- לא
- לא יודע

שאלה 4: האם יש, לדעתך, תועלת בצירוף קווים מנחים לדו"ח האישי (ראה דוגמה להלן)?

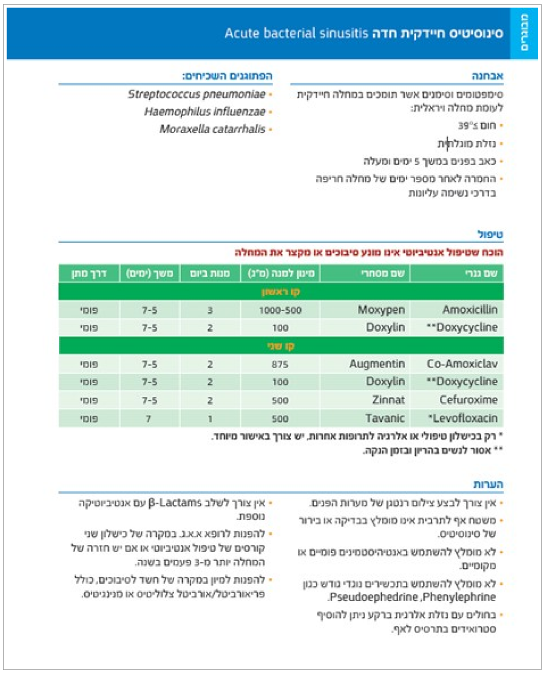
מבוגרים

סינוסיטיס חיידקית חדה Acute bacterial sinusitis

אבחנה

סימפטומים וסימנים אשר תומכים במחלה חיידקית לעומת מחלה ויראלית:

- חום ≥39°צ
- נזלת מוגלתית
- כאב בפנים במשך 5 ימים ומעלה
- החמרה לאחר מספר ימים של מחלה חריפה בדרכי נשימה עליונות

הפתוגנים השכיחים:

- *Streptococcus pneumoniae*
- *Haemophilus influenzae*
- *Moraxella catarrhalis*

טיפול

הוכח שטיפול אנטיביוטי אינו מונע סיבוכים או מקצר את המחלה

שם גנרי	שם מסחרי	מינון למנה (מ"ג)	מנות ביום	משך (ימים)	דרך מתן
קו ראשון					
Amoxicillin	Moxypen	1000-500	3	7-5	פומי
**Doxycycline	Doxylin	100	2	7-5	פומי
קו שני					
Co-Amoxiclav	Augmentin	875	2	7-5	פומי
**Doxycycline	Doxylin	100	2	7-5	פומי
Cefuroxime	Zinnat	500	2	7-5	פומי
*Levofloxacin	Tavanic	500	1	7	פומי

*** רק בכישלון טיפולי או אלרגיה לתרופות אחרות, יש צורך באישור מיוחד.**
**** אסור לנשים בהריון ובזמן הנקה.**

הערות

- אין צורך לבצע צילום רנטגן של מערות הפנים.
- משטח אף לתרבית אינו מומלץ בבדיקה או בירור של סינוסיטיס.
- לא מומלץ להשתמש באנטיהיסטמינים פומיים או מקומיים.
- לא מומלץ להשתמש בתכשירים נוגדי גודש כגון Phenylephrine, Pseudoephedrine.
- בחולים עם נזלת אלרגית ברקע ניתן להוסיף סטרואידים בתרסיס לאף.
- אין צורך לשלב β-Lactams עם אנטיביוטיקה נוספת.
- להפנות לרופא א.א.ג. במקרה של כישלון שני קורסים של טיפול אנטיביוטי או אם יש חזרה של המחלה יותר מ-3 פעמים בשנה.
- להפנות למיון במקרה של חשד לסיבוכים, כולל פריאורביטל/אורביטל צלוליטיס או מנינגיטיס.

- כן
- לא
- לא יודע

שאלה 5: אנא הענק ציון לכל אחד מההיגדים הבאים, בהתאם לתועלת הפוטנציאלית עבורך בדו"ח האישי ששלחנו לך שים לב, לא כל המשפטים/היגדים שאתה נשאל עליהם הופיעו בדו"ח האישי שקבלת. (מועיל מאוד, מועיל במידה מסוימת, מועיל במידה מועטה, כלל לא מועיל, לא יודע)

- אני יכול לראות כיצד דפוס רישום האנטיביוטיקה שלי משתנה לאורך זמן
- אני יכול להשוות את דפוס רישום האנטיביוטיקה שלי לאלה של עמיתיי
- אני יכול לקבל סקירה של דפוסי המרשם המקומיים
- קבלת דוח סקירה מקומי על רמות עמידות
- אני יכול להשוות את דפוס הרישום שלי לעומת הקווים המנחים של הכללית
- מאפשר לי לדון עם עמיתיי על מצב דפוסי המרשם המקומיים

שאלה 6: בסך הכל, איזה ציון היית מעניק לתועלת שאתה מפיק מהדו"ח האישי?

- מועיל מאוד
- מועיל במידה מסוימת
- מועיל במידה מועטה
- כלל לא מועיל
- לא יודע

שאלה 7: אנא הצע דרכים לשיפור הדו"ח האישי (כמו עיצוב הגרף והפרמטרים).

[טקסט חופשי]

שאלה 8: הערות נוספות?

[טקסט חופשי]

Survey 2 (t_3)

שאלה 1: באיזו מידה הפריט הבא - "שיעור מרשמי האנטיביוטיקה שרשמת פר ביקור מטופל בהשוואה לעמיתיך" - סיפק לך את המידע הדרוש לך (הנתונים למטה לצורך דוגמא בלבד - אינם הנתונים שלך)?

Number of Prescriptions of Any Antibiotic (Per 100 Visits) for Any Indication

✓ You prescribed 11% below average

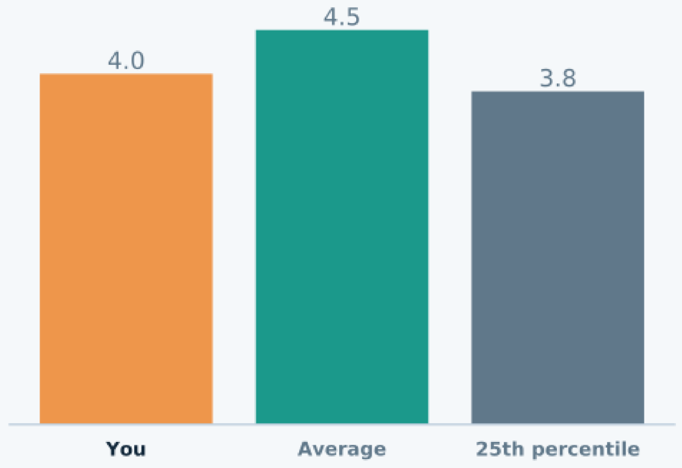

You had 5001 patient visits from Jan 01, 2019 to Dec 31, 2019 .

This figure shows how frequently you prescribed antibiotics for any indication in the previous year (2019). Your prescribing rate (prescriptions per 100 visits) is compared to the average (mean) of your colleagues and participants in Southern Ontario as well as showing your position relative to the 25th percentile. Generally, it is better to be below the average rate of prescribing and within the lowest 25th percentile.

These data are not adjusted for physician's practice characteristics, nor do they differentiate between delayed or non-filled prescriptions. These results are generated from claims data, and misclassification of the diagnosis may be present. As a result, our prescribing targets are referenced to peer benchmarks. Learn more about the data and its interpretation.

- מידה רבה
- במידה מסוימת
- במידה מועטה
- בכלל לא
- לא יודע

שאלה 2: האם במהלך שלושת החודשים האחרונים לקחת בחשבון את תוצאות הדוח הקודם לצורך קבלת החלטה על רישום אנטיביוטיקה למטופל?

- כן
- לא
- לא יודע

שאלה 3: אנא הענק ציון לכל אחד מההיגדים הבאים, בהתאם לתועלת הפוטנציאלית עבורך בדו"ח האישי ששלחנו לך שים לב, לא כל המשפטים/היגדים שאתה נשאל עליהם הופיעו בדו"ח האישי שקבלת. (מועיל מאוד, מועיל במידה מסוימת, מועיל במידה מועטה, כלל לא מועיל, לא יודע)

- אני יכול לראות כיצד דפוס רישום האנטיביוטיקה שלי משתנה לאורך זמן
- אני יכול להשוות את דפוס רישום האנטיביוטיקה שלי לאלה של עמיתיי
- אני יכול לקבל סקירה של דפוסי המרשם המקומיים
- קבלת דוח סקירה מקומי על רמות עמידות
- אני יכול להשוות את דפוס הרישום שלי לעומת הקווים המנחים של הכללית
- מאפשר לי לדון עם עמיתיי על מצב דפוסי המרשם המקומיים

שאלה 4: בסך הכל, איזה ציון היית מעניק לתועלת שאתה מפיק מהדו"ח האישי?

- מועיל מאוד
- מועיל במידה מסוימת
- מועיל במידה מועטה
- כלל לא מועיל
- לא יודע

שאלה 5: אנא הצע דרכים לשיפור הדו"ח האישי (כמו עיצוב הגרף והפרמטרים).

[טקסט חופשי]

שאלה 6: הערות נוספות?

[טקסט חופשי]

Survey 3 (t_6)

שאלה 1: באיזו מידה הפריט הבא - "שיעור מרשמי האנטיביוטיקה שרשמת פר ביקור מטופל בהשוואה לעמיתיך" - סיפק לך את המידע הדרוש לך (הנתונים למטה לצורך דוגמא בלבד - אינם הנתונים שלך)?

Number of Prescriptions of Any Antibiotic (Per 100 Visits) for Any Indication

✓ You prescribed 11% below average

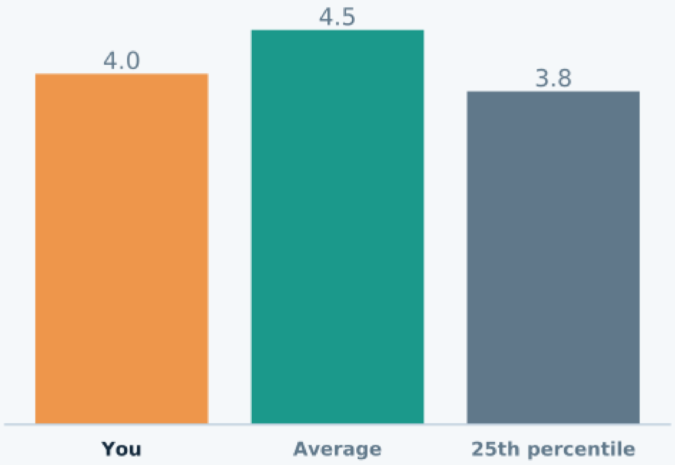

You had 5001 patient visits from Jan 01, 2019 to Dec 31, 2019 .

This figure shows how frequently you prescribed antibiotics for any indication in the previous year (2019). Your prescribing rate (prescriptions per 100 visits) is compared to the average (mean) of your colleagues and participants in Southern Ontario as well as showing your position relative to the 25th percentile. Generally, it is better to be below the average rate of prescribing and within the lowest 25th percentile.

These data are not adjusted for physician's practice characteristics, nor do they differentiate between delayed or non-filled prescriptions. These results are generated from claims data, and misclassification of the diagnosis may be present. As a result, our prescribing targets are referenced to peer benchmarks. Learn more about the data and its interpretation.

- מידה רבה
- במידה מסוימת
- במידה מועטה
- בכלל לא
- לא יודע

שאלה 2: האם במהלך שלושת החודשים האחרונים לקחת בחשבון את תוצאות הדוח הקודם לצורך קבלת החלטה על רישום אנטיביוטיקה למטופל?

- כן
- לא
- לא יודע

שאלה 3: בסך הכל, איזה ציון היית מעניק לתועלת שאתה מפיק מהדו"ח האישי?
- מועיל מאוד
- מועיל במידה מסוימת
- מועיל במידה מועטה
- כלל לא מועיל
- לא יודע

שאלה 4: באופן תיאורטי, מהי התדירות הרצויה לדעתך לקבלת הדו"ח האישי?
- אחת לחודש
- אחת לרבעו
- אחת לחצי שנה
- אחת לשנה
- בכלל לא

שאלה 5: האם מאגר מרכזי של קווים מנחים מקומיים עשוי להועיל לך בעבודתך?
- כן
- לא
- לא יודע

שאלה 6: האם אתה עשוי למצוא תועלת בקבלת גישה לפורטל אישי ברשת האינטרנט, בו תוכל למצוא את דפוסי רישום האנטיביוטיקה האישיים שלך וכן את דפוסי רישום האנטיביוטיקה המקומיים, בצירוף הקווים המנחים הרלוונטיים?
- כן
- לא
- לא יודע

שאלה 7: האם תהיה מוכן להשתתף בראיון עוקב (follow-up), בו תישאל על ניסיונך עם הדו"חות האישיים בצורה מפורטת יותר?
- כן
- לא
- לא יודע

שאלה 8: אנא הצע דרכים לשיפור הדו"ח האישי (כמו עיצוב הגרף והפרמטרים).

[טקסט חופשי]

שאלה 9: הערות נוספות?

[טקסט חופשי]

Appendix 3. OPEN Stewardship feedback survey pilot protocol and interview guide.

OPEN Stewardship Survey: Pilot testing of questionnaires #1, #2 and #3
***** INTERVIEW GUIDE AND TEMPLATE FOR RESPONSES *****

Purpose of the pilots

- To identify issues (clarity, technical errors etc) in the questionnaires and find potential solutions to improve the questionnaires, key points being:
  - Do the respondents understand the purpose of the questionnaire?
  - Do the respondents find the questions relevant?
  - Do the respondents think that there are questions that are missing?
- To get a realistic estimate of the response time.

Before the interview

- **Identify respondents and book time**
- Identify two respondents from each country and sector (humans/companion animals/dairy) who are willing to pilot test the questionnaire, either among those that have been recruited to the OPEN Stewardship study or other similar persons (or a combination of both), do what is most feasible.
- If possible, identify pilot respondents who are geographically close to you so that you can sit next to the respondent when conducting the pilot interview. An interview over phone, Skype or similar is acceptable but a less preferred option.
- If possible, of the two persons per country and sector, select one person with more years with relevant practice and one with less.
- Each pilot respondent will go through two questionnaires: one which is identical to questionnaire #1 and one which includes only those questions that are added to questionnaire #2 and to questionnaire #3 (referred to as pilot questionnaire #2)
  Estimated time for the pilot session is 60 minutes plus introduction and wrap up time, in total up to 90 minutes, so book 1.5 hour for the interview.

- **Communicate with each respondent before the interview**
- Send a (generic) OPEN Stewardship report one week before the booked interview.
- Ask the respondent to spend about 10 minutes to read it at some point before the booked interview.
- Ask the respondent to make a note of the date they read the report.
- Inform the respondent that during the interview you will go through an online questionnaire on his or her computer/device. You will also email some information during the interview. The respondent must thus have internet access plus access to email.

- **Material the interviewer should bring to the interview**
- Laptop or similar, with:
  - This guide in electronic format
  - Draft emails with link to pilot questionnaires to send during the interview
- A printed version of the introductory text that will be sent with each questionnaire
- Pen and paper
- Watch.

During the interview

Use this template during the interview as a guideline for the information that should be given to the respondent. Record the responses to the interview questions in the boxes as specified.
Please fill out and save one template per respondent.
Name each file: COUNTRY_SECTOR_ABBREVIATED-NAME-OF-RESPONDENT (first two letters of given name and first two letters of family name).
You may of course use your own words whenever you wish.

Name of interviewer
Fill out here

Abbreviated name of respondent (first two letters of given name and family name respectively)
Fill out here

- **INTRODUCTION TO INTERVIEW**

Thank you for taking the time to do this interview. As I mentioned previously, I estimate that it will take about 60 minutes.
The aim of the OPEN Stewardship project is to improve appropriate antibiotic prescribing by generating customised reports of individual prescribing in relation to peers. We sent you one of these reports a week ago.
Surveys will be sent out to practitioners enrolled in the OPEN Stewardship intervention at three occasions, to improve the reports.
We are now doing interviews to find out what respondents think about the survey questions. This is to ensure that the surveys measure the right things, so that we will get as good results as possible in order to improve our system. The surveys will be sent out by email and will be filled out online.
Just so you know, I am not the one who have made the survey questions we are about to go through. All of your comments are greatly appreciated.
Approximately 10 people will be interviewed about the survey questions in this pilot. This is an international collaboration, and we work together with colleagues in Canada, Israel, Sweden and the USA [REMOVE YOUR OWN COUNTRY].
If you need a break at any time during the interview just let me know.
Any questions before we get started?

- **SAMPLE REPORT INFORMATION**

Have you had time to read the OPEN Stewardship report?
Interviewer fill out here

- *If not, allow for up to 10 minutes to look at it (but don't rush the person).*

Roughly, when did you read the report? *Or note if the respondent is reading it right now*
Interviewer fill out here

- **EMAIL WITH QUESTIONNAIRE INFORMATION**

So we will start by looking at the text that will accompany the questionnaire, which will be sent out through a link in an email. If you can please read through this email.

- Hand over the paper with the email text. *If the interview is done over the phone, email the text to the respondent now.*

Do you have any comments?
Interviewer fill out here
Is there anything you feel is unclear?
Interviewer fill out here

Do you think the text would motivate someone to answer the survey?
Interviewer fill out here
Any suggestions?
Interviewer fill out here

Thank you!

- **QUESTIONNAIRE 1**

We will now move on to the first questionnaire, and I will email you a link to this questionnaire.

- Email the link to pilot questionnaire 1.

I would like to ask you to fill out the whole questionnaire, but don't press "submit" at the end. Take your time. If you wish you can make notes if there is anything that you come to think of; we will later go through the questionnaire question by question.

- Give the respondent a piece of paper and a pen.
- Respondent fills out the questionnaire.
- Note how long it takes in minutes to complete questionnaire.

Interviewer fill out here

- Note if the respondent seems to spend a longer time reading/re-reading any of the questions.

Interviewer fill out here

Thanks!
So let us start from the beginning with the introduction to the survey.

Do you think this text is informative enough? Any suggestions?
Interviewer fill out here

We will now go through each question, one at a time. I am interested in your opinion, in this situation there is nothing that is right or wrong. So we start with the first question.

- In your discussion with the respondent, note also if anything he or she says that is unexpected/odd.

- **"Is the purpose of the OPEN Stewardship report clear?"**

Can you please describe why you gave this response?
Interviewer fill out here

- **"How informative did you find the following item in the OPEN Stewardship report: Your Prescribing Rate per Visit Compared to Peers"**

Can you please describe why you gave this response?
Interviewer fill out here

Is it clear to you what the various responses mean?
Interviewer fill out here

- **"Was it straightforward how to navigate through the OPEN Stewardship report?"**

Can you please describe why you gave this response?
Interviewer fill out here

- **“Is it useful to include Best Practice Guidelines in the OPEN Stewardship report”**

Can you please describe why you gave this response?
Interviewer fill out here

- **“Rate each of the following items according to its potential usefulness to you in the OPEN Stewardship report.”**
- **“See how my prescribing changes over time”**

Can you please describe why you gave this response?
Interviewer fill out here

- **“Compare my own prescribing with peers”**

Can you please describe why you gave this response?
Interviewer fill out here

- **“Get an overview of local prescribing patterns”**

Can you please describe why you gave this response?
Interviewer fill out here

- **“Compare my own prescribing with guidelines”**

Can you please describe why you gave this response?
Interviewer fill out here

- **“Have discussions with my peers on the local situation … ”**

Can you please describe why you gave this response?
Interviewer fill out here

Is it clear to you what the various responses mean?
Interviewer fill out here

- **“Overall, how would you rate the usefulness of the OPEN Stewardship report?”**

Can you please describe why you gave this response?
Interviewer fill out here

- **"Please suggest how to improve the OPEN Stewardship report:"**
- **"Layout of the graphs (such as colors, size, legends)."**

Is it clear to you what kind of responses could be given here?
Interviewer fill out here

- **"Information included (such as number of graphs, explanatory text)."**

Is it clear to you what kind of responses could be given here?
Interviewer fill out here

- **"Other comments."**

Is it clear to you what kind of responses could be given here?
Interviewer fill out here

I would now like to continue with the layout of the questionnaire.

- Note any potential changes that are indicated by the responses.

What was the most difficult part to navigate through?
Interviewer fill out here

For which questions did you have to use the scroll bar?
Interviewer fill out here

If you now think about the questionnaire as a whole,

What is your overall impression?
Interviewer fill out here

After reading these questions – what would you say is the purpose of this survey?
Interviewer fill out here

Are there any questions that are *not* relevant?
Interviewer fill out here

Are there any questions that should be phrased differently?
Interviewer fill out here

Is any type of question missing?
Interviewer fill out here

What about the length of the survey?
Interviewer fill out here

- **QUESTIONNAIRE 2**

The second and last questionnaire that I want you to go through contains some other questions that we want to ask when evaluating the project and the reports on prescription patterns. It contains 7 questions. I will now email you the link to this questionnaire.

- Email the link to pilot questionnaire 2.

I would like to ask you to now fill out the second questionnaire.

- Respondent fills out the questionnaire.

Again we will now go through each question one at a time.

- **"1. In the past three months, did you ever think about your previous OPEN Stewardship report when making an antibiotic prescribing decision?"**

Can you please describe why you gave this response?
Interviewer fill out here

- **"2. In general, how useful do you think the OPEN Stewardship report would be to other prescribers?"**

Can you please describe why you gave this response?
Interviewer fill out here

Is it clear to you what the various responses mean?
Interviewer fill out here

- **"3. Hypothetically, how often do you think it would be appropriate to receive OPEN Stewardship reports?"**

Can you please describe why you gave this response?
Interviewer fill out here

Is it clear to you what the various responses mean?
Interviewer fill out here

- **"4. Does the pdf format of the OPEN Stewardship report work well for you?"**

Can you please describe why you gave this response?
Interviewer fill out here

- **"5. Would a central repository of local treatment guidelines be useful for your practice?"**

What does this question mean, can you describe it in other words?
Interviewer fill out here

- **"6. Do you think it would be useful to have access to a personalized web-portal where you could browse your own and local patterns of antibiotic use, as well as best practice guidelines?"**

What does this question mean, can you describe it in other words?
Interviewer fill out here

- **“7. Would you be willing to participate in a follow-up interview where we would ask in more detail about your experiences with the OPEN Stewardship reports?”**

Can you please describe why you gave this response?
Interviewer fill out here

- **END OF INTERVIEW**

- Note which kind of device and browser the respondent uses.

Interviewer fill out here

We have now finished. On behalf of me and all my colleagues in the project, I would like to thank you so much for taking the time to help us to improve the evaluation. It has been most useful for our work. If there is anything that you come to think of afterwards, do not hesitate to contact me.

<u>After the interview</u>

Email the filled out templates (in English in case you have used another language for your notes) to the Public Health Agency of Sweden who will compile from all sites. Make sure that the file names are: COUNTRY_SECTOR_ABBREVIATED-NAME-OF-RESPONDENT.
