# Supplementary material for: Evaluation of an automated feedback intervention to improve antimicrobial prescribing among primary care physicians (OPEN Stewardship): protocol for an interrupted time-series and usability analysis in Ontario, Canada and Southern Israel
Source: BMJ Open. 2021 Jan 13;11(1):e039810. doi: 10.1136/bmjopen-2020-039810 (PMC7812099; doi:10.1136/bmjopen-2020-039810)
Supplement: Author's manuscript [file bmjopen-2020-039810.draft_revisions.pdf]

BMJ Open is committed to open peer review. As part of this commitment we make the peer review history of every article we publish publicly available.

When an article is published we post the peer reviewers' comments and the authors' responses online. We also post the versions of the paper that were used during peer review. These are the versions that the peer review comments apply to.

The versions of the paper that follow are the versions that were submitted during the peer review process. They are not the versions of record or the final published versions. They should not be cited or distributed as the published version of this manuscript.

BMJ Open is an open access journal and the full, final, typeset and author-corrected version of record of the manuscript is available on our site with no access controls, subscription charges or pay-per-view fees (http://bmjopen.bmj.com).

If you have any questions on BMJ Open's open peer review process please email info.bmjopen@bmj.com

BMJ Open

Evaluation of an automated feedback intervention to improve antimicrobial prescribing among primary care physicians (OPEN Stewardship): Protocol for an interrupted time-series and usability analysis in Ontario, Canada and Southern Israel

Journal:	*BMJ Open*
Manuscript ID	bmjopen-2020-039810
Article Type:	Protocol
Date Submitted by the Author:	27-Apr-2020

Complete List of Authors:	Soucy, Jean-Paul; University of Toronto Dalla Lana School of Public Health, Division of Epidemiology<br>Low, Marcelo; Clalit Health Services<br>Acharya, Kamal; University of Guelph Ontario Veterinary College, Department of Population Medicine<br>Ellen, Moriah; University of the Negev Guilford Glazer Faculty of Business and Management, Department of Health Services Management<br>Hulth, Anette; Folkhalsomyndigheten<br>Löfmark, Sonja; Folkhalsomyndigheten<br>Garber, Gary; Public Health Ontario, Infection Prevention and Control; University of Ottawa Faculty of Medicine, Medicine/infectious diseases<br>Watson, William; University of Toronto<br>Moran-Gilad, Jacob ; Ben-Gurion University of the Negev Faculty of Health Sciences<br>Fisman, David N.; University of Toronto<br>MacFadden, Derek; Ottawa Hospital Research Institute, Infectious Diseases

Keywords:	INFECTIOUS DISEASES, Public health < INFECTIOUS DISEASES, Epidemiology < INFECTIOUS DISEASES, PRIMARY CARE

SCHOLARONE™
Manuscripts

I, the Submitting Author has the right to grant and does grant on behalf of all authors of the Work (as defined in the below author licence), an exclusive licence and/or a non-exclusive licence for contributions from authors who are: i) UK Crown employees; ii) where BMJ has agreed a CC-BY licence shall apply, and/or iii) in accordance with the terms applicable for US Federal Government officers or employees acting as part of their official duties; on a worldwide, perpetual, irrevocable, royalty-free basis to BMJ Publishing Group Ltd ("BMJ") its licensees and where the relevant Journal is co-owned by BMJ to the co-owners of the Journal, to publish the Work in this journal and any other BMJ products and to exploit all rights, as set out in our licence.

The Submitting Author accepts and understands that any supply made under these terms is made by BMJ to the Submitting Author unless you are acting as an employee on behalf of your employer or a postgraduate student of an affiliated institution which is paying any applicable article publishing charge ("APC") for Open Access articles. Where the Submitting Author wishes to make the Work available on an Open Access basis (and intends to pay the relevant APC), the terms of reuse of such Open Access shall be governed by a Creative Commons licence – details of these licences and which Creative Commons licence will apply to this Work are set out in our licence referred to above.

Other than as permitted in any relevant BMJ Author's Self Archiving Policies, I confirm this Work has not been accepted for publication elsewhere, is not being considered for publication elsewhere and does not duplicate material already published. I confirm all authors consent to publication of this Work and authorise the granting of this licence.

For peer review only

Evaluation of an automated feedback intervention to improve antimicrobial prescribing among primary care physicians (OPEN Stewardship): Protocol for an interrupted time-series and usability analysis in Ontario, Canada and Southern Israel

Jean-Paul R. Soucy[1], Marcelo Low[2, 3], Kamal R. Acharya[4], Moriah E. Ellen[5, 6, 7], Anette Hulth[8,], Sonja Löfmark[8], Gary Garber[9, 10, 11], William Watson[12, 13], Jacob Moran-Gilad[14], David N. Fisman[1]*, Derek R. MacFadden[15]*

* co-senior authors

[1] Division of Epidemiology, Dalla Lana School of Public Health, University of Toronto, Toronto, Canada
[2] Clalit Health Services, Chief Physician's Office, Tel Aviv, Israel
[3] School of Public Health, University of Haifa, Haifa, Israel
[4] Department of Population Medicine, University of Guelph, Guelph, Canada
[5] Department of Health Systems Management, Guilford Glazer Faculty of Business and Management and Faculty of Health Sciences, Ben-Gurion University of the Negev, Beer Sheva, Israel
[6] McMaster Health Forum, McMaster University, Hamilton, Canada
[7] Institute for Health Policy, Management and Evaluation, University of Toronto, Toronto, Canada
[8] Public Health Agency of Sweden, Stockholm, Sweden
[9] Infection Prevention and Control, Public Health Ontario, Toronto, Canada
[10] Department of Medicine, University of Toronto, Toronto, Canada
[11] Department of Medicine, University of Ottawa, Ottawa, Canada
[12] St Michael's Hospital, Toronto, Canada
[13] Department of Family & Community Medicine, University of Toronto, Canada
[14] School of Public Health, Faculty of Health Sciences, Ben-Gurion University of the Negev, Beer Sheva, Israel
[15] Ottawa Hospital Research Institute, Ottawa, Canada

Corresponding author: Derek R. MacFadden (derek.macfadden@gmail.com): Ottawa Hospital Research Institute, 1053 Carling Ave, Ottawa, ON, Canada K1Y 4E9

Word count: 3,151

Keywords: antimicrobial stewardship, antimicrobial resistance, antimicrobial prescribing, primary care, interrupted time-series analysis

For peer review only

ABSTRACT

Introduction

Antimicrobial resistance undermines our ability to treat bacterial infections, leading to longer hospital stays, increased morbidity and mortality, and a mounting burden to the healthcare system. Antimicrobial stewardship is increasingly important to safeguard the efficacy of existing drugs, as few new drugs are in the developmental pipeline. While significant progress has been made with respect to stewardship in hospitals, relatively little progress has been made in the primary care setting, where the majority of antimicrobials are prescribed. OPEN Stewardship is an international collaboration to develop an automated feedback platform to improve responsible antimicrobial prescribing among primary care physicians and capable of being deployed across heterogeneous healthcare settings. We describe the protocol for an evaluation of this automated feedback intervention with two main objectives: assessing changes in antimicrobial prescribing among participating physicians and determining the usability and usefulness of the reports.

Methods and analysis

A non-randomized evaluation of the automated feedback intervention (OPEN Stewardship) will be conducted among approximately 150 primary care physicians recruited from Ontario, Canada and Southern Israel, based on a series of targeted stewardship messages sent using the platform. Using a controlled interrupted time-series analysis, we will compare the antimicrobial prescribing rates of participants before and after the intervention, and also to the prescribing rates of non-participants (from the same healthcare network) during the same period. We will examine outcomes targeted by the stewardship messages, which include prescribing for antimicrobials with duration longer than seven days as well as prescribing for indications where antimicrobials are typically unnecessary. Participants will also complete a series of surveys to determine the usability and usefulness of various elements of the stewardship reports.

Ethics and dissemination

All participating sites have obtained research ethics board approval to recruit providers and access anonymized prescribing data. Dissemination will occur through open-access publication, stakeholder networks, and national/international meetings.

STRENGTH AND LIMITATIONS OF THIS STUDY

- Multinational pragmatic study
- Large number of providers studied with extended follow-up
- Comprehensive evaluation including pre-post design and concurrent controls
- Scalable and open concept with potential for broad generalizability to various care settings
- Differences in recruitment strategy for intervention group across the two study sites may result in heterogeneity of intervention effect

For peer review only

INTRODUCTION

The World Health Organization has called antimicrobial resistance "one of the biggest threats to global health, food security, and development today." [1] Antimicrobial resistance undermines our ability to treat bacterial infections, leading to longer hospital stays, increased complications, higher mortality, and a mounting resource burden on healthcare systems globally [1–5]. It has been projected that resistant microbes could contribute to up to 10 million deaths per year by 2050, greater than the current annual burden of cancer deaths. Additionally, a cumulative loss in global production of trillions of USD in the same time frame is possible [6], hindering efforts to eliminate poverty [7]. Despite a widespread recognition of antimicrobial use as the leading driver of resistance, inappropriate prescribing remains common [8–10]. Given the paucity of new antimicrobials in the drug development pipeline [11], it is critical that we act to safeguard the effectiveness of existing drugs by reducing inappropriate antimicrobial prescribing.

Antimicrobial stewardship refers to the effort to promote the responsible use of antimicrobials and to ensure that patients receive the best choice, duration, and route of antimicrobial treatment, while minimizing potential side effects and the dissemination of antimicrobial resistance [12]. While progress has been made in advancing antimicrobial stewardship in hospitals, leading to significant cost savings and improved patient outcomes [13,14], progress in the primary care setting has been slower [15–17]. This is troubling, given that most antimicrobials are prescribed in the outpatient setting, rather than in hospitals (e.g., over 90% in Canada) [18,19]. Supporting general and family practitioners with prescribing is especially important, since they account for the majority of antimicrobials dispensed for human use in Canada [19]. The United States Centers for Disease Control and Prevention have issued a checklist identifying the key elements to a successful antimicrobial stewardship program in an outpatient setting, highlighting easy access to evidence-based prescribing guidelines and the technical capacity to track, evaluate, and report on antimicrobial prescribing [20]. An online, automated feedback system facilitating these elements would be a major asset to advancing stewardship in primary care.

OPEN Stewardship is an international collaboration with the goal of developing an open, web-based platform to improve antimicrobial prescribing among primary care physicians. This platform integrates with local antimicrobial prescribing data to provide participating physicians with regular, automated feedback (identified by a recent Cochrane review as an effective technique to enable appropriate antimicrobial prescribing in the hospital setting [14]) and connects users with locally relevant prescribing guidelines. The development of a standardized, accessible stewardship platform addresses a key gap for stewardship interventions in the primary care setting, namely the difficulty of expanding across heterogeneous, resource-constrained healthcare systems. As part of a One Health approach recognizing the interconnectedness of human and animal health [21], the platform is also being developed for use in veterinary care, a sector where antimicrobial stewardship has received less attention. A study parallel to the present study will be occurring among veterinary care providers.

A study of the intervention will voluntarily enrol primary care physicians from practices in two study sites: Ontario, Canada and Southern Israel. The intervention will consist of three personalized electronic reports sent to participants over the span of six months, benchmarking their prescribing rate to those of their peers and attaching locally relevant prescribing guidelines for specific indications. The messaging will target antimicrobial prescribing longer than seven days (few conditions require longer than this [22]), as well as two indications in particular: acute sinusitis and viral respiratory conditions. These common infections are a promising target for stewardship [8,20,23,24]. In the US, acute respiratory conditions as a whole account for close to half of antimicrobials prescribed and a great majority of those prescribed inappropriately in the ambulatory care setting [8].

For peer review only

A controlled interrupted time-series design will be used to assess the changes in prescribing among participating physicians compared to both their own prescribing prior to the intervention and to changes in the prescribing of their non-intervention peers in the same network. While overall antimicrobial prescribing is a proxy for our desired measure, which is appropriate antimicrobial prescribing, the microbiological data do not exist to validate the appropriateness of each individual prescription. Nonetheless, given the ubiquity of inappropriate prescribing, particularly for acute respiratory tract infections, any reduction in prescribing as a result of the intervention is likely to be positive [8,25,26]. The design of this study addresses the limitations of some previous studies of stewardship interventions, including small sample sizes, single-centre design, a lack of external controls, and short follow-up [16]. Surveys will be administered to participants to assess the usefulness and usability of the various elements of the reports, as well as soliciting feedback on how to improve the reports for future use.

OBJECTIVES

The aim of this project is to assess the impact of an automated feedback intervention on antimicrobial prescribing in primary care physicians. Specifically, for a cohort of primary care physicians in Ontario, Canada and Southern Israel, we will determine the impact of the stewardship intervention on:

1. The overall antimicrobial prescribing rate in the period following the intervention compared to the period preceding it and to a cohort of primary care physicians who did not receive the intervention.

2. The prescribing rate for antimicrobials with duration longer than seven days in the period following the intervention compared to the period preceding it and to a cohort of primary care physicians who did not receive the intervention.

3. The antimicrobial prescribing rate for acute sinusitis in the period following the intervention compared to the period preceding it and to a cohort of primary care physicians who did not receive the intervention.

4. The antimicrobial prescribing rate for viral respiratory conditions in the period following the intervention compared to the period preceding it and to a cohort of primary care physicians who did not receive the intervention.

We will also assess:

5. The usability and usefulness of each component of the antimicrobial stewardship reports, as well as the usefulness of the reports overall.

METHODS AND ANALYSIS

Objectives 1–4: Non-randomized evaluation of intervention with interrupted time-series analysis

Study design

Primary care physicians from two study sites will be voluntarily enrolled to participate in an antimicrobial stewardship intervention. The providers' rates of antimicrobial prescribing after the intervention will be compared to their rates prior to having received the reports, as well as the

For peer review only

prescribing rates of their peers who did not participate in the intervention. This study design is know as a controlled interrupted time-series [27].

Participants

Approximately 100–150 primary care physicians will be enrolled from two study sites: Ontario, Canada (50) and Southern Israel (50–100). Participants will be drawn from the University of Toronto Practice-Based Research Network (UTOPIAN) and Clalit Health Services (CHS), respectively. UTOPIAN is "one of the largest and most representative primary care research networks in North America" [28], including data for over 400 physicians with an average practice size of 1,018 patients [29]. CHS is the largest healthcare maintenance organization in Southern Israel [30], and covers nearly 80% of the population in Southern Israel. Existing data storage and sharing facilities within both of these networks allow access to de-identified patient data for research in the primary care setting.

In Ontario, primary care providers will be voluntarily enrolled, provided they worked for at least 12 months prior the intervention, expect to continue working for the 12 months following the start of the intervention, and are not actively participating in another stewardship intervention. Providers will be approached for enrolment via email or in-person presentations to groups of providers. In Southern Israel, half of the approximately 400 eligible physicians (average number of patients: 1,482) will be randomized (using random assignment in an Excel spreadsheet by the study coordinator) to be approached (via a letter from the Chief Physician, followed by contact by an interviewer) for voluntary enrolment in the study. Anonymized prescribing data of non-participating physicians within UTOPIAN and CHS will be available as an external control group (approximately 350 for UTOPIAN and 250–300 for CHS, assuming a 25–50% response rate for physicians approached to enrol).

It is possible that physicians who voluntarily enrol in the antimicrobial stewardship intervention will be more engaged with stewardship prior to the intervention, and thus may have less to gain from a stewardship intervention than less-engaged physicians. Differences in the recruitment strategy between sites may result in a heterogeneity in the observed effect, which will be accounted for in the analysis.

Intervention

The intervention will consist of three personalized reports generated by the OPEN Stewardship platform and emailed to participants in the intervention group. The first report will be sent at the beginning of the intervention period (t_0) and will consist of a welcome email followed by a benchmark of the provider's overall antimicrobial prescribing rate relative to the average and 25th percentile of providers in the same network (UTOPIAN or CHS). This report will also include guidelines highlighting that most conditions do not require more than 7 days of antimicrobials and a benchmark of the provider's proportion of antimicrobial prescriptions with duration longer than 7 days relative to the average and 25th percentile of providers in the same network. The two subsequent reports will be sent 3 (t_3) and 6 (t_6) months after the first report. These reports will target acute sinusitis and viral respiratory conditions, respectively, and will consist of a benchmark of the provider's prescribing for the specified indication as well as study site-specific best practice guidelines for the specified indication. The study concludes one year after the first report is sent (t_{12}). The composition of each report is summarized in table 1. An example figure is shown in figure 1.

Outcomes

Each of the 4 objectives corresponds to an outcome:

For peer review only

1. Each provider's overall monthly prescribing rate for antimicrobials, as measured by the number of prescriptions divided by the number of patients visits in that month.
2. The prescribing rate for antimicrobials lasting longer than seven days in duration.
3. The prescribing rate for visits with the indication of acute sinusitis.
4. The prescribing rate for visits with an indication classified as a viral respiratory condition (see table S1 for ICD-9 codes).

Covariates

The analysis will include potential predictors of a provider's baseline prescribing rate, as well as seasonality (table 2). These include a provider's age, sex, and employment status (full-time or part-time) [10] and monthly practice patient composition (by age and sex) [19,31]. We will also use nested random intercepts for provider, practice group (clinic), and study site, representing within-group prescribing norms and a provider's baseline willingness to prescribe after accounting for other predictors, as well as heterogeneity between study sites. Seasonality will be accounted for using a categorical variable for each month compared to January. An interaction term with study site will be included for each covariate to allow for differences in seasonal and demographic prescribing patterns between the two countries in our study.

Statistical analysis

We will perform a multilevel, controlled interrupted time-series analysis with the provider as the unit of analysis [32]. A negative binomial model (to allow for overdispersion of the outcome) will be used with prescribing rates as the outcome. Each provider in the intervention group will contribute 24 observations: a minimum of 12 months in the pre-intervention period (t_{-12} to t_0) and up to 12 months in the post-intervention period, depending on the objective. The pre-intervention and post-intervention periods of each outcome are defined by the time when the relevant report was delivered. The first report (t_0) corresponds to objectives 1 and 2, the second report (t_3) to objective 3, and the third report (t_6) to objective 4. The pre-intervention and post-intervention periods of each objective are summarized in table 3. Providers in the external control group will contribute 24 observations in the control period. The timeline of the study for each objective is shown in figure 2.

The intervention effect will be modelled as a level change and/or change in slope for the prescribing rate at the beginning of the post-intervention period [27]. An interaction term with study site will be included to allow for differences in the intervention effect between Ontario and Southern Israel. Stewardship interventions often result in a reversion to baseline behaviour after the intervention is discontinued [33,34]. To examine the durability of the intervention effect, we will contrast the coefficients estimated from models including 1) all months in the post-intervention period, 2) the first 3 months in the post-intervention period, 3) the first 6 months in the post-intervention period. Finally, since the first report at t_0 (highlighting overall prescribing) is likely to affect objectives targeted by later reports (at t_3 or t_6), for objectives 3 and 4, we will consider t_0 as an alternative start time of the post-intervention period.

Autocorrelation in each provider's time-series should be accounted for by the random intercept (provider nested within practice group nested within study site) and by controlling for seasonality (categorical variable for month) [27]. In case of residual autocorrelation, an autoregressive covariance structure will be considered.

For peer review only

The effect of the COVID-19 pandemic on prescribing in the pre-intervention period will also be considered.

Sensitivity analyses

Alternative parameterizations of the study outcomes will be considered. For the prescribing rate of antimicrobials with courses longer than seven days (objective 2), we will consider mean duration of prescription as an alternative outcome. There may be issues with how visit indication is coded in the electronic medical record, whereas the type of antimicrobial prescribed is very likely to be accurate. A sensitivity analysis will be performed using the prescribing rates (for all visits, ignoring indication) for antimicrobials most often prescribed for viral respiratory conditions (e.g., amoxicillin, amoxicillin/clavulanic acid, azithromycin [35]) as the outcome, rather than the indication-specific prescribing rate of antimicrobials for viral respiratory conditions. Since acute respiratory tract infections make up a large proportion of total antimicrobial prescribing, the signal from the intervention may be evident using these outcomes as well.

To ascertain whether there could be negative effects of the stewardship intervention, we will evaluate (1) the frequency of bacterial pneumonia and pyelonephritis diagnoses and (2) the number of hospital admissions (including those related to infection) in the pre-intervention and post-intervention periods. Admissions data are only available for CHS (Israel), and thus will only be evaluated at this site only.

Including patient composition as predictors (e.g., percentage of patients 65 and older, percentage of female patients) may be insufficient to capture differences in prescribing occurring between strata of age and sex. A sensitivity analysis will be performed where the outcome will be stratified by age group and sex and modelled separately. Although stratification increases the noise in each individual time-series, these models may reveal differences in the effectiveness of the intervention across sex/age groups, which have different prescribing patterns.

As the offer to enrol in the study is randomized at one study site (Southern Israel), an intention-to-treat analysis will be performed as a supplementary analysis at this site only.

Sample size

We provide an approximate sample size calculation for a difference in antimicrobial prescribing between two groups (intervention and control) in a Poisson framework using the method described by Whitehead [36] and implemented in an online calculator (http://www.obg.cuhk.edu.hk/ResearchSupport/StatTools/SSiz2Counts_Tab.php). In Fleming-Dutra's [8] study of outpatient prescribing, 12.6% of visits resulted in an antimicrobial prescription. Since most visits would only results in a single antimicrobial prescription, we take this as the baseline prescribing rate (0.126/visit). The American Academy of Family Physicians estimated in 2012 that their members had an average of 92 patient encounters per week (368 per month) [37]. We define a 15% reduction in prescribing rate as clinically meaningful (0.107/visit). To achieve a power of 90% (with $\alpha = 0.05$, one-tail), we would require approximately 15 physicians in each group to be observed for 1 month each, which is much smaller than our expected sample size at each study site. While the clustering and likely over-dispersion of prescribing rates in our sample would decrease our power, the fact that we will have 24 months of observations for each provider (rather than 1 month), as well as pre-intervention and post-intervention observations, greatly enhances the power of our study to detect an intervention effect.

Objective 5: Survey of usability among participating providers

For peer review only - http://bmjopen.bmj.com/site/about/guidelines.xhtml

Study design

Physicians enrolled in the antimicrobial stewardship intervention will receive three short surveys to assess the usability of the reports received and to provide feedback on the intervention.

Surveys

Surveys will be delivered by email to participants in the intervention group 3 days after the initial receipt of each report using Qualtrics (Qualtrics, Provo, UT) in Southern Israel and RedCAP [38] survey software in Ontario (due to differences in institutional policies regarding data collection). The report will be re-sent with the survey and the recipient will be given 7 days to respond. If the recipient fails to respond within this allotted time, the survey and report are re-sent again, giving the recipient an additional 7 days to respond. The first two surveys focus on the usability and usefulness of the emailed reports, with additional space for free text feedback. The final report adds additional questions to assess if the participant thinks the reports would be useful for his or her peers, how often they would prefer to receive reports, and whether particular additional features would be of use to him or her. Survey questions are given in English in Appendix 1; translated questions in Hebrew are given in Appendix 2. Questions were developed in accordance with experts at the Public Health Agency of Sweden. Questions were pilot tested and revised according to the protocol and interviewer guide in Appendix 3.

Statistical analysis

Participants' attitudes regarding the overall usefulness of the intervention and the informativeness of the figure included in each intervention (table 1) will be assessed in each survey and compared longitudinally with a repeated measures ANOVA. Responses to other variables may be analysed using ordinal regression and/or multinomial regression.

ETHICS AND DISSEMINATION

All participating sites in this study have obtained research ethics board approval to recruit providers and access prescribing data. All patient-level prescribing data will be anonymized prior to receipt, available only as the rate of antimicrobial prescribing per visit, stratified by month and indication (and potentially sex and/or broad age category). Study data will be stored on a secure server and accessible only to study analysts. Study participants from Ontario will be compensated with a gift card. Study participants from Israel will be encouraged to participate by a letter from the Chief Physician's Office.

The OPEN Stewardship project includes collaborators affiliated with hospitals, universities, and public health authorities in four countries. In addition to dissemination through stakeholder networks, the results of this study will be published in an open-access journal and presented at national and/or international meetings.

PATIENT AND PUBLIC INVOLVEMENT

Primary care physicians were involved in the conception and design of this study, as well as the study surveys. They were also consulted to assess the appropriate time burden and adequate compensation for participants in the study.

For peer review only

REFERENCES

1 World Health Organization. Antibiotic resistance. 2019. http://www.who.int/mediacentre/factsheets/antibiotic-resistance/en/ (accessed 1 Jan 2019).

2 The Review on Antimicrobial Resistance. Tackling Drug-resistant Infections Globally: Final Report and Recommendations. 2016. https://amr-review.org/sites/default/files/160518_Final%20paper_with%20cover.pdf (accessed 27 May 2018).

3 World Health Organization. GLASS | Global antimicrobial resistance surveillance system (GLASS) report. Geneva: 2017.

4 Interagency Coordination Group on Antimicrobial Resistance. No Time to Wait: Securing the future from drug-resistant infections. Interagency Coordination Group on Antimicrobial Resistance 2019. https://www.who.int/antimicrobial-resistance/interagency-coordination-group/IACG_final_report_EN.pdf (accessed 18 Jul 2019).

5 Roberts RR, Hota B, Ahmad I, *et al.* Hospital and Societal Costs of Antimicrobial-Resistant Infections in a Chicago Teaching Hospital: Implications for Antibiotic Stewardship. *Clin Infect Dis* 2009;**49**:1175–84.

6 The Review on Antimicrobial Resistance. Antimicrobial Resistance: Tackling a Crisis For the Health and Wealth Of Nations. 2014.

7 Ahmed SA, Bariş E, Go DS, *et al.* Assessing the Global Economic and Poverty Effects of Antimicrobial Resistance. 2017.

8 Fleming-Dutra KE, Hersh AL, Shapiro DJ, *et al.* Prevalence of inappropriate antibiotic prescriptions among US ambulatory care visits, 2010-2011. *JAMA* 2016;**315**:1864–73.

9 Shively NR, Buehrle DJ, Clancy CJ, *et al.* Prevalence of Inappropriate Antibiotic Prescribing in Primary Care Clinics within a Veterans Affairs Health Care System. *Antimicrob Agents Chemother* 2018;**62**:e00337-18.

10 Cadieux G, Tamblyn R, Dauphinee D, *et al.* Predictors of inappropriate antibiotic prescribing among primary care physicians. *Can Med Assoc J* 2007;**177**:877–83.

11 Renwick MJ, Simpkin V, Mossialos E. Targeting innovation in antibiotic drug discovery and development: the need for a one health - one Europe - one world framework. 2016. http://www.ncbi.nlm.nih.gov/books/NBK447337/ (accessed 18 Feb 2020).

12 Dyar OJ, Huttner B, Schouten J, *et al.* What is antimicrobial stewardship? *Clin Microbiol Infect* 2017;**23**:793–8.

13 Nathwani D, Varghese D, Stephens J, *et al.* Value of hospital antimicrobial stewardship programs [ASPs]: a systematic review. *Antimicrob Resist Infect Control* 2019;**8**.

14 Davey P, Marwick CA, Scott CL, *et al.* Interventions to improve antibiotic prescribing practices for hospital inpatients. *Cochrane Database Syst Rev* Published Online First: 9 February 2017.

For peer review only

15 Bal AM, Gould IM. Antibiotic stewardship: Overcoming implementation barriers. *Curr Opin Infect Dis* 2011;**24**:357–62.

16 Allerberger F, Gareis R, Jindrák V, *et al.* Antibiotic stewardship implementation in the EU: the way forward. *Expert Rev Anti Infect Ther* 2009;**7**:1175–83.

17 Langford BJ, Schwartz KL, Garber GE. Antimicrobial stewardship by family physicians. *Can Fam Physician* 2018;**64**:168–9.

18 Drekonja D, Filice G, Greer N, *et al. Antimicrobial Stewardship Programs in Outpatient Settings: A Systematic Review*. Washington DC: Department of Veterans Affairs 2014. http://www.ncbi.nlm.nih.gov/books/NBK274571/ (accessed 19 Jul 2019).

19 Public Health Agency of Canada. Canadian Antimicrobial Resistance Surveillance System – 2017 Report. 2017.

20 Sanchez GV, Fleming-Dutra KE, Roberts RM, *et al.* Core Elements of Outpatient Antibiotic Stewardship. *MMWR Recomm Rep* 2016;**65**:1–12.

21 McEwen SA, Collignon PJ. Antimicrobial Resistance: a One Health Perspective. In: Schwarz S, Cavaco LM, Shen J, eds. *Antimicrobial Resistance in Bacteria from Livestock and Companion Animals*. Washington DC: ASM Press 2018. 521–47.

22 Pouwels KB, Hopkins S, Llewelyn MJ, *et al.* Duration of antibiotic treatment for common infections in English primary care: cross sectional analysis and comparison with guidelines. *BMJ* 2019;**364**:l440.

23 Smith SM, Fahey T, Smucny J, *et al.* Antibiotics for acute bronchitis. *Cochrane Database Syst Rev* Published Online First: 19 June 2017.

24 Little P, Stuart B, Francis N, *et al.* Effects of internet-based training on antibiotic prescribing rates for acute respiratory-tract infections: a multinational, cluster, randomised, factorial, controlled trial. *The Lancet* 2013;**382**:1175–82.

25 Meeker D, Linder JA, Fox CR, *et al.* Effect of Behavioral Interventions on Inappropriate Antibiotic Prescribing Among Primary Care Practices: A Randomized Clinical Trial. *JAMA* 2016;**315**:562.

26 Groth A, Enoksson F, Hermansson A, *et al.* Acute mastoiditis in children in Sweden 1993–2007—No increase after new guidelines. *Int J Pediatr Otorhinolaryngol* 2011;**75**:1496–501.

27 Bernal JL, Cummins S, Gasparrini A. Interrupted time series regression for the evaluation of public health interventions: a tutorial. *Int J Epidemiol* 2017;**46**:348–55.

28 University of Toronto Practice-Based Research Network. About UTOPIAN. 2017. http://www.dfcm.utoronto.ca/about-utopian (accessed 17 Feb 2019).

29 University of Toronto Practice-Based Research Network. UTOPIAN Data Safe Haven. 2019. http://www.dfcm.utoronto.ca/utopian-data-safe-haven (accessed 4 Sep 2019).

For peer review only

30 Clalit. The Story of Clalit Health Services. 2010. http://www.clalit-global.co.il/en/_the_story_of_clalit_health_services.html (accessed 21 Jul 2019).

31 Dosh SA, Hickner JM, Mainous AGI, *et al.* Predictors of Antibiotic Prescribing for Nonspecific Upper Respiratory Infections, Acute Bronchitis, and Acute Sinusitis. *J Fam Pract* 2000;**49**:409–15.

32 Taljaard M, McKenzie JE, Ramsay CR, *et al.* The use of segmented regression in analysing interrupted time series studies: an example in pre-hospital ambulance care. *Implement Sci* 2014;**9**.

33 Arnold SR, Straus SE. Interventions to improve antibiotic prescribing practices in ambulatory care. *Cochrane Database Syst Rev* Published Online First: 2005.

34 Gerber JS, Prasad PA, Fiks AG, *et al.* Durability of Benefits of an Outpatient Antimicrobial Stewardship Intervention After Discontinuation of Audit and Feedback. *JAMA* 2014;**312**:2569–70.

35 Ebell MH, Radke T. Antibiotic Use for Viral Acute Respiratory Tract Infections Remains Common. *Am J Manag Care* 2015;**21**:9.

36 Whitehead J. *The design and analysis of sequential clinical trials*. Rev. 2nd ed. Chichester, UK: John Wiley & Sons 1997.

37 American Academy of Family Physicians. AAFP Member Survey Provides Valuable Perspective. 2017. https://www.aafp.org/news/blogs/inthetrenches/entry/20170801ITT_Survey.html (accessed 27 Feb 2020).

38 Harris PA, Taylor R, Thielke R, *et al.* Research electronic data capture (REDCap)—A metadata-driven methodology and workflow process for providing translational research informatics support. *J Biomed Inform* 2009;**42**:377–81.

AUTHORS' CONTRIBUTIONS

DRM, MEE, AH, SL, DNF, and J-PRS conceived of the study and the analysis. J-PRS wrote the protocol and will conduct the data analysis. All authors contributed to the protocol and reviewed the final manuscript.

FUNDING STATEMENT

This work was supported by the European Commission's Joint Programming Initiative on Antimicrobial Resistance (5th Joint Call).

COMPETING INTERESTS STATEMENT

The authors declare no conflicts of interest.

For peer review only

Tables

Table 1. Composition of the feedback reports (intervention) at each time point.

Feedback report	Content of report	Benchmarking figure	Guidelines
Report at t_0	1. Antimicrobial prescribing rate/100 visits in the previous year for all indications.<br>2. Proportion of antimicrobial prescriptions with duration over 7 days.	Provider's own rate/proportion versus average rate/proportion and 25th percentile rate/proportion across all providers at the same study site.	Guidelines indicating that antimicrobial prescribing longer than 7 days is not necessary for many conditions.
Report at t_3	1. Antimicrobial prescribing rate/100 visits in the previous year for acute sinusitis.	Provider's own rate versus average rate and 25th percentile rate across all providers at the same study site.	Prescribing guidelines for acute sinusitis.
Report at t_6	1. Antimicrobial prescribing rate/100 visits in the previous year for viral respiratory conditions.	Provider's own rate versus average rate and 25th percentile rate across all providers at the same study site.	Prescribing guidelines for viral respiratory conditions.

For peer review only

Table 2. Covariates included in the interrupted time-series analysis of the OPEN Stewardship study.

Variable	Purpose	Form	Description
Number of prescriptions	Outcome	Integer	Number of antimicrobial prescriptions for given indication
Number of visits for indication	Offset	Integer	Number of patient visits for given indication
Study site	Covariate	Binary	Southern Israel versus Ontario, Canada (baseline)
Practice group	Random intercept	ID	Random intercept for practice group (nested within region)
Provider	Random intercept	ID	Random intercept for provider (nested within practice group)
Month	Covariate	Categorical	Calendar month (baseline = January)
Month × study site	Interaction	Interaction	Allows different value for coefficients for Southern Israel
Physician age	Covariate	Integer	Physician age at t_{-12}
Physician age × study site	Interaction	Interaction	Allows different value for coefficient for Southern Israel
Physician sex	Covariate	Binary	Female versus male (baseline)
Physician sex × study site	Interaction	Interaction	Allows different value for coefficient for Southern Israel
Physician employment status	Covariate	Binary	Part-time versus full-time (baseline)
Physician employment status × study site	Interaction	Interaction	Allows different value for coefficient for Southern Israel
Percent female patients	Covariate	Continuous	Percentage of visits for indication by female patients
Percent female patients × study site	Interaction	Interaction	Allows different value for coefficient for Southern Israel
Percent patients 17 and under	Covariate	Continuous	Percentage of visits for indication by patients 17 and younger
Percent patients 17 and under × study site	Interaction	Interaction	Allows different value for coefficient for Southern Israel
Percent patients 65 and older	Covariate	Continuous	Percentage of visits for indication by patients 65 and older
Percent patients 65 and older × study site	Interaction	Interaction	Allows different value for coefficient for Southern Israel
Post-intervention	Intervention	Binary	1 if provider is in intervention group and intervention has occurred, 0 otherwise; level change of the outcome associated with intervention
Post-intervention × study site	Interaction	Interaction	Allows different value for level change of the outcome for Southern Israel
Time	Trend	Integer	Time since entry into the study (t_{-12}); slope of the time trend of the outcome
Time × study site	Interaction	Interaction	Allows different value for slope of the time trend of the outcome for Southern Israel
Time × post-intervention	Intervention effect	Interaction	Change in slope of time trend in the intervention group after intervention is received
Time × post-intervention × study site	Interaction	Interaction	Allows different value for change in slope of time trend in the intervention group after intervention is received for Southern Israel

For peer review only

Table 3. Timing of analyses for objective 1–4 of the study. The definitions of the pre-intervention and post-intervention periods are dependant on the delivery time of the report (intervention) related to each objective.

Objective	Outcome	Unit of analysis	Intervention time	Number of observations in intervention cohort		Total observations/ observations in control cohort
				Pre-intervention	Post-intervention	
1	Prescribing for all indications	Provider	t_0	12	12	24
2	Prescribing (for all indications) with duration longer than 7 days	Provider	t_0	12	12	24
3	Prescribing for acute sinusitis	Provider	t_3	15	9	24
4	Prescribing for viral respiratory conditions	Provider	t_6	18	6	24

For peer review only

Figure legends

Figure 1. Example of chart sent in the first feedback report, representing the provider's prescribing rate/100 visits for any indication versus the average rate and 25th percentile rate across all providers in the study from the same study site.

Figure 2. Pre-intervention and post-intervention periods for objectives 1–4 of the OPEN Stewardship study. A: Objective 1, overall prescribing (report sent at t_0). B: Objective 2, prescribing with duration longer than 7 days (report sent at t_0). C: Objective 3, prescribing for acute sinusitis (report sent at t_3). D: Objective 4, prescribing for viral respiratory conditions (report sent at t_6). t_0 represents the time of the first report (intervention); t_{-12} represents 1 year prior to the first report; t_{12} marks the conclusion of the study period (1 year after the first report).

For peer review only

Number of Prescriptions of Any Antibiotic (Per 100 Visits) for Any Indication

You prescribed 18% more than average

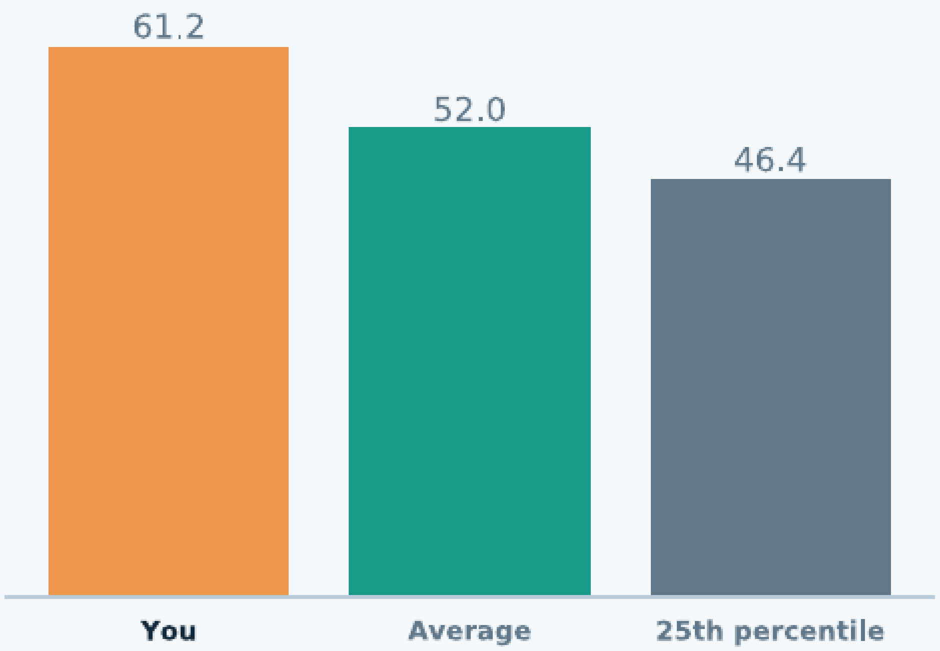

You had 242 patient visits from Jan 01, 2019 to Dec 31, 2019 .

These data are not adjusted for physician's practice characteristics, nor do they differentiate between delayed or non-filled prescriptions. These results are generated from claims data, and misclassification of the diagnosis may be present. As a result, our prescribing targets are referenced to peer benchmarks. Learn more about the data and its interpretation.

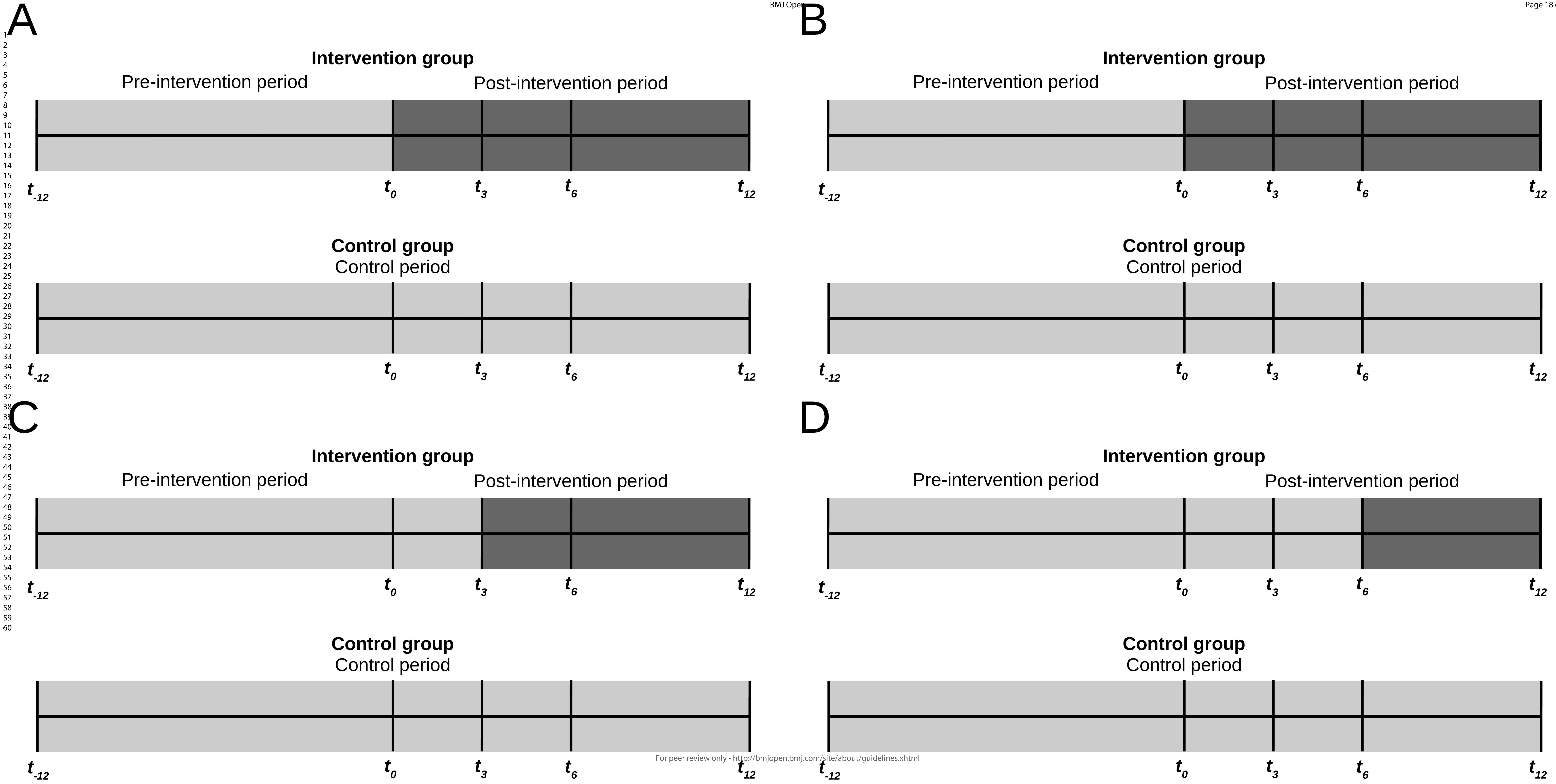
A
Intervention group
Pre-intervention period
Post-intervention period
t-12 t0 t3 t6 t12
Control group
Control period
t-12 t0 t3 t6 t12
B
Intervention group
Pre-intervention period
Post-intervention period
t-12 t0 t3 t6 t12
Control group
Control period
t-12 t0 t3 t6 t12
C
Intervention group
Pre-intervention period
Post-intervention period
t-12 t0 t3 t6 t12
Control group
Control period
t-12 t0 t3 t6 t12
D
Intervention group
Pre-intervention period
Post-intervention period
t-12 t0 t3 t6 t12
Control group
Control period
t-12 t0 t3 t6 t12

Table S1. ICD-9 codes for indications identified as acute sinusitis and viral respiratory conditions in the OPEN Stewardship platform.

OPEN Stewardship category	Indication	ICD-9 codes
Acute sinusitis	Acute sinusitis	461 Acute sinusitis
Viral respiratory conditions	Asthma/allergy	477 Allergic rhinitis, 493 Asthma, 995.3 Allergy, unspecified
	Bronchitis/bronchiolitis	466 Acute bronchitis and bronchiolitis, 490 Bronchitis, not specified as acute or chronic, 492 Emphysema, 496 Chronic airway obstruction, not elsewhere classified
	Influenza	487 Influenza, 488 Influenza due to identified avian influenza virus
	Viral pneumonia	480 Viral pneumonia
	Viral upper respiratory tract infection	460 Acute nasopharyngitis [common cold], 464 Acute laryngitis and tracheitis, 465 Acute upper respiratory infections of multiple or unspecified sites, 786.2 Cough

For peer review only

Appendix 1. Survey questions for participants in the OPEN Stewardship trial (English).

Introductory text: Thank you for agreeing to receive the OPEN Stewardship report on antibiotic prescribing. Help us improve future reports by filling out this short survey. It will only take 3-5 minutes. Questions are multiple choice with the option to provide comments at the end of the survey. Your feedback is important to us and your opinion matters!

Survey 1 (t_0)

Q1. Is the purpose of the OPEN Stewardship report clear?

- Yes
- No
- Don't know

Q2. How informative did you find the following item in the OPEN Stewardship report: Your Prescribing Rate per Visit Compared to Peers (example below).

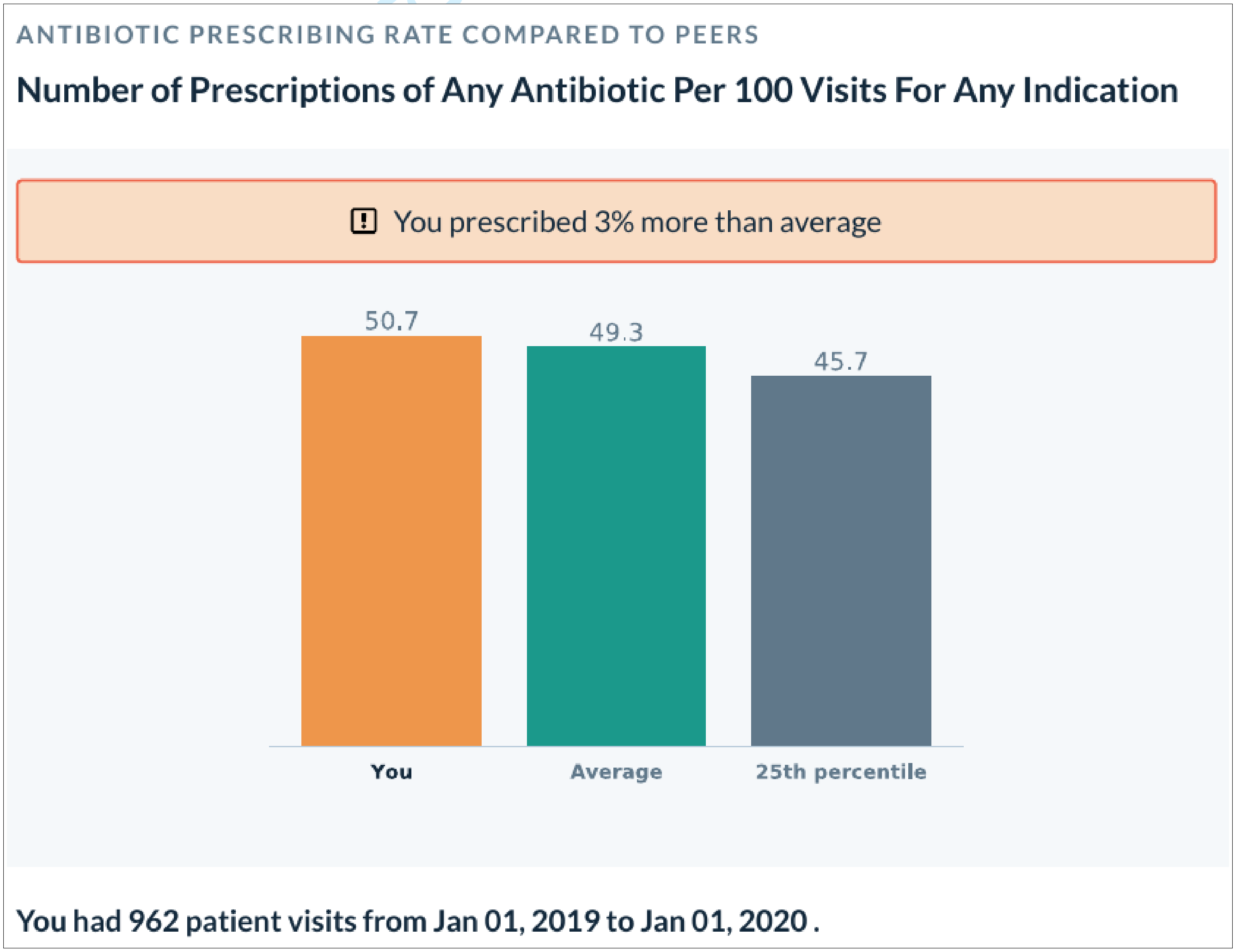

- Very informative
- Somewhat informative

- Not so informative
- Not at all informative
- Don't know

Q3. Was it straightforward how to navigate through the OPEN Stewardship report?

- Yes
- No
- Don't know

Q4. Is it useful to include *Best Practice Guidelines* in the OPEN Stewardship report (sample from most recent report below)?

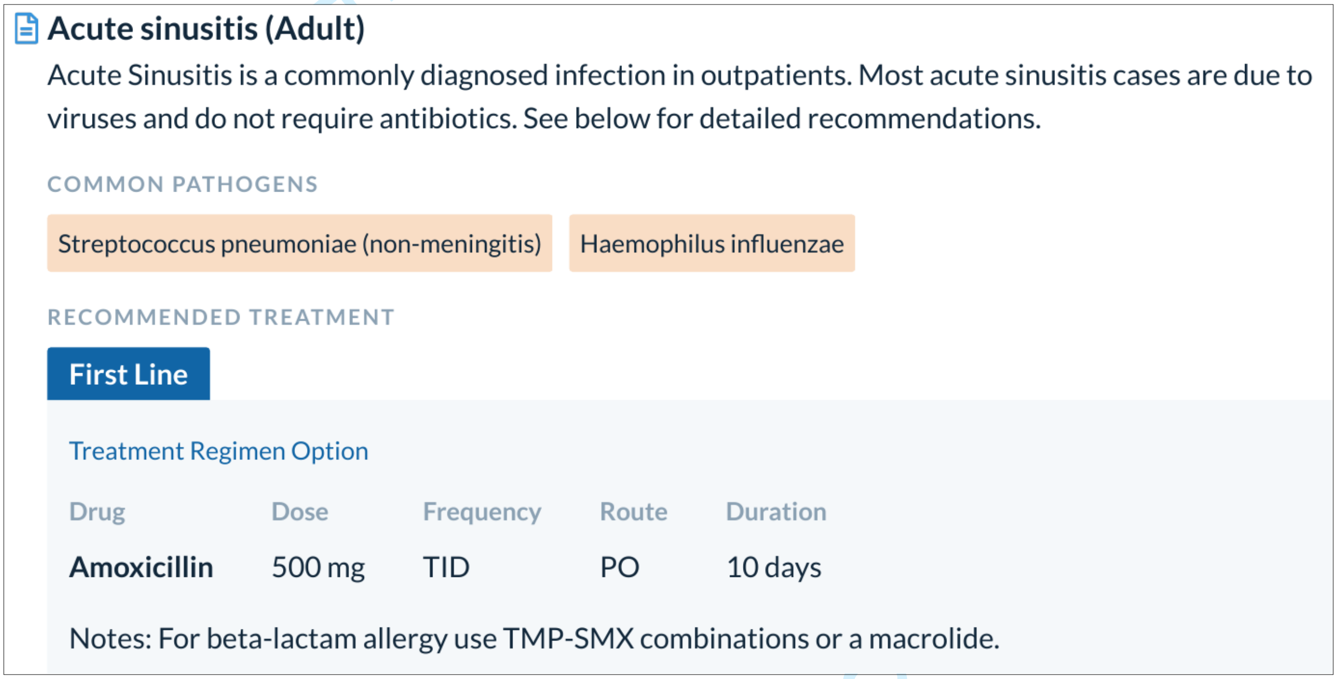

Acute sinusitis (Adult)

Acute Sinusitis is a commonly diagnosed infection in outpatients. Most acute sinusitis cases are due to viruses and do not require antibiotics. See below for detailed recommendations.

COMMON PATHOGENS

Streptococcus pneumoniae (non-meningitis) | Haemophilus influenzae

RECOMMENDED TREATMENT

First Line

Treatment Regimen Option

Drug	Dose	Frequency	Route	Duration
**Amoxicillin**	500 mg	TID	PO	10 days

Notes: For beta-lactam allergy use TMP-SMX combinations or a macrolide.

- Yes
- No
- Don't know

Q5. Rate each of the following items according to its potential usefulness to you in the OPEN Stewardship report. (Very useful, Somewhat useful, Not so useful, Not at all useful, Don't know)

- See how my prescribing changes over time
- Compare my own prescribing with peers
- Get an overview of local prescribing patterns
- Compare my own prescribing with guidelines
- Have discussions with peers on the local situation regarding prescribing patterns

Q6. Overall, how would you rate the usefulness of the OPEN Stewardship report?

- Very useful

- Somewhat useful
- Not so useful
- Not at all useful
- Don’t know

Q7. Please suggest how to improve the OPEN Stewardship report:

1. Layout of the graphs (such as colors, size, legends).

[Free text box]

2. Information included (such as number of graphs, explanatory text).

[Free text box]

3. Other comments.

[Free text box]

Survey 2 (t_3)

Q1. How informative did you find the following item in the OPEN Stewardship report: Your Prescribing Rate per Visit Compared to Peers (example below).

For peer review only

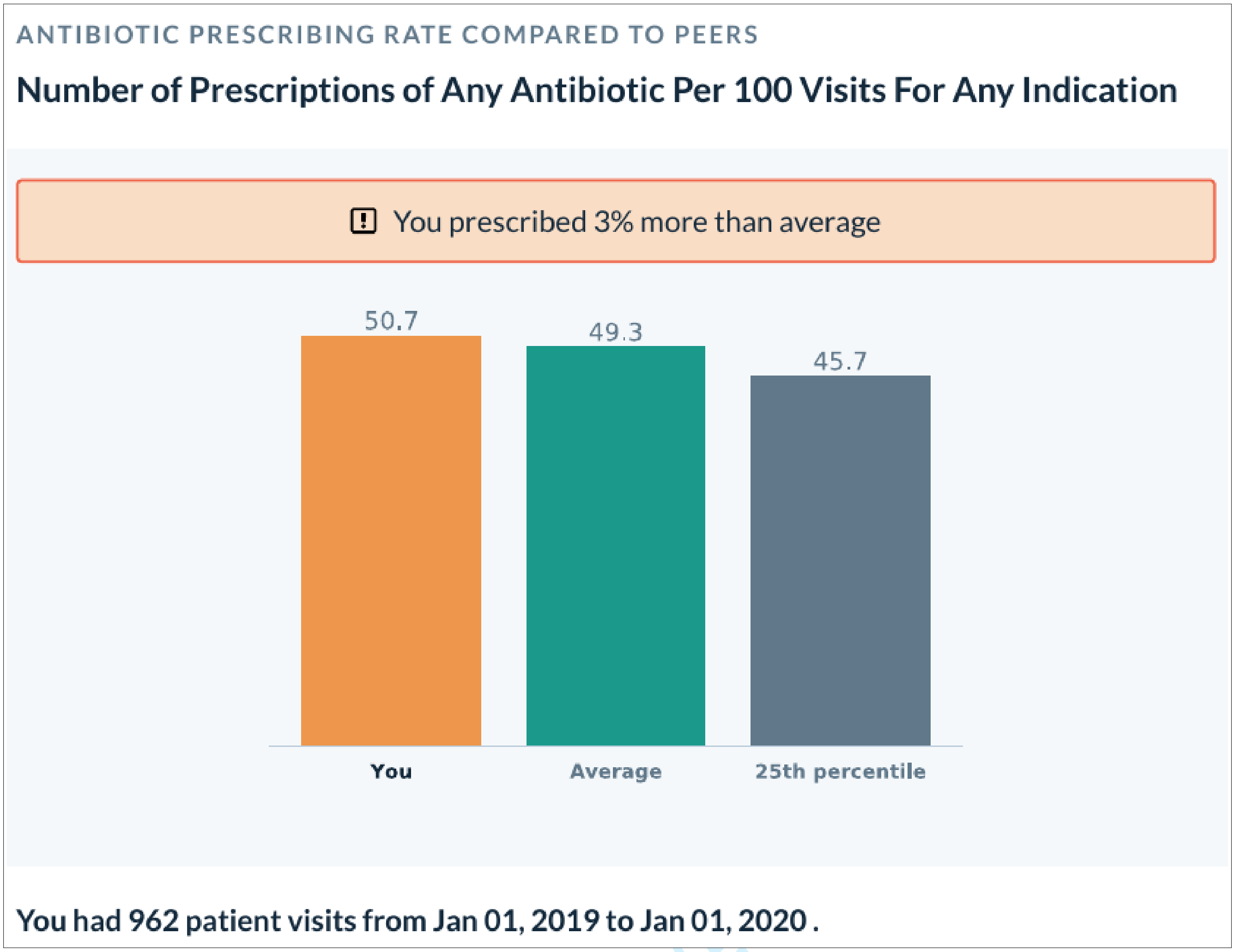

- Very informative
- Somewhat informative
- Not so informative
- Not at all informative
- Don't know

Q2. In the past three months, did you ever think about your previous OPEN Stewardship report when making an antibiotic prescribing decision?

- Yes
- No
- Don't know

Q3. Rate each of the following items according to its potential usefulness to you in the OPEN Stewardship report. (Very useful, Somewhat useful, Not so useful, Not at all useful, Don't know)

- See how my prescribing changes over time
- Compare my own prescribing with peers
- Get an overview of local prescribing patterns
- Compare my own prescribing with guidelines

- Have discussions with peers on the local situation regarding prescribing patterns

Q4. Overall, how would you rate the usefulness of the OPEN Stewardship report?

- Very useful
- Somewhat useful
- Not so useful
- Not at all useful
- Don't know

Q5. Please suggest how to improve the OPEN Stewardship report:

1. Layout of the graphs (such as colors, size, legends).

[Free text box]

2. Information included (such as number of graphs, explanatory text).

[Free text box]

3. Other comments.

[Free text box]

Survey 3 (t_6)

Q1. How informative did you find the following item in the OPEN Stewardship report: Your Prescribing Rate per Visit Compared to Peers (example below).

For peer review only

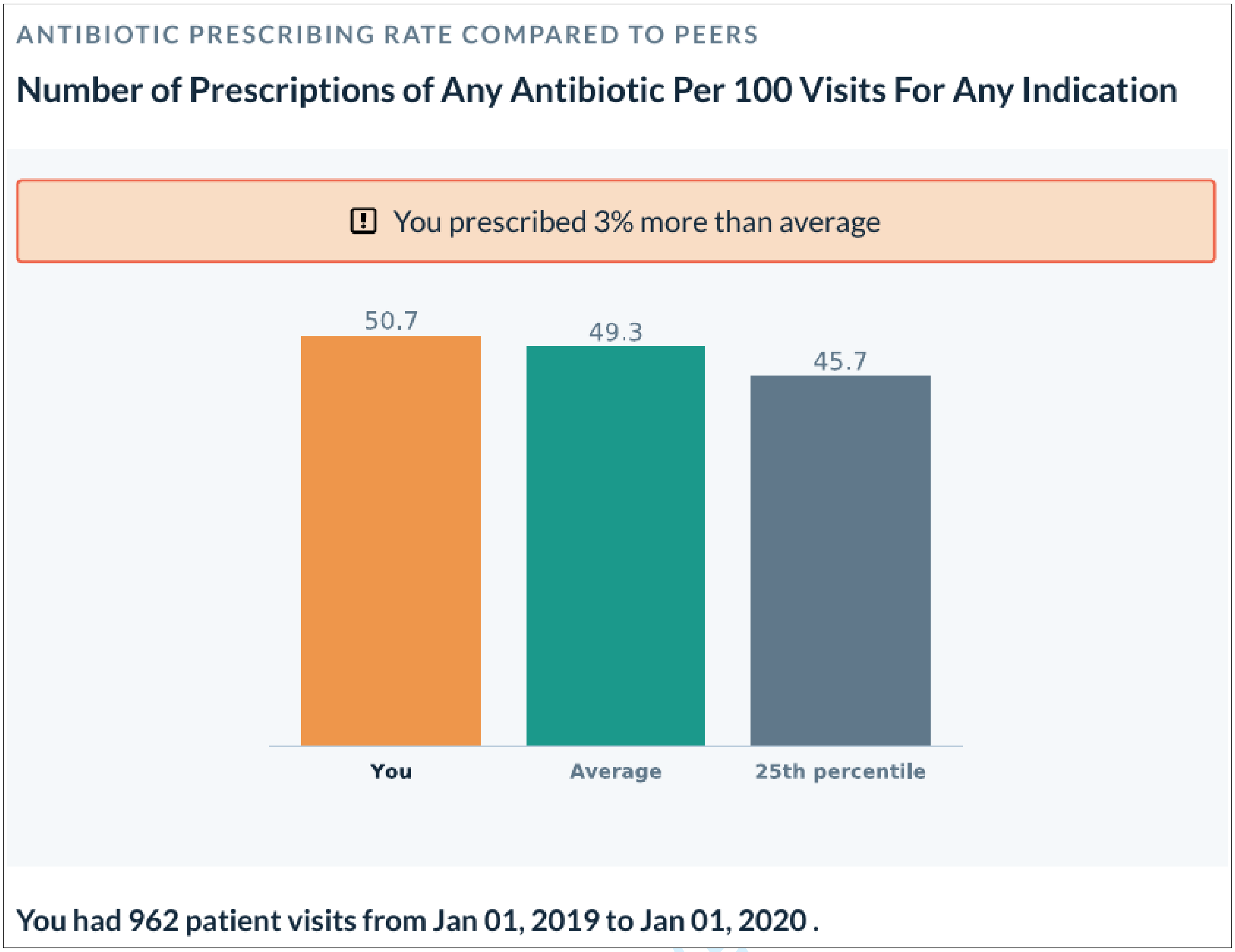

- Very informative
- Somewhat informative
- Not so informative
- Not at all informative
- Don’t know

Q2. In the past three months, did you ever think about your previous OPEN Stewardship report when making an antibiotic prescribing decision?

- Yes
- No
- Don’t know

Q3. Overall, how would you rate the usefulness of the OPEN Stewardship report?

- Very useful
- Somewhat useful
- Not so useful
- Not at all useful
- Don’t know

For peer review only - http://bmjopen.bmj.com/site/about/guidelines.xhtml

Q4. In general, how useful do you think the OPEN Stewardship report would be to other prescribers?

- Very useful
- Somewhat useful
- Not so useful
- Not at all useful
- Don’t know

Q5. Hypothetically, how often do you think it would be appropriate to receive OPEN Stewardship reports?

- Monthly
- Every two months
- Quarterly
- Annually
- Not at all
- Don’t know

Q6. Does the pdf format of the OPEN Stewardship report work well for you?

- Yes
- No
- Don’t know

Q7. Would a central repository of local treatment guidelines be useful for your practice?

- Yes
- No
- Don’t know

Q8. Do you think it would be useful to have access to a personalized web-portal where you could browse your own and local patterns of antibiotic use, as well as best practice guidelines?

- Yes
- No
- Don’t know

Q9. Would you be willing to participate in a follow-up interview where we would ask in more detail about your experiences with the OPEN Stewardship reports? If so, please send an email to [study email address].

Q10. Please suggest how to improve the OPEN Stewardship report:

1. Layout of the graphs (such as colors, size, legends).

[Free text box]

2. Information included (such as number of graphs, explanatory text).

For peer review only

[Free text box]

3. Other comments.

[Free text box]

For peer review only

Appendix 2. Survey questions for participants in the OPEN Stewardship trial (Hebrew).

Introductory text:

רופא יקר,

אנו מודים לך שוב על הסכמתך לקחת חלק במחקר המשותף של אגף רפואה בחטיבת הקהילה בשירותי בריאות כללית והמחלקה לניהול מערכות בריאות באוניברסיטת בן גוריון בנגב בנושא "דפוסי רישום תרופות אנטיביוטיות על-ידי רופאי משפחה".

נודה לך אם תוכל להקדיש שלוש עד חמש דקות מזמנך כדי לענות על השאלון שלהלן, המתייחס לדו"ח האישי שנשלח אליך לאחרונה בנושא מרשמי האנטיביוטיקה של מטופליך. תשובותיך יעזרו לנו לשפר את אופן העברת המידע לרופאים בהמשך.

השאלון נכתב בלשון זכר מטעמי נוחות, אך מיועד לשני המינים.

Survey 1 (t_0)

שאלה 1: האם מטרת הדו"ח האישי ששלחנו לך, ברורה, לדעתך?

- כן
- לא
- לא יודע

שאלה 2: באיזו מידה הפריט הבא – "שיעור מרשמי האנטיביוטיקה שרשמת פר ביקור מטופל בהשוואה לעמיתיך" - סיפק לך את המידע הדרוש לך? (ראה דוגמה להלן)

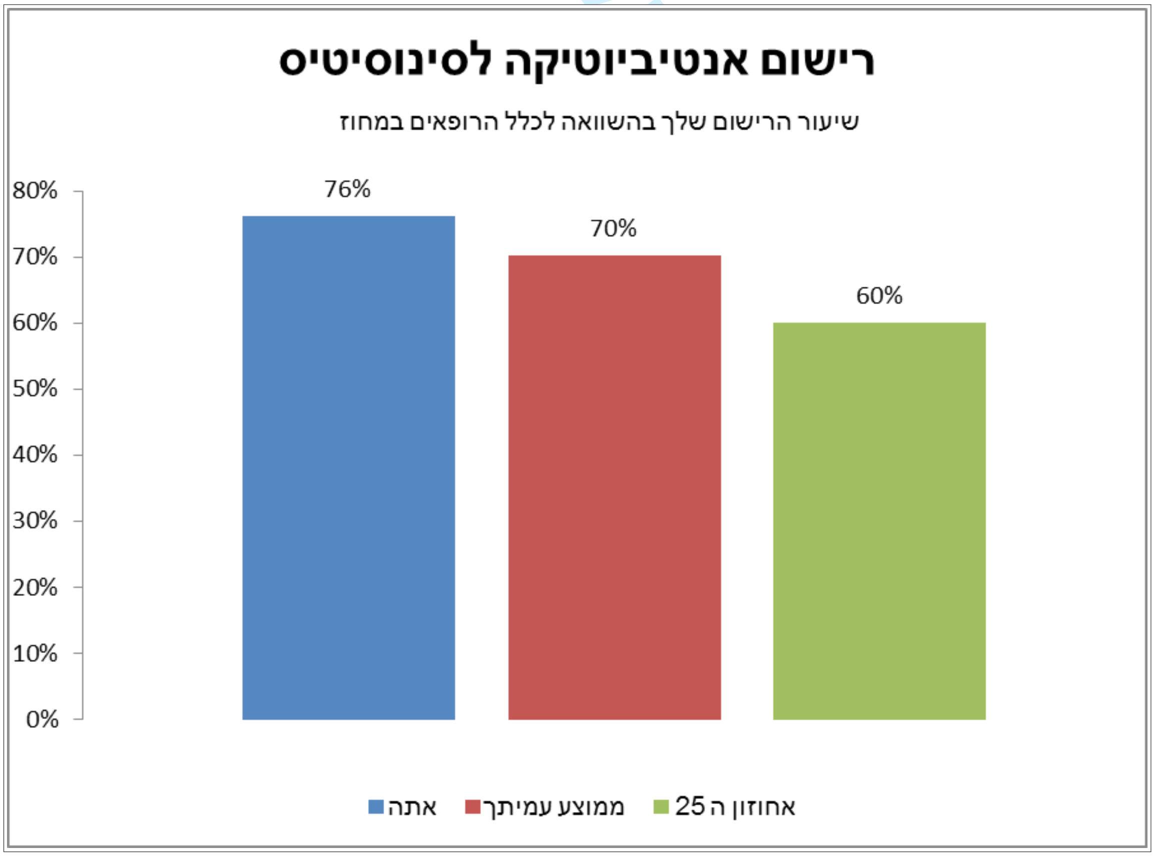

For peer review only - http://bmjopen.bmj.com/site/about/guidelines.xhtml

- מידה רבה
- במידה מסוימת
- במידה מועטה
- בכלל לא
- לא יודע

שאלה 3: האם הצלחת להתמצא בקלות בדו"ח האישי?
- כן
- לא
- לא יודע

שאלה 4: האם יש, לדעתך, תועלת בצירוף קווים מנחים לדו"ח האישי (ראה דוגמה להלן)?

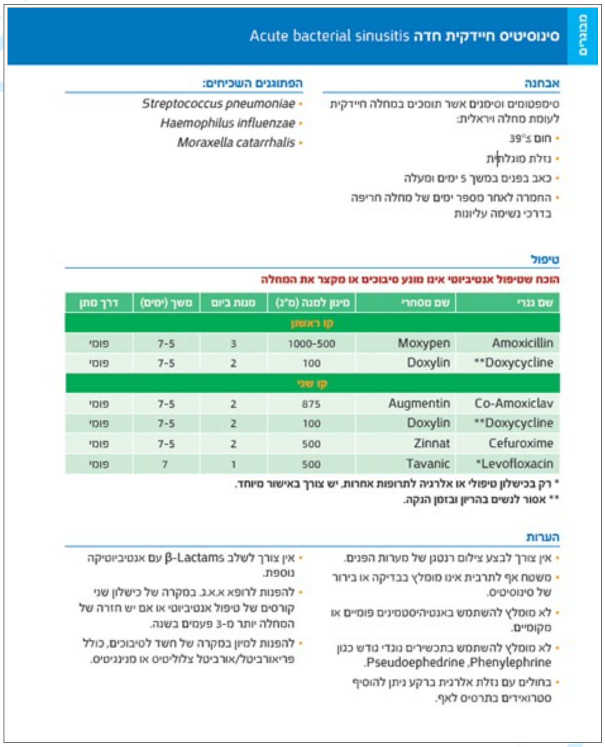
מבוגרים

סינוסיטיס חיידקית חדה Acute bacterial sinusitis

אבחנה

סימפטומים וסימנים אשר תומכים במחלה חיידקית לעומת מחלה ויראלית:
- חום ≥39°
- נזלת מוגלתית
- כאב בפנים במשך 5 ימים ומעלה
- החמרה לאחר מספר ימים של מחלה חריפה בדרכי נשימה עליונות

הפתוגנים השכיחים:
- *Streptococcus pneumoniae*
- *Haemophilus influenzae*
- *Moraxella catarrhalis*

טיפול

הוכח שטיפול אנטיביוטי אינו מונע סיבוכים או מקצר את המחלה

שם גנרי	שם מסחרי	מינון למנה (מ"ג)	מנות ביום	משך (ימים)	דרך מתן
**קו ראשון**					
Amoxicillin	Moxypen	1000-500	3	7-5	פומי
**Doxycycline	Doxylin	100	2	7-5	פומי
**קו שני**					
Co-Amoxiclav	Augmentin	875	2	7-5	פומי
**Doxycycline	Doxylin	100	2	7-5	פומי
Cefuroxime	Zinnat	500	2	7-5	פומי
*Levofloxacin	Tavanic	500	1	7	פומי

*** רק בכישלון טיפולי או אלרגיה לתרופות אחרות, יש צורך באישור מיוחד.**
**** אסור לנשים בהריון ובזמן הנקה.**

הערות
- אין צורך לבצע צילום רנטגן של מערות הפנים.
- משטח אף לתרבית אינו מומלץ בבדיקה או בירור של סינוסיטיס.
- לא מומלץ להשתמש באנטיהיסטמינים פומיים או מקומיים.
- לא מומלץ להשתמש בתכשירים נוגדי גודש כגון Phenylephrine, Pseudoephedrine.
- בחולים עם נזלת אלרגית ברקע ניתן להוסיף סטרואידים בתרסיס לאף.
- אין צורך לשלב β-Lactams עם אנטיביוטיקה נוספת.
- להפנות לרופא א.א.ג. במקרה של כישלון שני קורסים של טיפול אנטיביוטי או אם יש חזרה של המחלה יותר מ-3 פעמים בשנה.
- להפנות למיון במקרה של חשד לסיבוכים, כולל פריאורביטל/אורביטל צלוליטיס או מנינגיטיס.

- כן
- לא
- לא יודע

שאלה 5: אנא הענק ציון לכל אחד מההיגדים הבאים, בהתאם לתועלת הפוטנציאלית עבורך בדו"ח האישי ששלחנו לך. (מועיל מאוד, מועיל במידה מסוימת, מועיל במידה מועטה, כלל לא מועיל, לא יודע)
- אני יכול לראות כיצד דפוס רישום האנטיביוטיקה שלי משתנה לאורך זמן
- אני יכול להשוות את דפוס רישום האנטיביוטיקה שלי לאלה של עמיתיי
- אני יכול לקבל סקירה של דפוסי המרשם המקומיים
- אני יכול להשוות את דפוס הרישום שלי לעומת הקווים המנחים של הכללית
- מאפשר לי לדון עם עמיתיי על מצב דפוסי המרשם המקומיים

שאלה 6: בסך הכל, איזה ציון היית מעניק לתועלת שאתה מפיק מהדו"ח האישי?
- מועיל מאוד

- מועיל במידה מסוימת
- מועיל במידה מועטה
- כלל לא מועיל
- לא יודע

שאלה 7: אנא הצע דרכים לשיפור הדו"ח האישי בנוגע להיבטים הבאים:

1. עריכת התרשימים (כגון צבעים, גודל, מקרא)

[טקסט חופשי]

2. איכות המידע הכלול בדו"ח האישי (כגון מספר התרשימים שכלולים בו, הסברים לתרשימים)

[טקסט חופשי]

3. הערות נוספות

[טקסט חופשי]

Survey 2 (t_3)

שאלה 1: באיזו מידה הפריט הבא – "שיעור מרשמי האנטיביוטיקה שרשמת פר ביקור מטופל בהשוואה לעמיתיך" - סיפק לך את המידע הדרוש לך? (ראה דוגמה להלן)

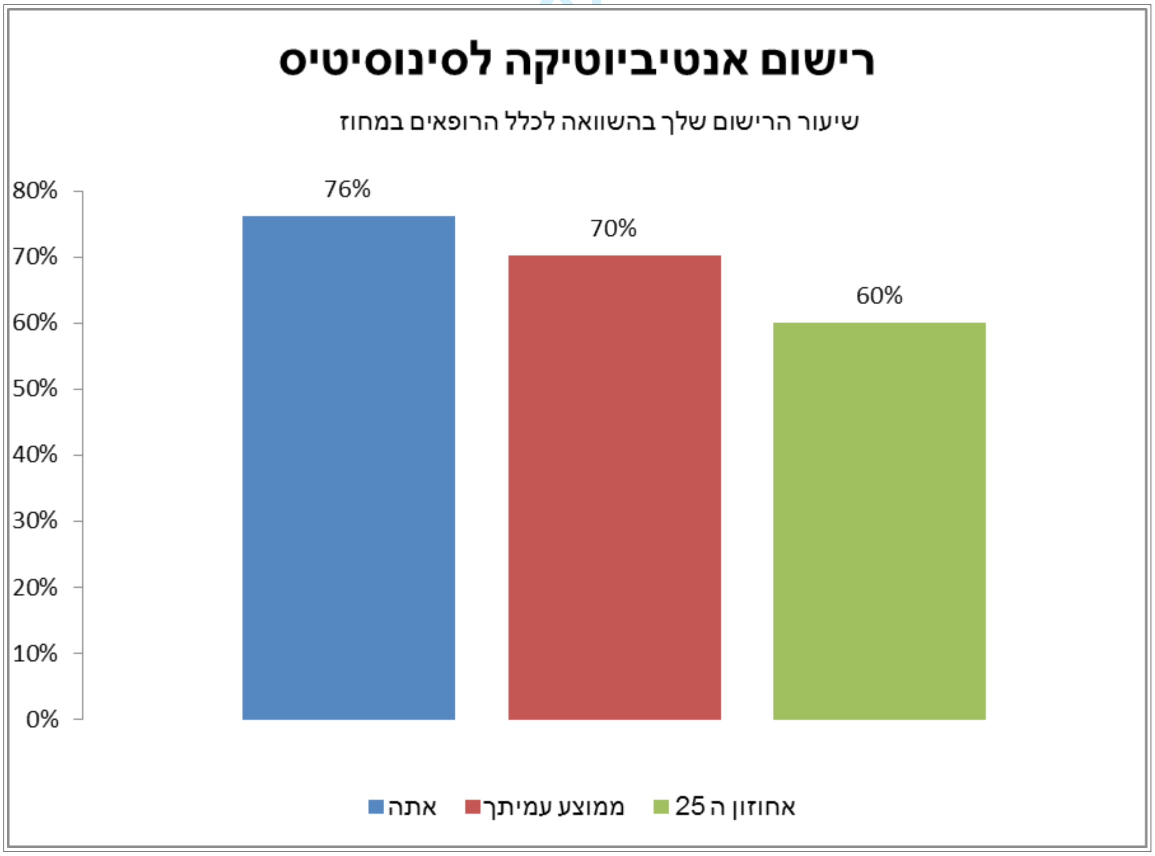

- מידה רבה

- במידה מסוימת
- במידה מועטה
- בכלל לא
- לא יודע

שאלה 2: במהלך שלושת החודשים שעברו, האם הזדמן לך לחשוב על הנתונים שקיבלת כאשר נדרשת לקבל החלטה על רישום אנטיביוטיקה למטופל?

- כן
- לא
- לא יודע

שאלה 3: אנא הענק ציון לכל אחד מההיגדים הבאים, בהתאם לתועלת הפוטנציאלית עבורך בדו"ח האישי ששלחנו לך. (מועיל מאוד, מועיל במידה מסוימת, מועיל במידה מועטה, כלל לא מועיל, לא יודע)

- אני יכול לראות כיצד דפוס רישום האנטיביוטיקה שלי משתנה לאורך זמן
- אני יכול להשוות את דפוס רישום האנטיביוטיקה שלי לאלה של עמיתיי
- אני יכול לקבל סקירה של דפוסי המרשם המקומיים
- אני יכול להשוות את דפוס הרישום שלי לעומת הקווים המנחים של הכללית
- מאפשר לי לדון עם עמיתיי על מצב דפוסי המרשם המקומיים

שאלה 4: בסך הכל, איזה ציון היית מעניק לתועלת שאתה מפיק מהדו"ח האישי?

- מועיל מאוד
- מועיל במידה מסוימת
- מועיל במידה מועטה
- כלל לא מועיל
- לא יודע

שאלה 5: אנא הצע דרכים לשיפור הדו"ח האישי בנוגע להיבטים הבאים:

1. עריכת התרשימים (כגון צבעים, גודל, מקרא)

[טקסט חופשי]

2. איכות המידע הכלול בדו"ח האישי (כגון מספר התרשימים שכלולים בו, הסברים לתרשימים)

[טקסט חופשי]

3. הערות נוספות

[טקסט חופשי]

Survey 3 (t_6)

שאלה 1: באיזו מידה הפריט הבא – "שיעור מרשמי האנטיביוטיקה שרשמת פר ביקור מטופל בהשוואה לעמיתיך" - סיפק לך את המידע הדרוש לך? (ראה דוגמה להלן)

For peer review only

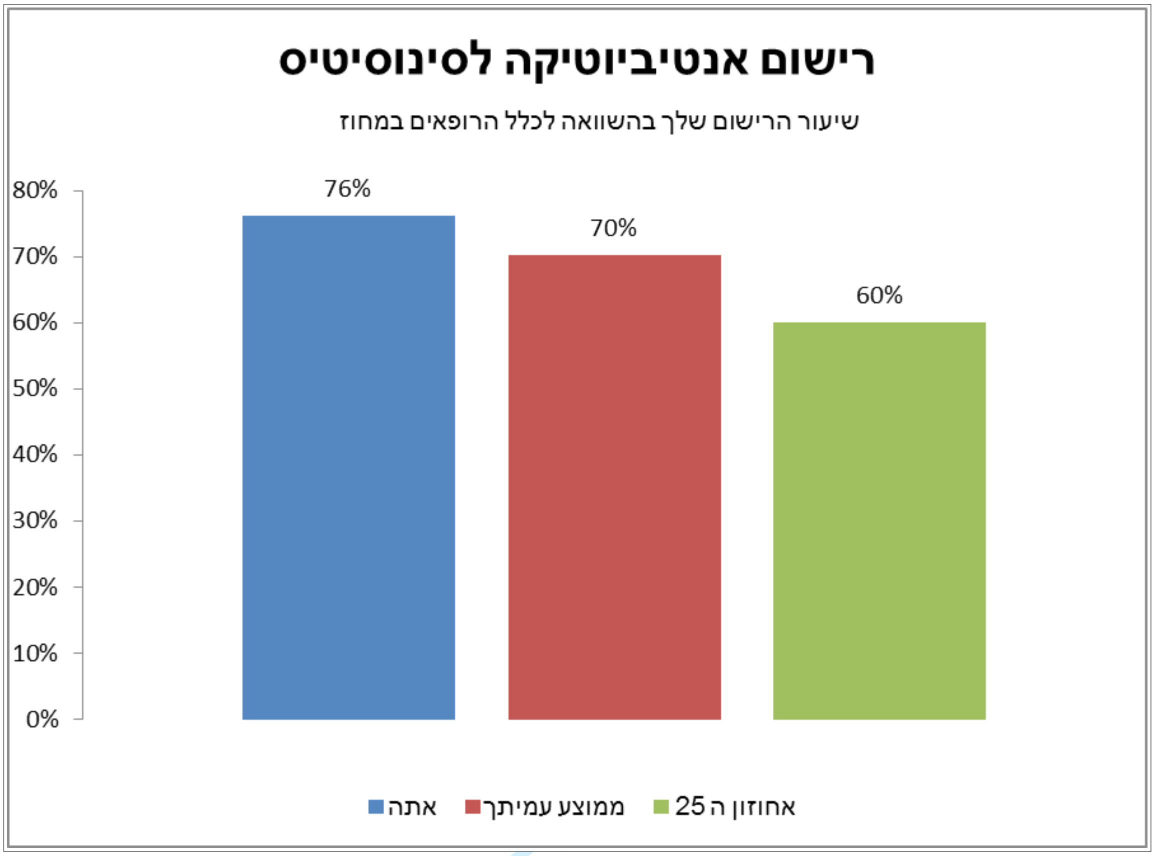

- מידה רבה
- במידה מסוימת
- במידה מועטה
- בכלל לא
- לא יודע

שאלה 2: במהלך שלושת החודשים שעברו, האם הזדמן לך לחשוב על הנתונים שקיבלת כאשר נדרשת לקבל החלטה על רישום אנטיביוטיקה למטופל?

- כן
- לא
- לא יודע

שאלה 3: בסך הכל, איזה ציון היית מעניק לתועלת שאתה מפיק מהדו"ח האישי?

- מועיל מאוד
- מועיל במידה מסוימת
- מועיל במידה מועטה
- כלל לא מועיל
- לא יודע

שאלה 4: באופן כללי, באיזו מידה אתה סבור שהקובץ יועיל לרופאים אחרים?

- יועיל מאוד
- יועיל במידה מסוימת
- יועיל מעט
- לא יועיל בכלל
- לא יודע

שאלה 5: באופן תיאורטי, מהי התדירות הרצויה לדעתך לקבלת הדו"ח האישי?
- אחת לחודש
- אחת לחודשיים
- אחת לרבעון
- אחת לשנה
- בכלל לא

שאלה 6: האם אתה מצליח לעבוד בקלות עם הדו"ח בפורמט PDF?
- כן
- לא
- לא יודע

שאלה 7: האם מאגר מרכזי של קווים מנחים מקומיים עשוי להועיל לך בעבודתך?
- כן
- לא
- לא יודע

שאלה 8: האם אתה עשוי למצוא תועלת בקבלת גישה לפורטל אישי ברשת האינטרנט, בו תוכל למצוא את דפוסי רישום האנטיביוטיקה האישיים שלך וכן את דפוסי רישום האנטיביוטיקה המקומיים, בצירוף הקווים המנחים הרלוונטיים?
- כן
- לא
- לא יודע

שאלה 9: האם תהיה מוכן להשתתף בראיון עוקב (follow-up), בו תישאל על ניסיונך עם הדו"חות האישיים בצורה מפורטת יותר?
- כן
- לא
- לא יודע

שאלה 10 :אנא הצע דרכים לשיפור הדו"ח האישי בנוגע להיבטים הבאים:

1. עריכת התרשימים (כגון צבעים, גודל, מקרא)

[טקסט חופשי]

2. איכות המידע הכלול בדו"ח האישי (כגון מספר התרשימים שכלולים בו, הסברים לתרשימים)

[טקסט חופשי]

3. הערות נוספות

[טקסט חופשי]

For peer review only

Appendix 3. OPEN Stewardship feedback survey pilot protocol and interview guide.

OPEN Stewardship Survey: Pilot testing of questionnaires #1, #2 and #3
***** INTERVIEW GUIDE AND TEMPLATE FOR RESPONSES *****

Purpose of the pilots

- To identify issues (clarity, technical errors etc) in the questionnaires and find potential solutions to improve the questionnaires, key points being:
  - Do the respondents understand the purpose of the questionnaire?
  - Do the respondents find the questions relevant?
  - Do the respondents think that there are questions that are missing?
- To get a realistic estimate of the response time.

Before the interview

- **Identify respondents and book time**
- Identify two respondents from each country and sector (humans/companion animals/dairy) who are willing to pilot test the questionnaire, either among those that have been recruited to the OPEN Stewardship study or other similar persons (or a combination of both), do what is most feasible.
- If possible, identify pilot respondents who are geographically close to you so that you can sit next to the respondent when conducting the pilot interview. An interview over phone, Skype or similar is acceptable but a less preferred option.
- If possible, of the two persons per country and sector, select one person with more years with relevant practice and one with less.
- Each pilot respondent will go through two questionnaires: one which is identical to questionnaire #1 and one which includes only those questions that are added to questionnaire #2 and to questionnaire #3 (referred to as pilot questionnaire #2)
  Estimated time for the pilot session is 60 minutes plus introduction and wrap up time, in total up to 90 minutes, so book 1.5 hour for the interview.

- **Communicate with each respondent before the interview**
- Send a (generic) OPEN Stewardship report one week before the booked interview.
- Ask the respondent to spend about 10 minutes to read it at some point before the booked interview.
- Ask the respondent to make a note of the date they read the report.
- Inform the respondent that during the interview you will go through an online questionnaire on his or her computer/device. You will also email some information during the interview. The respondent must thus have internet access plus access to email.

- **Material the interviewer should bring to the interview**
- Laptop or similar, with:
  - This guide in electronic format
  - Draft emails with link to pilot questionnaires to send during the interview
- A printed version of the introductory text that will be sent with each questionnaire
- Pen and paper
- Watch.

During the interview

For peer review only

Use this template during the interview as a guideline for the information that should be given to the respondent. Record the responses to the interview questions in the boxes as specified.
Please fill out and save one template per respondent.
Name each file: COUNTRY_SECTOR_ABBREVIATED-NAME-OF-RESPONDENT (first two letters of given name and first two letters of family name).
You may of course use your own words whenever you wish.

Name of interviewer
Fill out here

Abbreviated name of respondent (first two letters of given name and family name respectively)
Fill out here

- **INTRODUCTION TO INTERVIEW**

Thank you for taking the time to do this interview. As I mentioned previously, I estimate that it will take about 60 minutes.
The aim of the OPEN Stewardship project is to improve appropriate antibiotic prescribing by generating customised reports of individual prescribing in relation to peers. We sent you one of these reports a week ago.
Surveys will be sent out to practitioners enrolled in the OPEN Stewardship intervention at three occasions, to improve the reports.
We are now doing interviews to find out what respondents think about the survey questions. This is to ensure that the surveys measure the right things, so that we will get as good results as possible in order to improve our system. The surveys will be sent out by email and will be filled out online.
Just so you know, I am not the one who have made the survey questions we are about to go through. All of your comments are greatly appreciated.
Approximately 10 people will be interviewed about the survey questions in this pilot. This is an international collaboration, and we work together with colleagues in Canada, Israel, Sweden and the USA [REMOVE YOUR OWN COUNTRY].
If you need a break at any time during the interview just let me know.
Any questions before we get started?

For peer review only

- **SAMPLE REPORT INFORMATION**

Have you had time to read the OPEN Stewardship report?
Interviewer fill out here

- *If not, allow for up to 10 minutes to look at it (but don't rush the person).*

Roughly, when did you read the report? *Or note if the respondent is reading it right now*
Interviewer fill out here

- **EMAIL WITH QUESTIONNAIRE INFORMATION**

So we will start by looking at the text that will accompany the questionnaire, which will be sent out through a link in an email. If you can please read through this email.

- Hand over the paper with the email text. *If the interview is done over the phone, email the text to the respondent now.*

Do you have any comments?
Interviewer fill out here
Is there anything you feel is unclear?
Interviewer fill out here

Do you think the text would motivate someone to answer the survey?
Interviewer fill out here
Any suggestions?
Interviewer fill out here

Thank you!

- **QUESTIONNAIRE 1**

We will now move on to the first questionnaire, and I will email you a link to this questionnaire.

- Email the link to pilot questionnaire 1.

I would like to ask you to fill out the whole questionnaire, but don't press "submit" at the end. Take your time. If you wish you can make notes if there is anything that you come to think of; we will later go through the questionnaire question by question.

For peer review only

- Give the respondent a piece of paper and a pen.
- Respondent fills out the questionnaire.
- Note how long it takes in minutes to complete questionnaire.

Interviewer fill out here

- Note if the respondent seems to spend a longer time reading/re-reading any of the questions.

Interviewer fill out here

Thanks!
So let us start from the beginning with the introduction to the survey.

Do you think this text is informative enough? Any suggestions?
Interviewer fill out here

We will now go through each question, one at a time. I am interested in your opinion, in this situation there is nothing that is right or wrong. So we start with the first question.

- In your discussion with the respondent, note also if anything he or she says that is unexpected/odd.

- **"Is the purpose of the OPEN Stewardship report clear?"**

Can you please describe why you gave this response?
Interviewer fill out here

- **"How informative did you find the following item in the OPEN Stewardship report: Your Prescribing Rate per Visit Compared to Peers"**

Can you please describe why you gave this response?
Interviewer fill out here

Is it clear to you what the various responses mean?
Interviewer fill out here

- **"Was it straightforward how to navigate through the OPEN Stewardship report?"**

Can you please describe why you gave this response?
Interviewer fill out here

For peer review only

- **"Is it useful to include Best Practice Guidelines in the OPEN Stewardship report"**

Can you please describe why you gave this response?
Interviewer fill out here

- **"Rate each of the following items according to its potential usefulness to you in the OPEN Stewardship report."**

- **"See how my prescribing changes over time"**

Can you please describe why you gave this response?
Interviewer fill out here

- **"Compare my own prescribing with peers"**

Can you please describe why you gave this response?
Interviewer fill out here

- **"Get an overview of local prescribing patterns"**

Can you please describe why you gave this response?
Interviewer fill out here

- **"Compare my own prescribing with guidelines"**

Can you please describe why you gave this response?
Interviewer fill out here

- **"Have discussions with my peers on the local situation … "**

Can you please describe why you gave this response?
Interviewer fill out here

Is it clear to you what the various responses mean?
Interviewer fill out here

- **"Overall, how would you rate the usefulness of the OPEN Stewardship report?"**

Can you please describe why you gave this response?
Interviewer fill out here

- **“Please suggest how to improve the OPEN Stewardship report:”**
- **“Layout of the graphs (such as colors, size, legends).”**

Is it clear to you what kind of responses could be given here?
Interviewer fill out here

- **“Information included (such as number of graphs, explanatory text).”**

Is it clear to you what kind of responses could be given here?
Interviewer fill out here

- **“Other comments.”**

Is it clear to you what kind of responses could be given here?
Interviewer fill out here

I would now like to continue with the layout of the questionnaire.

- Note any potential changes that are indicated by the responses.

What was the most difficult part to navigate through?
Interviewer fill out here

For which questions did you have to use the scroll bar?
Interviewer fill out here

For peer review only

If you now think about the questionnaire as a whole,

What is your overall impression?
Interviewer fill out here

After reading these questions – what would you say is the purpose of this survey?
Interviewer fill out here

Are there any questions that are *not* relevant?
Interviewer fill out here

Are there any questions that should be phrased differently?
Interviewer fill out here

Is any type of question missing?
Interviewer fill out here

What about the length of the survey?
Interviewer fill out here

- **QUESTIONNAIRE 2**

The second and last questionnaire that I want you to go through contains some other questions that we want to ask when evaluating the project and the reports on prescription patterns. It contains 7 questions. I will now email you the link to this questionnaire.

- Email the link to pilot questionnaire 2.

I would like to ask you to now fill out the second questionnaire.

- Respondent fills out the questionnaire.

Again we will now go through each question one at a time.

- **"1. In the past three months, did you ever think about your previous OPEN Stewardship report when making an antibiotic prescribing decision?"**

Can you please describe why you gave this response?
Interviewer fill out here

- **"2. In general, how useful do you think the OPEN Stewardship report would be to other prescribers?"**

Can you please describe why you gave this response?
Interviewer fill out here

Is it clear to you what the various responses mean?
Interviewer fill out here

- **"3. Hypothetically, how often do you think it would be appropriate to receive OPEN Stewardship reports?"**

Can you please describe why you gave this response?
Interviewer fill out here

Is it clear to you what the various responses mean?
Interviewer fill out here

- **"4. Does the pdf format of the OPEN Stewardship report work well for you?"**

Can you please describe why you gave this response?
Interviewer fill out here

- **"5. Would a central repository of local treatment guidelines be useful for your practice?"**

What does this question mean, can you describe it in other words?
Interviewer fill out here

- **"6. Do you think it would be useful to have access to a personalized web-portal where you could browse your own and local patterns of antibiotic use, as well as best practice guidelines?"**

What does this question mean, can you describe it in other words?
Interviewer fill out here

For peer review only

- **“7. Would you be willing to participate in a follow-up interview where we would ask in more detail about your experiences with the OPEN Stewardship reports?”**

Can you please describe why you gave this response?
Interviewer fill out here

- **END OF INTERVIEW**

- Note which kind of device and browser the respondent uses.

Interviewer fill out here

We have now finished. On behalf of me and all my colleagues in the project, I would like to thank you so much for taking the time to help us to improve the evaluation. It has been most useful for our work. If there is anything that you come to think of afterwards, do not hesitate to contact me.

After the interview

Email the filled out templates (in English in case you have used another language for your notes) to the Public Health Agency of Sweden who will compile from all sites. Make sure that the file names are: COUNTRY_SECTOR_ABBREVIATED-NAME-OF-RESPONDENT.

For peer review only

BMJ Open

Evaluation of an automated feedback intervention to improve antimicrobial prescribing among primary care physicians (OPEN Stewardship): Protocol for an interrupted time-series and usability analysis in Ontario, Canada and Southern Israel

Journal:	*BMJ Open*
Manuscript ID	bmjopen-2020-039810.R1
Article Type:	Protocol
Date Submitted by the Author:	05-Sep-2020
Complete List of Authors:	Soucy, Jean-Paul; University of Toronto Dalla Lana School of Public Health, Division of Epidemiology<br>Low, Marcelo; Clalit Health Services<br>Acharya, Kamal; University of Guelph Ontario Veterinary College, Department of Population Medicine<br>Ellen, Moriah; University of the Negev Guilford Glazer Faculty of Business and Management, Department of Health Services Management<br>Hulth, Anette; Folkhalsomyndigheten<br>Löfmark, Sonja; Folkhalsomyndigheten<br>Garber, Gary; Public Health Ontario, Infection Prevention and Control; University of Ottawa Faculty of Medicine, Medicine/infectious diseases<br>Watson, William; University of Toronto; St Michael's Hospital<br>Moran-Gilad, Jacob ; Ben-Gurion University of the Negev Faculty of Health Sciences<br>Fisman, David N.; University of Toronto Dalla Lana School of Public Health, Division of Epidemiology<br>MacFadden, Derek; Ottawa Hospital Research Institute, Infectious Diseases
**Primary Subject Heading**:	Public health
Secondary Subject Heading:	General practice / Family practice, Infectious diseases
Keywords:	INFECTIOUS DISEASES, Public health < INFECTIOUS DISEASES, Epidemiology < INFECTIOUS DISEASES, PRIMARY CARE

SCHOLARONE™
Manuscripts

For peer review only - http://bmjopen.bmj.com/site/about/guidelines.xhtml

I, the Submitting Author has the right to grant and does grant on behalf of all authors of the Work (as defined in the below author licence), an exclusive licence and/or a non-exclusive licence for contributions from authors who are: i) UK Crown employees; ii) where BMJ has agreed a CC-BY licence shall apply, and/or iii) in accordance with the terms applicable for US Federal Government officers or employees acting as part of their official duties; on a worldwide, perpetual, irrevocable, royalty-free basis to BMJ Publishing Group Ltd ("BMJ") its licensees and where the relevant Journal is co-owned by BMJ to the co-owners of the Journal, to publish the Work in this journal and any other BMJ products and to exploit all rights, as set out in our licence.

The Submitting Author accepts and understands that any supply made under these terms is made by BMJ to the Submitting Author unless you are acting as an employee on behalf of your employer or a postgraduate student of an affiliated institution which is paying any applicable article publishing charge ("APC") for Open Access articles. Where the Submitting Author wishes to make the Work available on an Open Access basis (and intends to pay the relevant APC), the terms of reuse of such Open Access shall be governed by a Creative Commons licence – details of these licences and which Creative Commons licence will apply to this Work are set out in our licence referred to above.

Other than as permitted in any relevant BMJ Author's Self Archiving Policies, I confirm this Work has not been accepted for publication elsewhere, is not being considered for publication elsewhere and does not duplicate material already published. I confirm all authors consent to publication of this Work and authorise the granting of this licence.

For peer review only

Evaluation of an automated feedback intervention to improve antimicrobial prescribing among primary care physicians (OPEN Stewardship): Protocol for an interrupted time-series and usability analysis in Ontario, Canada and Southern Israel

Jean-Paul R. Soucy[1], Marcelo Low[2, 3], Kamal R. Acharya[4], Moriah Ellen[5, 6, 7], Anette Hulth[8,], Sonja Löfmark[8], Gary E. Garber[9, 10, 11], William Watson[12, 13], Jacob Moran-Gilad[14], David N. Fisman[1]*, Derek R. MacFadden[15]*

* co-senior authors

[1] Division of Epidemiology, Dalla Lana School of Public Health, University of Toronto, Toronto, Canada
[2] Clalit Health Services, Chief Physician's Office, Tel Aviv, Israel
[3] School of Public Health, University of Haifa, Haifa, Israel
[4] Department of Population Medicine, University of Guelph, Guelph, Canada
[5] Department of Health Systems Management, Guilford Glazer Faculty of Business and Management and Faculty of Health Sciences, Ben-Gurion University of the Negev, Beer Sheva, Israel
[6] McMaster Health Forum, McMaster University, Hamilton, Canada
[7] Institute for Health Policy, Management and Evaluation, University of Toronto, Toronto, Canada
[8] Public Health Agency of Sweden, Stockholm, Sweden
[9] Infection Prevention and Control, Public Health Ontario, Toronto, Canada
[10] Department of Medicine, University of Toronto, Toronto, Canada
[11] Department of Medicine, University of Ottawa, Ottawa, Canada
[12] St Michael's Hospital, Toronto, Canada
[13] Department of Family & Community Medicine, University of Toronto, Canada
[14] School of Public Health, Faculty of Health Sciences, Ben-Gurion University of the Negev, Beer Sheva, Israel
[15] Ottawa Hospital Research Institute, Ottawa, Canada

Corresponding author: Derek R. MacFadden (dmacfadden@toh.ca): Ottawa Hospital Research Institute, 1053 Carling Ave, Ottawa, ON, Canada K1Y 4E9

Word count: 3,250

Keywords: antimicrobial stewardship, antimicrobial resistance, antimicrobial prescribing, primary care, interrupted time-series analysis

For peer review only

ABSTRACT

Introduction

Antimicrobial resistance undermines our ability to treat bacterial infections, leading to longer hospital stays, increased morbidity and mortality, and a mounting burden to the healthcare system. Antimicrobial stewardship is increasingly important to safeguard the efficacy of existing drugs, as few new drugs are in the developmental pipeline. While significant progress has been made with respect to stewardship in hospitals, relatively little progress has been made in the primary care setting, where the majority of antimicrobials are prescribed. OPEN Stewardship is an international collaboration to develop an automated feedback platform to improve responsible antimicrobial prescribing among primary care physicians and capable of being deployed across heterogeneous healthcare settings. We describe the protocol for an evaluation of this automated feedback intervention with two main objectives: assessing changes in antimicrobial prescribing among participating physicians and determining the usability and usefulness of the reports.

Methods and analysis

A non-randomized evaluation of the automated feedback intervention (OPEN Stewardship) will be conducted among approximately 150 primary care physicians recruited from Ontario, Canada and Southern Israel, based on a series of targeted stewardship messages sent using the platform. Using a controlled interrupted time-series analysis and multilevel negative binomial modelling, we will compare the antimicrobial prescribing rates of participants before and after the intervention, and also to the prescribing rates of non-participants (from the same healthcare network) during the same period. We will examine outcomes targeted by the stewardship messages, including prescribing for antimicrobials with duration longer than seven days and prescribing for indications where antimicrobials are typically unnecessary. Participants will also complete a series of surveys to determine the usability and usefulness of the stewardship reports.

Ethics and dissemination

All sites have obtained ethics committee approval to recruit providers and access anonymized prescribing data. Dissemination will occur through open-access publication, stakeholder networks, and national/international meetings.

STRENGTH AND LIMITATIONS OF THIS STUDY

- Multinational pragmatic study
- Large number of providers studied with extended follow-up
- Comprehensive evaluation including pre-post design and concurrent controls
- Scalable and open concept with potential for broad generalizability to various care settings
- Differences in recruitment strategy for intervention group across the two study sites may result in heterogeneity of intervention effect

For peer review only

INTRODUCTION

The World Health Organization has called antimicrobial resistance "one of the biggest threats to global health, food security, and development today." [1] Antimicrobial resistance undermines our ability to treat bacterial infections, leading to longer hospital stays, increased complications, higher mortality, and a mounting resource burden on healthcare systems globally [1–5]. It has been projected that resistant microbes could contribute to up to 10 million deaths per year by 2050, greater than the current annual burden of cancer deaths. Additionally, a cumulative loss in global production of trillions of USD in the same time frame is possible [6], hindering efforts to eliminate poverty [7]. Despite a widespread recognition of antimicrobial use as the leading driver of resistance, inappropriate prescribing remains common [8–10]. Given the paucity of new antimicrobials in the drug development pipeline [11], it is critical that we act to safeguard the effectiveness of existing drugs by reducing inappropriate antimicrobial prescribing.

Antimicrobial stewardship refers to the effort to promote the responsible use of antimicrobials and to ensure that patients receive the best choice, duration, and route of antimicrobial treatment, while minimizing potential side effects and the dissemination of antimicrobial resistance [12]. While progress has been made in advancing antimicrobial stewardship in hospitals, leading to significant cost savings and improved patient outcomes [13,14], progress in the primary care setting has been slower [15–17]. This is troubling, given that most antimicrobials are prescribed in the outpatient setting, rather than in hospitals (e.g., over 90% in Canada) [18,19]. Supporting general and family practitioners with prescribing is especially important, since they account for the majority of antimicrobials dispensed for human use in Canada [19]. The United States Centers for Disease Control and Prevention have issued a checklist identifying the key elements to a successful antimicrobial stewardship program in an outpatient setting, highlighting easy access to evidence-based prescribing guidelines and the technical capacity to track, evaluate, and report on antimicrobial prescribing [20]. An online, automated feedback system facilitating these elements would be a major asset to advancing stewardship in primary care.

OPEN Stewardship is an international collaboration with the goal of developing an open, web-based platform to improve antimicrobial prescribing among primary care physicians. This platform integrates with local antimicrobial prescribing data to provide participating physicians with regular, automated feedback (identified by a recent Cochrane review as an effective technique to enable appropriate antimicrobial prescribing in the hospital setting [14]) and connects users with locally relevant prescribing guidelines. The development of a standardized, accessible stewardship platform addresses a key gap for stewardship interventions in the primary care setting, namely the difficulty of expanding across heterogeneous, resource-constrained healthcare systems. As part of a One Health approach recognizing the interconnectedness of human and animal health [21], the platform is also being developed for use in veterinary care, a sector where antimicrobial stewardship has received less attention [22]. A study parallel to the present study will be occurring among veterinary care providers.

STUDY APPROACH AND OBJECTIVES

In this study, we will enrol primary care physicians on a voluntary basis from practices in two study sites: Ontario, Canada and Southern Israel. Non-participating physicians will be available as controls. The intervention group will receive three personalized electronic reports sent over the span of six months, benchmarking their prescribing rate to those of their peers and attaching locally relevant prescribing guidelines for specific indications. The messaging will target antimicrobial prescribing longer than seven days (few conditions require longer than this [23]), as well as two indications in particular: acute sinusitis and viral respiratory conditions. These common infections are a promising

For peer review only

target for stewardship [8,20,24,25]. In the US, acute respiratory conditions as a whole account for close to half of antimicrobials prescribed and a great majority of those prescribed inappropriately in the ambulatory care setting [8].

The aim of this project is to assess the impact of an automated feedback intervention on antimicrobial prescribing in primary care physicians. Specifically, for a cohort of primary care physicians in Ontario, Canada and Southern Israel, we will determine the impact of the stewardship intervention on:

1. The overall antimicrobial prescribing rate in the period following the intervention compared to the period preceding it and to a cohort of primary care physicians who did not receive the intervention.

2. The prescribing rate for antimicrobials with duration longer than seven days in the period following the intervention compared to the period preceding it and to a cohort of primary care physicians who did not receive the intervention.

3. The antimicrobial prescribing rate for viral respiratory conditions in the period following the intervention compared to the period preceding it and to a cohort of primary care physicians who did not receive the intervention.

4. The antimicrobial prescribing rate for acute sinusitis in the period following the intervention compared to the period preceding it and to a cohort of primary care physicians who did not receive the intervention.

We will also assess:

5. The usability and usefulness of each component of the antimicrobial stewardship reports, as well as the usefulness of the reports overall.

METHODS AND ANALYSIS

Objectives 1–4: Non-randomized evaluation of intervention with interrupted time-series analysis

Study design

Primary care physicians (providers) from two study sites will be voluntarily enrolled to participate in an antimicrobial stewardship intervention. The providers' rates of antimicrobial prescribing after the intervention will be compared to their rates prior to having received the reports, as well as the prescribing rates of their peers in the same network who did not participate in the intervention. This study design is know as a controlled interrupted time-series [26]. Our study design addresses the limitations of some previous studies of stewardship interventions, including small sample sizes, single-centre design, a lack of external controls, and short follow-up [16].

Participants

Approximately 100–150 primary care physicians will be enrolled from two study sites: Ontario, Canada (50) and Southern Israel (50–100). Participants will be drawn from the University of Toronto Practice-Based Research Network (UTOPIAN) and Clalit Health Services (CHS), respectively. UTOPIAN is "one of the largest and most representative primary care research networks in North America" [27], including data for over 400 physicians with an average practice size of 1,018 patients

For peer review only

[28]. CHS is the largest healthcare maintenance organization in Southern Israel [29], and covers nearly 80% of the population in Southern Israel. Existing data storage and sharing facilities within both of these networks allow access to de-identified patient data for research in the primary care setting.

In Ontario, primary care physicians will be voluntarily enrolled, provided they worked for at least 12 months prior the intervention, expect to continue working for the 12 months following the start of the intervention, and are not actively participating in another stewardship intervention. Providers will be approached for enrolment via email or in-person presentations to groups of providers. Participants will begin the intervention in at least two waves in different months. In Southern Israel, half of the approximately 400 eligible physicians (average number of patients: 1,482) will be randomized (using random assignment in an Excel spreadsheet by the study coordinator) to be approached (via a letter from the Chief Physician, followed by contact by an interviewer) for voluntary enrolment in the study. Anonymized prescribing data of non-participating physicians within UTOPIAN and CHS will be available as an external control group (approximately 350 for UTOPIAN and 250–300 for CHS, with an expected 25–50% response rate for physicians approached to enrol).

It is possible that physicians who voluntarily enrol in the antimicrobial stewardship intervention will be more engaged with stewardship prior to the intervention, and thus may have less to gain from a stewardship intervention than less-engaged physicians. Differences in the recruitment strategy between sites may result in a heterogeneity in the observed effect, which will be accounted for in the analysis.

Intervention

The intervention will consist of three personalized reports generated by the OPEN Stewardship platform and emailed to participants in the intervention group. The first report will be sent at the beginning of the intervention period (t_0) and will consist of a welcome email followed by a benchmark of the provider's overall antimicrobial prescribing rate relative to the average and 25th percentile of participants in the same network (UTOPIAN or CHS) during the previous year. This report will also include guidelines highlighting that most conditions do not require more than 7 days of antimicrobials and a benchmark of the provider's proportion of antimicrobial prescriptions with duration longer than 7 days relative to the average and 25th percentile of participants in the same network. The two subsequent reports will be sent 3 (t_3) and 6 (t_6) months after the first report. These reports will target viral respiratory conditions (as defined by the ICD-9 code associated with the visit, see table S1) and acute sinusitis, respectively, and will consist of a benchmark of the provider's prescribing for the specified indication as well as site-specific best practice guidelines for the specified indication. The study concludes one year after the first report is sent (t_{12}). The composition of each report is summarized in table 1. An example figure is shown in figure 1.

Outcomes

Each of the 4 objectives corresponds to an outcome:

1. Each provider's overall monthly prescribing rate for antimicrobials, as measured by the number of prescriptions divided by the number of patients visits in that month.
2. The prescribing rate for antimicrobials lasting longer than seven days in duration.
3. The prescribing rate for visits classified as being for a viral respiratory condition (see table S1 for ICD-9 codes).
4. The prescribing rate for acute sinusitis visits.

For peer review only - http://bmjopen.bmj.com/site/about/guidelines.xhtml

While overall antimicrobial prescribing is a proxy for our desired measure, which is appropriate antimicrobial prescribing, the microbiological data do not exist to validate the appropriateness of each individual prescription. Nonetheless, given the ubiquity of inappropriate prescribing, particularly for acute respiratory tract infections, any reduction in prescribing as a result of the intervention is likely to be positive [8,30,31].

Covariates

The analysis will include potential predictors of a provider's baseline prescribing rate, as well as seasonality (table 2). These include a provider's age, sex, and employment status (full-time or part-time) [10] and monthly practice patient composition (by age and sex) [19,32]. We will also use nested random intercepts for provider, practice group (clinic), and study site, representing within-group prescribing norms and a provider's baseline willingness to prescribe after accounting for other predictors, as well as heterogeneity between study sites. Seasonality will be accounted for using a categorical variable for each month compared to January. An interaction term with study site will be included for each covariate to allow for differences in seasonal and demographic prescribing patterns between the two countries in our study.

Statistical analysis

We will perform a multilevel, controlled interrupted time-series analysis with the provider as the unit of analysis [33]. A negative binomial model (to allow for overdispersion of the outcome) will be used with prescribing rates as the outcome. Each provider in the intervention group will contribute 24 observations: a minimum of 12 months in the pre-intervention period (t_{-12} to t_0) and up to 12 months in the post-intervention period, depending on the objective. The pre-intervention and post-intervention periods of each outcome are defined by the time when the relevant report was delivered. The first report (t_0) corresponds to objectives 1 and 2, the second report (t_3) to objective 3, and the third report (t_6) to objective 4. The pre-intervention and post-intervention periods of each objective are summarized in table 3. Providers in the external control group will contribute 24 observations in the control period. The timeline of the study for each objective is shown in figure 2.

The intervention effect will be modelled as a level change and/or change in slope for the prescribing rate at the beginning of the post-intervention period [26]. An interaction term with study site will be included to allow for differences in the intervention effect between Ontario and Southern Israel. A key assumption of interrupted time-series analysis is that the pre-intervention time trend is assumed to be linear, which must be evaluated through visualization [34]. Otherwise, non-linear terms may be considered. Model fit can be assessed by comparing models containing intervention parameters to a null model not containing intervention parameters using a statistic such as Akaike information criterion (AIC).

Autocorrelation in each provider's time-series should be accounted for by the random intercept (provider nested within practice group nested within study site) and by controlling for seasonality (categorical variable for month) [26]. In case autocorrelation is present in the residual distribution, an autoregressive (AR1) covariance structure will be considered.

Stewardship interventions often result in a reversion to baseline behaviour after the intervention is discontinued [35,36]. To examine the durability of the intervention effect, we will contrast the coefficients estimated from models including 1) all months in the post-intervention period, 2) the first 3 months in the post-intervention period, 3) the first 6 months in the post-intervention period. Finally,

For peer review only - http://bmjopen.bmj.com/site/about/guidelines.xhtml

since the first report at t_0 (highlighting overall prescribing) is likely to affect objectives targeted by later reports (at t_3 or t_6), for objectives 3 and 4, we will consider t_0 as an alternative start time of the post-intervention period.

As the COVID-19 pandemic represents a major disruption of primary care, its effect on prescribing will be modelled as one or more separate "intervention points" in the time-series analysis.

Sensitivity analyses

Alternative parameterizations of the study outcomes will be considered. For the prescribing rate of antimicrobials with courses longer than seven days (objective 2), we will consider total days of therapy (with the number of prescriptions as the offset) as an alternative outcome using a zero-truncated negative binomial model. There may be issues with how indications are coded in the electronic medical record, whereas the type of antimicrobial prescribed is very likely to be accurate. A sensitivity analysis will be performed using the prescribing rates (for all visits) for antimicrobials most often prescribed for viral respiratory conditions (e.g., amoxicillin, amoxicillin/clavulanic acid, azithromycin [37]) as the outcome, rather than the indication-specific prescribing rate of antimicrobials for viral respiratory conditions. Since acute respiratory tract infections make up a large proportion of total antimicrobial prescribing, the signal from the intervention may be evident using these outcomes as well.

To ascertain whether there could be negative effects of the stewardship intervention, we will evaluate (1) the frequency of bacterial pneumonia and pyelonephritis diagnoses and (2) the number of hospital admissions (including those related to infection) in the pre-intervention and post-intervention periods. Admissions data are only available for CHS (Israel), and thus will only be evaluated at this site only.

Including patient composition as predictors (e.g., percentage of patients 65 and older, percentage of female patients) may be insufficient to capture differences in prescribing occurring between strata of age and sex. A sensitivity analysis will be performed where the outcome will be stratified by age group and sex and modelled separately. Although stratification increases the noise in each individual time-series, these models may reveal differences in the effectiveness of the intervention across sex/age groups, which have different prescribing patterns.

As the offer to enrol in the study is randomized at one study site (Southern Israel), an intention-to-treat analysis will be performed as a supplementary analysis at this site only. The characteristics and prescribing patterns of responders and non-responders among those offered enrolment will also be compared.

Sample size

We provide an approximate sample size calculation for a difference in antimicrobial prescribing between two groups (intervention and control) in a Poisson framework using the method described by Whitehead [38] and implemented in an online calculator (http://www.obg.cuhk.edu.hk/ResearchSupport/StatTools/SSiz2Counts_Tab.php). In Fleming-Dutra's [8] study of outpatient prescribing, 12.6% of visits resulted in an antimicrobial prescription. Since most visits would only results in a single antimicrobial prescription, we take this as the baseline prescribing rate (0.126/visit). The American Academy of Family Physicians estimated in 2012 that their members had an average of 92 patient encounters per week (368 per month) [39]. We define a 15% reduction in prescribing rate as clinically meaningful (0.107/visit). To achieve a power of 90% (with $\alpha = 0.05$, one-tail), we would require approximately 15 physicians in each group to be observed for 1 month each,

For peer review only

which is much smaller than our expected sample size at each study site. While the clustering and likely over-dispersion of prescribing rates in our sample would decrease our power, the fact that we will have 24 months of observations for each provider (rather than 1 month), as well as pre-intervention and post-intervention observations, greatly enhances the power of our study to detect an intervention effect.

Objective 5: Survey of usability among participating providers

Study design

Physicians enrolled in the antimicrobial stewardship intervention will receive three short surveys to assess the usability of the reports received and to provide feedback on the intervention.

Surveys

Surveys will be delivered by email to participants in the intervention group 3 days after the initial receipt of each report using Qualtrics (Qualtrics, Provo, UT) in Southern Israel and RedCAP [40] survey software in Ontario (due to differences in institutional policies regarding data collection). The report will be re-sent with the survey and the recipient will be given 7 days to respond. If the recipient fails to respond within this allotted time, the survey and report are re-sent again, giving the recipient an additional 7 days to respond. The first two surveys focus on the usability and usefulness of the emailed reports, with additional space for free text feedback. The final report adds additional questions to assess how often they would prefer to receive reports and whether particular additional features would be of use to him or her. Survey questions are given in English in Appendix 1; translated questions in Hebrew are given in Appendix 2. Questions were developed in accordance with experts at the Public Health Agency of Sweden. Questions were pilot tested and revised according to the protocol and interviewer guide in Appendix 3.

Statistical analysis

Participants' attitudes regarding the overall usefulness of the intervention and the informativeness of the figure included in each intervention (table 1) will be assessed in each survey and compared longitudinally with a multilevel linear model. Responses to other questions may be analyzed using ordinal regression and/or multinomial regression.

ETHICS AND DISSEMINATION

The following ethics committees reviewed and approved this study: University of Toronto REB (Canada), St. Michael's Hospital REB (Canada), Toronto East Health Network REB (Canada), Clalit REB (Israel). The veterinary study was further reviewed and approved by: University of Guelph REB (Canada) and Internal Research Review Committee of the Koret School of Veterinary Medicine – Veterinary Teaching Hospital, Hebrew University (Israel).

All patient-level prescribing data will be anonymized prior to receipt, available only as the rate of antimicrobial prescribing per visit, stratified by month and reason for visit (and potentially sex and/or broad age category). Study data will be stored on a secure server and accessible only to study analysts. Study participants from Ontario will be compensated with a gift card. Study participants from Israel will be encouraged to participate by a letter from the Chief Physician's Office.

For peer review only

The OPEN Stewardship project includes collaborators affiliated with hospitals, universities, and public health authorities in four countries. In addition to dissemination through stakeholder networks, the results of this study will be published in an open-access journal and presented at national and/or international meetings.

PATIENT AND PUBLIC INVOLVEMENT

Primary care physicians were involved in the conception and design of this study, as well as the study surveys. They were also consulted to assess the appropriate time burden and adequate compensation for participants in the study.

For peer review only

REFERENCES

1 World Health Organization. Antibiotic resistance. 2019.http://www.who.int/mediacentre/factsheets/antibiotic-resistance/en/ (accessed 1 Jan 2019).

2 The Review on Antimicrobial Resistance. Tackling Drug-resistant Infections Globally: Final Report and Recommendations. 2016.https://amr-review.org/sites/default/files/160518_Final%20paper_with%20cover.pdf (accessed 27 May 2018).

3 World Health Organization. GLASS | Global antimicrobial resistance surveillance system (GLASS) report. Geneva: 2017.

4 Interagency Coordination Group on Antimicrobial Resistance. No Time to Wait: Securing the future from drug-resistant infections. Interagency Coordination Group on Antimicrobial Resistance 2019. https://www.who.int/antimicrobial-resistance/interagency-coordination-group/IACG_final_report_EN.pdf (accessed 18 Jul 2019).

5 Roberts RR, Hota B, Ahmad I, *et al.* Hospital and Societal Costs of Antimicrobial-Resistant Infections in a Chicago Teaching Hospital: Implications for Antibiotic Stewardship. *Clin Infect Dis* 2009;**49**:1175–84. doi:10.1086/605630

6 The Review on Antimicrobial Resistance. Antimicrobial Resistance: Tackling a Crisis For the Health and Wealth Of Nations. 2014.

7 Ahmed SA, Bariş E, Go DS, *et al.* Assessing the Global Economic and Poverty Effects of Antimicrobial Resistance. 2017.

8 Fleming-Dutra KE, Hersh AL, Shapiro DJ, *et al.* Prevalence of inappropriate antibiotic prescriptions among US ambulatory care visits, 2010-2011. *JAMA* 2016;**315**:1864–73.

9 Shively NR, Buehrle DJ, Clancy CJ, *et al.* Prevalence of Inappropriate Antibiotic Prescribing in Primary Care Clinics within a Veterans Affairs Health Care System. *Antimicrob Agents Chemother* 2018;**62**:e00337-18.

10 Cadieux G, Tamblyn R, Dauphinee D, *et al.* Predictors of inappropriate antibiotic prescribing among primary care physicians. *Can Med Assoc J* 2007;**177**:877–83.

11 Renwick MJ, Simpkin V, Mossialos E. Targeting innovation in antibiotic drug discovery and development: the need for a one health - one Europe - one world framework. 2016.http://www.ncbi.nlm.nih.gov/books/NBK447337/ (accessed 18 Feb 2020).

12 Dyar OJ, Huttner B, Schouten J, *et al.* What is antimicrobial stewardship? *Clin Microbiol Infect* 2017;**23**:793–8.

13 Nathwani D, Varghese D, Stephens J, *et al.* Value of hospital antimicrobial stewardship programs [ASPs]: a systematic review. *Antimicrob Resist Infect Control* 2019;**8**.

14 Davey P, Marwick CA, Scott CL, *et al.* Interventions to improve antibiotic prescribing practices for hospital inpatients. *Cochrane Database Syst Rev* Published Online First: 9 February 2017.

For peer review only

15 Bal AM, Gould IM. Antibiotic stewardship: Overcoming implementation barriers. *Curr Opin Infect Dis* 2011;**24**:357–62.

16 Allerberger F, Gareis R, Jindrák V, *et al.* Antibiotic stewardship implementation in the EU: the way forward. *Expert Rev Anti Infect Ther* 2009;**7**:1175–83.

17 Langford BJ, Schwartz KL, Garber GE. Antimicrobial stewardship by family physicians. *Can Fam Physician* 2018;**64**:168–9.

18 Drekonja D, Filice G, Greer N, *et al. Antimicrobial Stewardship Programs in Outpatient Settings: A Systematic Review*. Washington DC: Department of Veterans Affairs 2014. http://www.ncbi.nlm.nih.gov/books/NBK274571/ (accessed 19 Jul 2019).

19 Public Health Agency of Canada. Canadian Antimicrobial Resistance Surveillance System – 2017 Report. 2017.

20 Sanchez GV, Fleming-Dutra KE, Roberts RM, *et al.* Core Elements of Outpatient Antibiotic Stewardship. *MMWR Recomm Rep* 2016;**65**:1–12.

21 McEwen SA, Collignon PJ. Antimicrobial Resistance: a One Health Perspective. In: Schwarz S, Cavaco LM, Shen J, eds. *Antimicrobial Resistance in Bacteria from Livestock and Companion Animals*. Washington DC: ASM Press 2018. 521–47.

22 Lloyd DH, Page SW. Antimicrobial Stewardship in Veterinary Medicine. In: Schwarz S, Cavaco LM, Shen J, eds. *Antimicrobial Resistance in Bacteria from Livestock and Companion Animals*. Washington DC: ASM Press 2018. 675–97.

23 Pouwels KB, Hopkins S, Llewelyn MJ, *et al.* Duration of antibiotic treatment for common infections in English primary care: cross sectional analysis and comparison with guidelines. *BMJ* 2019;**364**:l440.

24 Smith SM, Fahey T, Smucny J, *et al.* Antibiotics for acute bronchitis. *Cochrane Database Syst Rev* Published Online First: 19 June 2017.

25 Little P, Stuart B, Francis N, *et al.* Effects of internet-based training on antibiotic prescribing rates for acute respiratory-tract infections: a multinational, cluster, randomised, factorial, controlled trial. *The Lancet* 2013;**382**:1175–82.

26 Bernal JL, Cummins S, Gasparrini A. Interrupted time series regression for the evaluation of public health interventions: a tutorial. *Int J Epidemiol* 2017;**46**:348–55.

27 University of Toronto Practice-Based Research Network. About UTOPIAN. 2017.http://www.dfcm.utoronto.ca/about-utopian (accessed 17 Feb 2019).

28 University of Toronto Practice-Based Research Network. UTOPIAN Data Safe Haven. 2019.http://www.dfcm.utoronto.ca/utopian-data-safe-haven (accessed 4 Sep 2019).

29 Clalit. The Story of Clalit Health Services. 2010.http://www.clalit-global.co.il/en/_the_story_of_clalit_health_services.html (accessed 21 Jul 2019).

For peer review only

30 Meeker D, Linder JA, Fox CR, *et al.* Effect of Behavioral Interventions on Inappropriate Antibiotic Prescribing Among Primary Care Practices: A Randomized Clinical Trial. *JAMA* 2016;**315**:562.

31 Groth A, Enoksson F, Hermansson A, *et al.* Acute mastoiditis in children in Sweden 1993–2007—No increase after new guidelines. *Int J Pediatr Otorhinolaryngol* 2011;**75**:1496–501.

32 Dosh SA, Hickner JM, Mainous AGI, *et al.* Predictors of Antibiotic Prescribing for Nonspecific Upper Respiratory Infections, Acute Bronchitis, and Acute Sinusitis. *J Fam Pract* 2000;**49**:409–15.

33 Taljaard M, McKenzie JE, Ramsay CR, *et al.* The use of segmented regression in analysing interrupted time series studies: an example in pre-hospital ambulance care. *Implement Sci* 2014;**9**.

34 Kontopantelis E, Doran T, Springate DA, *et al.* Regression based quasi-experimental approach when randomisation is not an option: interrupted time series analysis. *BMJ* 2015;**350**.

35 Arnold SR, Straus SE. Interventions to improve antibiotic prescribing practices in ambulatory care. *Cochrane Database Syst Rev* Published Online First: 2005.

36 Gerber JS, Prasad PA, Fiks AG, *et al.* Durability of Benefits of an Outpatient Antimicrobial Stewardship Intervention After Discontinuation of Audit and Feedback. *JAMA* 2014;**312**:2569–70.

37 Ebell MH, Radke T. Antibiotic Use for Viral Acute Respiratory Tract Infections Remains Common. *Am J Manag Care* 2015;**21**:9.

38 Whitehead J. *The design and analysis of sequential clinical trials*. Rev. 2nd ed. Chichester, UK: John Wiley & Sons 1997.

39 American Academy of Family Physicians. AAFP Member Survey Provides Valuable Perspective. 2017.https://www.aafp.org/news/blogs/inthetrenches/entry/20170801ITT_Survey.html (accessed 27 Feb 2020).

40 Harris PA, Taylor R, Thielke R, *et al.* Research electronic data capture (REDCap)—A metadata-driven methodology and workflow process for providing translational research informatics support. *J Biomed Inform* 2009;**42**:377–81.

For peer review only

AUTHORS' CONTRIBUTIONS

J-PRS, ML, KRA, ME, AH, SL, GEG, WW, JM-G, DNF, and DRM conceived of the study and the analysis. J-PRS wrote the initial manuscript and will conduct the data analysis. All authors contributed to and approved the final manuscript.

FUNDING STATEMENT

This work was supported by the European Commission's Joint Programming Initiative on Antimicrobial Resistance (Swedish Research Council: 2017-05972, Canadian Institutes of Health Research: AMR - 155212, Israeli Ministry of Health: 8762491).

COMPETING INTERESTS STATEMENT

The authors declare no conflicts of interest.

For peer review only

Tables

Table 1. Composition of the feedback reports (intervention) at each time point.

Feedback report	Content of report	Benchmarking figure	Guidelines
Report at t_0	1. Antimicrobial prescribing rate/100 visits in the previous year for all visits.<br>2. Proportion of antimicrobial prescriptions in the previous year with duration over 7 days.	Provider's own rate/proportion versus average rate/proportion and 25th percentile rate/proportion across all participants at the same study site.	Guidelines indicating that antimicrobial prescribing longer than 7 days is not necessary for many conditions.
Report at t_3	1. Antimicrobial prescribing rate/100 visits in the previous year for viral respiratory conditions.	Provider's own rate versus average rate and 25th percentile rate across all participants at the same study site.	Prescribing guidelines for viral respiratory conditions.
Report at t_6	1. Antimicrobial prescribing rate/100 visits in the previous year for acute sinusitis.	Provider's own rate versus average rate and 25th percentile rate across all participants at the same study site.	Prescribing guidelines for acute sinusitis.

For peer review only

Table 2. Covariates included in the interrupted time-series analysis of the OPEN Stewardship study. Excludes variables related to the effect of the COVID-19 pandemic.

Variable	Purpose	Form	Description
Number of prescriptions	Outcome	Integer	Number of antimicrobial prescriptions for given indication
Number of visits for indication	Offset	Integer	Number of patient visits for given indication
Study site	Covariate	Binary	Southern Israel versus Ontario, Canada (baseline)
Practice group	Random intercept	ID	Random intercept for practice group (nested within region)
Provider	Random intercept	ID	Random intercept for provider (nested within practice group)
Month	Covariate	Categorical	Calendar month (baseline = January)
Month × study site	Interaction	Interaction	Allows different value for coefficients for Southern Israel
Physician age	Covariate	Integer	Physician age at t_{-12}
Physician age × study site	Interaction	Interaction	Allows different value for coefficient for Southern Israel
Physician sex	Covariate	Binary	Female versus male (baseline)
Physician sex × study site	Interaction	Interaction	Allows different value for coefficient for Southern Israel
Physician employment status	Covariate	Binary	Part-time versus full-time (baseline)
Physician employment status × study site	Interaction	Interaction	Allows different value for coefficient for Southern Israel
Percent female patients	Covariate	Continuous	Percentage of visits by female patients
Percent female patients × study site	Interaction	Interaction	Allows different value for coefficient for Southern Israel
Percent patients 17 and under	Covariate	Continuous	Percentage of visits by patients 17 and younger
Percent patients 17 and under × study site	Interaction	Interaction	Allows different value for coefficient for Southern Israel
Percent patients 65 and older	Covariate	Continuous	Percentage of visits by patients 65 and older
Percent patients 65 and older × study site	Interaction	Interaction	Allows different value for coefficient for Southern Israel
Post-intervention	Intervention	Binary	1 if provider is in intervention group and intervention has occurred, 0 otherwise; level change of the outcome associated with intervention
Post-intervention × study site	Interaction	Interaction	Allows different value for level change of the outcome for Southern Israel
Time	Trend	Integer	Time since the beginning of the study; slope of the time trend of the outcome
Time × study site	Interaction	Interaction	Allows different value for slope of the time trend of the outcome for Southern Israel
Time × post-intervention	Intervention effect	Interaction	Change in slope of time trend in the intervention group after intervention is received
Time × post-intervention × study site	Interaction	Interaction	Allows different value for change in slope of time trend in the intervention group after intervention is received for Southern Israel

For peer review only

Table 3. Timing of analyses for objective 1–4 of the study. The definitions of the pre-intervention and post-intervention periods are dependant on the delivery time of the report (intervention) related to each objective.

Objective	Outcome	Unit of analysis	Intervention time	Number of observations in intervention cohort		Total observations/ observations in control cohort
				Pre-intervention	Post-intervention	
1	Prescribing for all indications	Provider	t_0	12	12	24
2	Prescribing (for all indications) with duration longer than 7 days	Provider	t_0	12	12	24
3	Prescribing for viral respiratory conditions	Provider	t_3	15	9	24
4	Prescribing for acute sinusitis	Provider	t_6	18	6	24

For peer review only

Figure legends

Figure 1. Example of chart sent in the first feedback report, representing the provider's prescribing rate/100 visits for any indication versus the average rate and 25th percentile rate across all providers in the study from the same study site.

Figure 2. Pre-intervention and post-intervention periods for objectives 1–4 of the OPEN Stewardship study. A: Objective 1, overall prescribing (report sent at t_0). B: Objective 2, prescribing with duration longer than 7 days (report sent at t_0). C: Objective 3, prescribing for viral respiratory conditions (report sent at t_3). D: Objective 4, prescribing for acute sinusitis (report sent at t_6). t_0 represents the time of the first report (intervention); t_{-12} represents 1 year prior to the first report; t_{12} marks the conclusion of the study period (1 year after the first report).

For peer review only

Number of Prescriptions of Any Antibiotic (Per 100 Visits) for Any Indication

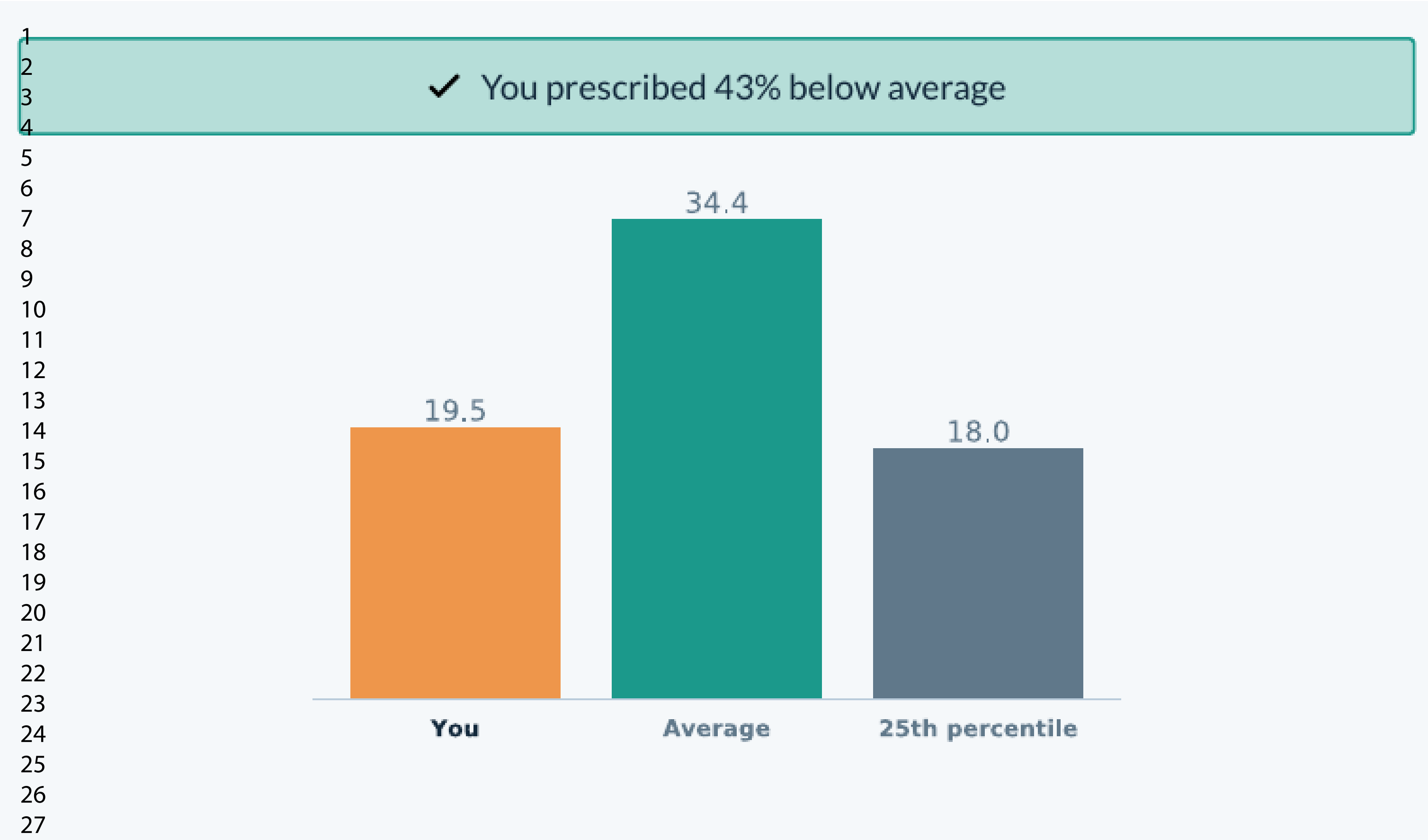

You had 865 patient visits from Jan 01, 2019 to Dec 31, 2019 .

These data are not adjusted for physician's practice characteristics, nor do they differentiate between delayed or non-filled prescriptions. These results are generated from claims data, and misclassification of the diagnosis may be present. As a result, our prescribing targets are referenced to peer benchmarks. Learn more about the data and its interpretation.

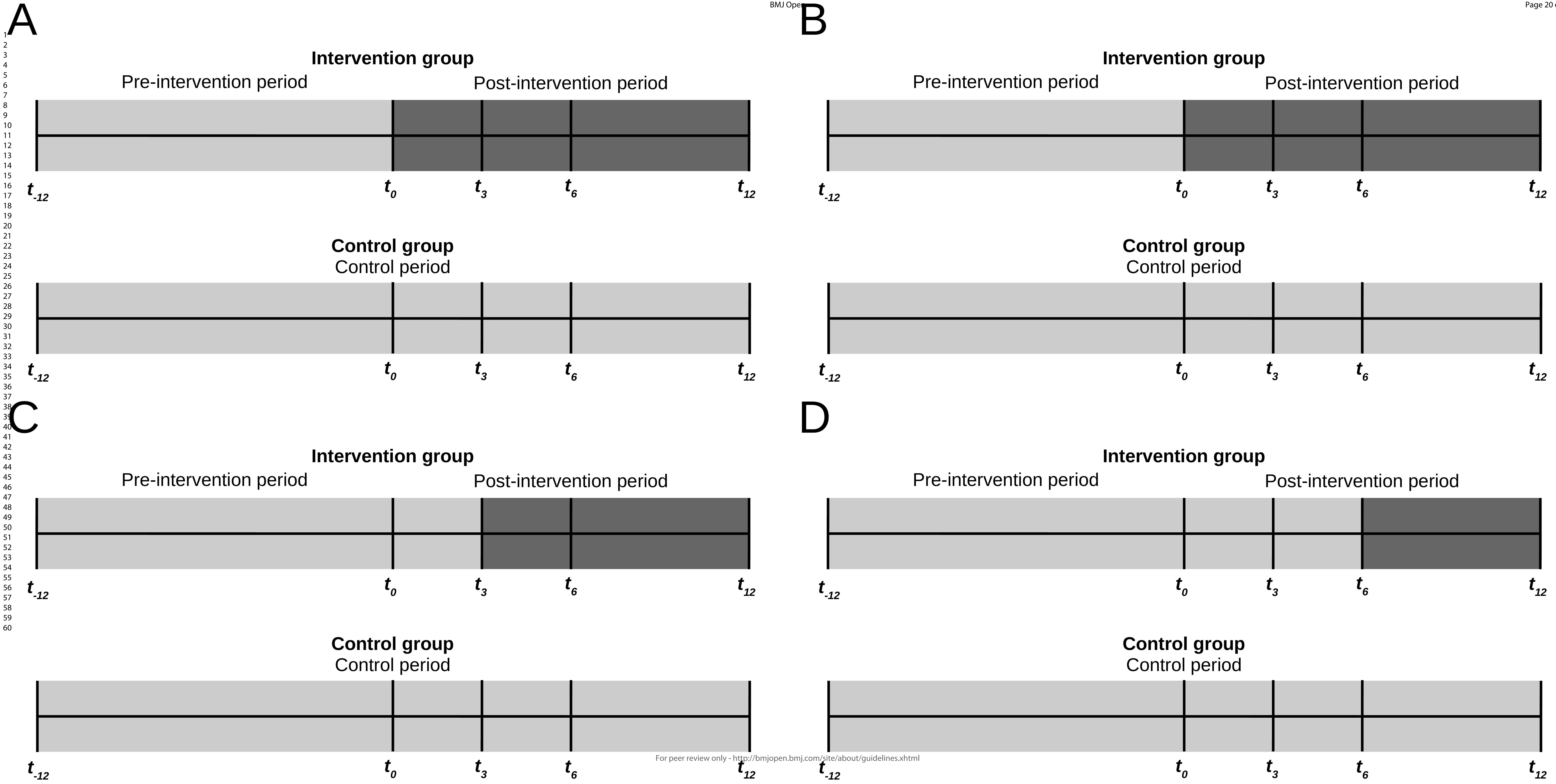
A
Intervention group
Pre-intervention period
Post-intervention period
t-12
t0
t3
t6
t12
Control group
Control period
t-12
t0
t3
t6
t12
B
Intervention group
Pre-intervention period
Post-intervention period
t-12
t0
t3
t6
t12
Control group
Control period
t-12
t0
t3
t6
t12
C
Intervention group
Pre-intervention period
Post-intervention period
t-12
t0
t3
t6
t12
Control group
Control period
t-12
t0
t3
t6
t12
D
Intervention group
Pre-intervention period
Post-intervention period
t-12
t0
t3
t6
t12
Control group
Control period
t-12
t0
t3
t6
t12

Table S1. ICD-9 codes for indications identified as acute sinusitis and viral respiratory conditions in the OPEN Stewardship platform.

OPEN Stewardship category	Indication	ICD-9 codes
Acute sinusitis	Acute sinusitis	461 Acute sinusitis
Viral respiratory conditions	Asthma/allergy	477 Allergic rhinitis, 493 Asthma, 995.3 Allergy, unspecified
	Bronchitis/bronchiolitis	466 Acute bronchitis and bronchiolitis, 490 Bronchitis, not specified as acute or chronic, 492 Emphysema, 496 Chronic airway obstruction, not elsewhere classified
	Influenza	487 Influenza, 488 Influenza due to identified avian influenza virus
	Viral pneumonia	480 Viral pneumonia
	Viral upper respiratory tract infection	460 Acute nasopharyngitis [common cold], 464 Acute laryngitis and tracheitis, 465 Acute upper respiratory infections of multiple or unspecified sites, 786.2 Cough

For peer review only

Appendix 1. Survey questions for participants in the OPEN Stewardship trial (English).

Introductory text: Thank you for agreeing to receive the OPEN Stewardship report on antibiotic prescribing. Help us improve future reports by filling out this short survey. It will only take 3-5 minutes. Questions are multiple choice with the option to provide comments at the end of the survey. Your feedback is important to us and your opinion matters!

Survey 1 (t_0)

Q1. Is the purpose of the OPEN Stewardship report clear?

- Yes
- No
- Don't know

Q2. How informative did you find the following item in the OPEN Stewardship report: Your Prescribing Rate per Visit Compared to Peers (example below).

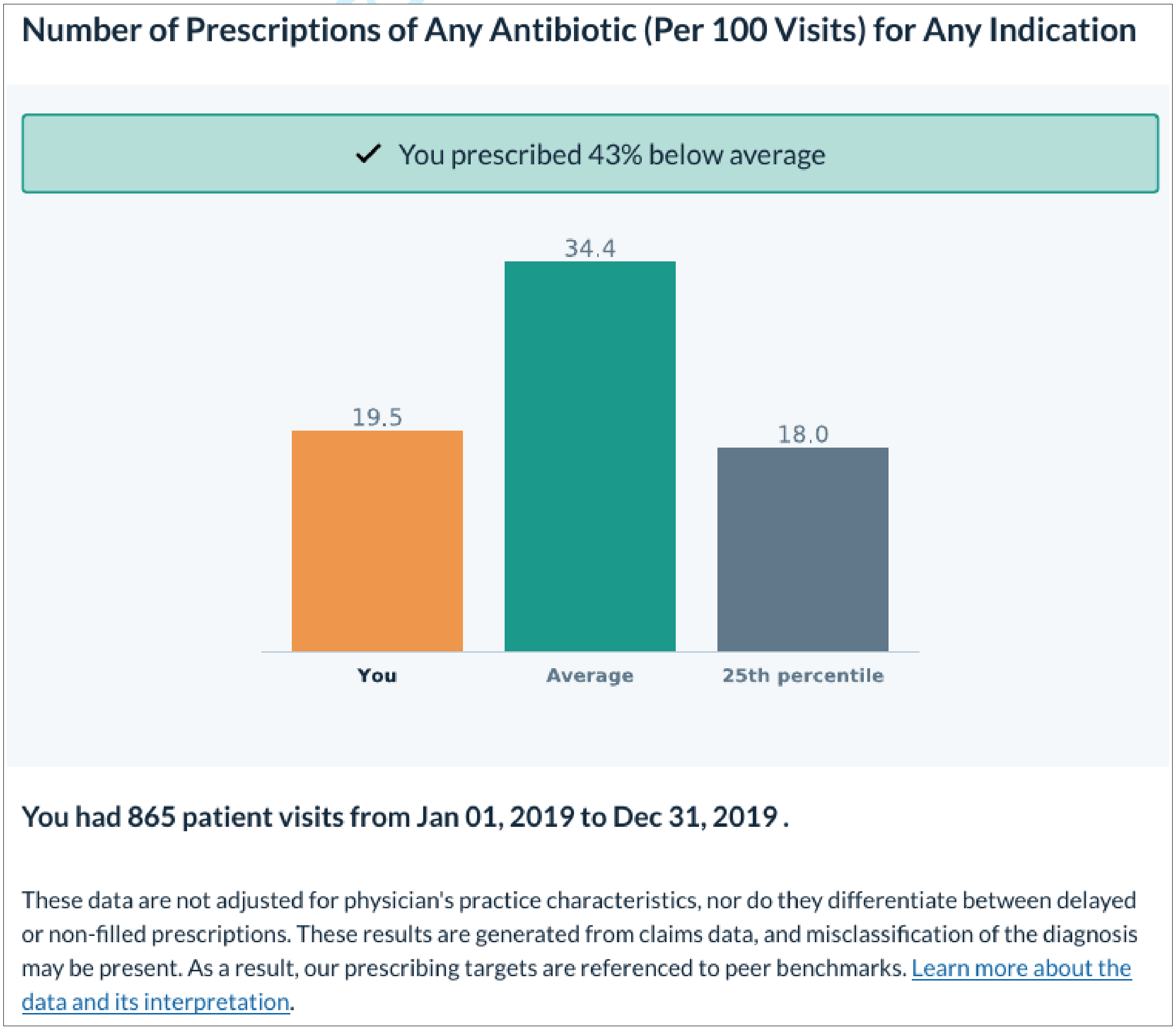

- Very informative

- Somewhat informative
- Not so informative
- Not at all informative
- Don’t know

Q3. Was it straightforward how to navigate through the OPEN Stewardship report?

- Yes
- No
- Don’t know

Q4. Is it useful to include *Best Practice Guidelines* in the OPEN Stewardship report (sample from most recent report below)?

Acute sinusitis (Adult)

COMMON PATHOGENS

Viruses | Streptococcus pneumoniae (non-meningitis) | Moraxella sp. | Haemophilus sp.

RECOMMENDED TREATMENT

First Line

Treatment Regimen Option

Drug	Dose	Frequency	Route	Duration
**No Antibiotic Treatment Indicated**	. mg	Once	PO	. days

Most acute sinusitis is viral in origin and does not require antibiotics. Antibiotics may be considered if symptoms are persistent and not improving after >10 days, are severe for >3 days, or improved then worsened and lasting >3 days. See below for 1st line antibiotic treatment if there is an indication.

Treatment Regimen Option

Drug	Dose	Frequency	Route	Duration
**Amoxicillin**	500 mg	TID	PO	5-7 days

- Yes

- No
- Don’t know

Q5. Rate each of the following items according to its potential usefulness to you in an OPEN Stewardship report. Note that not all of these items were included in the report sent to you. (Very useful, Somewhat useful, Not so useful, Not at all useful, Don’t know)

- See how my prescribing changes over time
- Compare my own prescribing with peers
- Get an overview of local prescribing patterns
- Get an overview of local resistance levels
- Compare my own prescribing with guidelines
- Have discussions with peers on the local situation regarding prescribing patterns

Q6. Overall, how would you rate the usefulness of the OPEN Stewardship report?

- Very useful
- Somewhat useful
- Not so useful
- Not at all useful
- Don’t know

Q7. Please suggest how to improve the OPEN Stewardship report (such as layout of the graphs and information included).

[Free text box]

Q8. Any additional comments?

[Free text box]

Survey 2 (t_3)

Q1. How informative did you find the following item in the OPEN Stewardship report: Your Prescribing Rate per Visit Compared to Peers (example below).

For peer review only

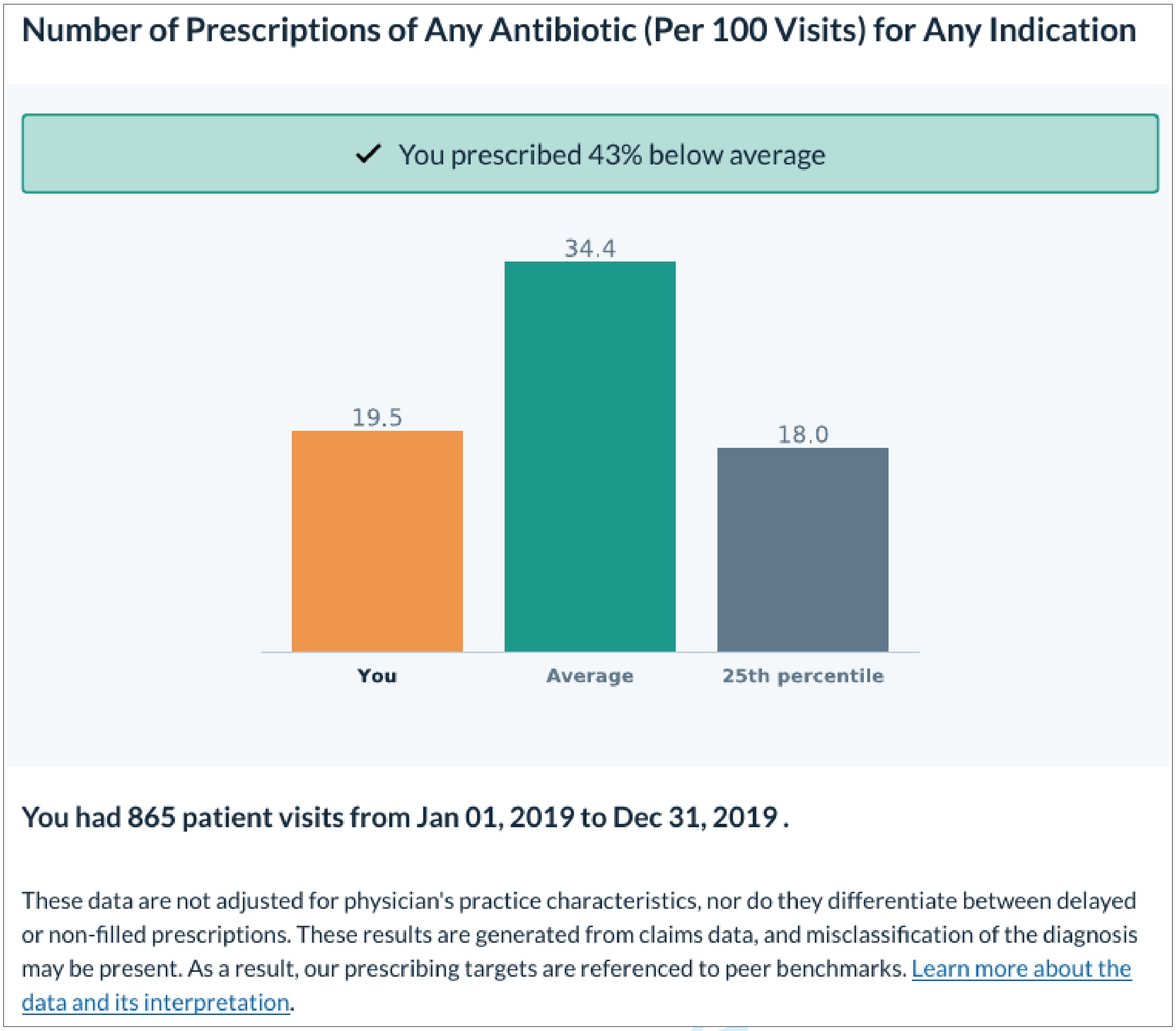

- Very informative
- Somewhat informative
- Not so informative
- Not at all informative
- Don't know

Q2. In the past three months, did you ever consider the previous OPEN Stewardship report when making an antibiotic prescribing decision?

- Yes
- No
- Don't know

Q3. Rate each of the following items according to its potential usefulness to you in the OPEN Stewardship report. (Very useful, Somewhat useful, Not so useful, Not at all useful, Don't know)

- See how my prescribing changes over time
- Compare my own prescribing with peers
- Get an overview of local prescribing patterns

For peer review only - http://bmjopen.bmj.com/site/about/guidelines.xhtml

- Compare my own prescribing with guidelines
- Have discussions with peers on the local situation regarding prescribing patterns

Q4. Overall, how would you rate the usefulness of the OPEN Stewardship report?

- Very useful
- Somewhat useful
- Not so useful
- Not at all useful
- Don't know

Q5. Please suggest how to improve the OPEN Stewardship report (such as layout of the graphs and information included).

[Free text box]

Q6. Any additional comments?

[Free text box]

Survey 3 (t_6)

Q1. How informative did you find the following item in the OPEN Stewardship report: Your Prescribing Rate per Visit Compared to Peers (example below).

For peer review only

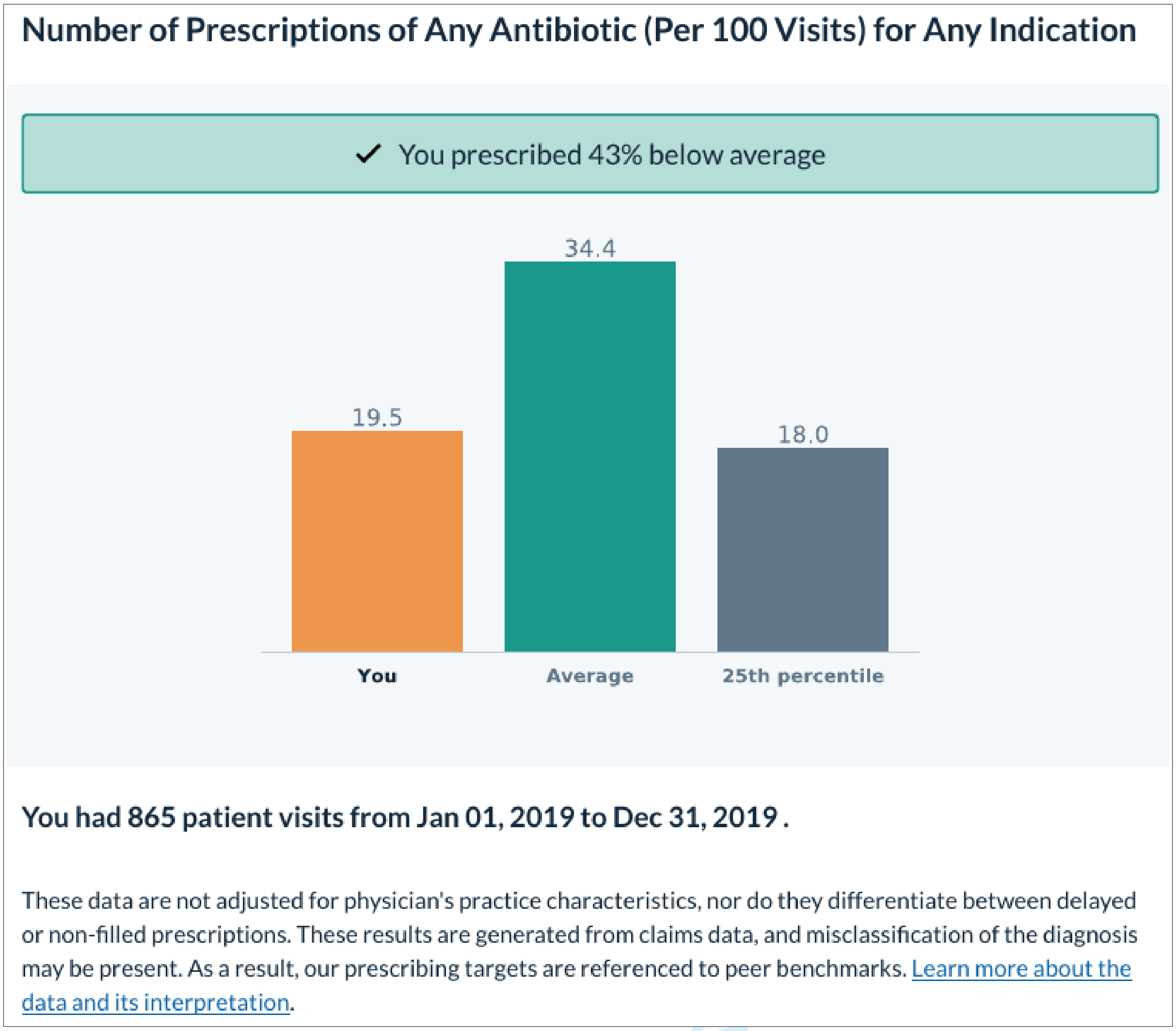

- Very informative
- Somewhat informative
- Not so informative
- Not at all informative
- Don’t know

Q2. In the past three months, did you ever consider the previous OPEN Stewardship report when making an antibiotic prescribing decision?

- Yes
- No
- Don’t know

Q3. Overall, how would you rate the usefulness of the OPEN Stewardship report?

- Very useful
- Somewhat useful
- Not so useful
- Not at all useful

For peer review only - http://bmjopen.bmj.com/site/about/guidelines.xhtml

- Don’t know

Q4. Hypothetically, how often do you think it would be appropriate to receive OPEN Stewardship reports?

- Monthly
- Quarterly
- Every six months
- Annually
- Not at all
- Don’t know

Q5. Would a central repository of local treatment guidelines be useful for your practice?

- Yes
- No
- Don’t know

Q6. Do you think it would be useful to have access to a personalized web-portal where you could browse your own and local patterns of antibiotic use, as well as best practice guidelines?

- Yes
- No
- Don’t know

Q7. Would you be willing to participate in a follow-up interview where we would ask in more detail about your experiences with the OPEN Stewardship reports? If so, please send an email to [study email address].

Q8. Please suggest how to improve the OPEN Stewardship report (such as layout of the graphs and information included).

[Free text box]

Q9. Any additional comments?

[Free text box]

For peer review only

Appendix 2. Survey questions for participants in the OPEN Stewardship trial (Hebrew).

Introductory text:

רופא יקר,

אנו מודים לך שוב על הסכמתך לקחת חלק במחקר המשותף של אגף רפואה בחטיבת הקהילה בשירותי בריאות כללית והמחלקה לניהול מערכות בריאות באוניברסיטת בן גוריון בנגב בנושא "דפוסי רישום תרופות אנטיביוטיות על-ידי רופאי משפחה".

נודה לך אם תוכל להקדיש שלוש עד חמש דקות מזמנך כדי לענות על השאלון שלהלן, המתייחס לדו"ח האישי שנשלח אליך לאחרונה בנושא מרשמי האנטיביוטיקה של מטופליך. תשובותיך יעזרו לנו לשפר את אופן העברת המידע לרופאים בהמשך.

השאלון נכתב בלשון זכר מטעמי נוחות, אך מיועד לשני המינים.

Survey 1 (t_0)

שאלה 1: האם מטרת הדו"ח האישי ששלחנו לך, ברורה, לדעתך?

- כן
- לא
- לא יודע

שאלה 2: באיזו מידה הפריט הבא – "שיעור מרשמי האנטיביוטיקה שרשמת פר ביקור מטופל בהשוואה לעמיתיך" - סיפק לך את המידע הדרוש לך? (ראה דוגמה להלן)

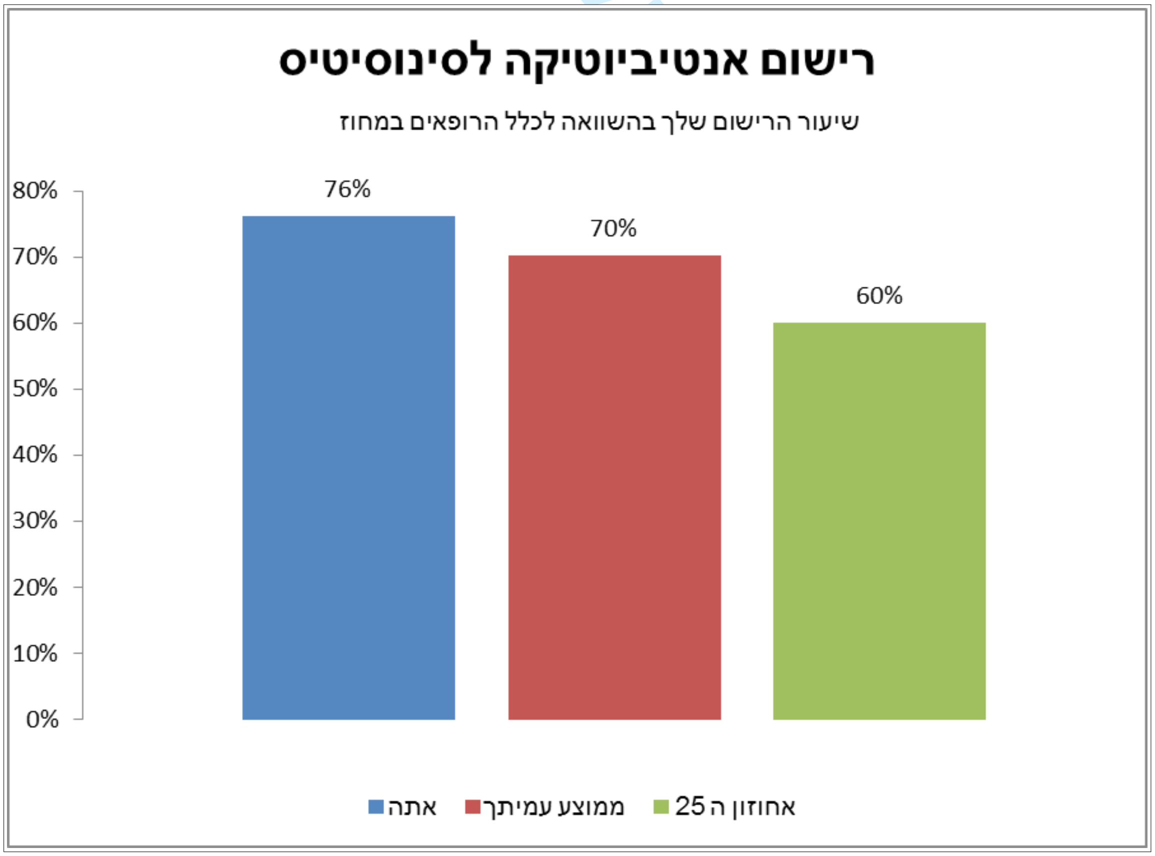

For peer review

- מידה רבה
- במידה מסוימת
- במידה מועטה
- בכלל לא
- לא יודע

שאלה 3: האם הדוח האישי היה קל להבנה?

- כן
- לא
- לא יודע

שאלה 4: האם יש, לדעתך, תועלת בצירוף קווים מנחים לדו"ח האישי (ראה דוגמה להלן)?

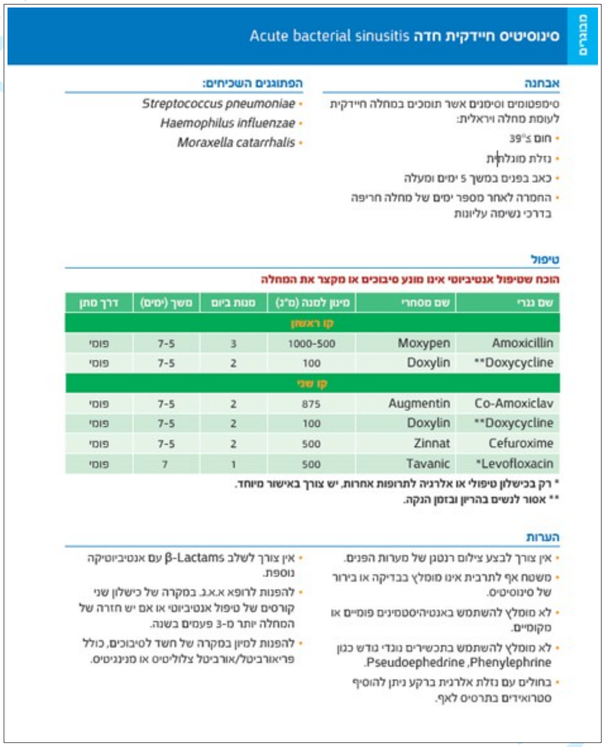

מבוגרים

סינוסיטיס חיידקית חדה Acute bacterial sinusitis

אבחנה

סימפטומים וסימנים אשר תומכים במחלה חיידקית לעומת מחלה ויראלית:

- חום ≥39°
- נזלת מוגלתית
- כאב בפנים במשך 5 ימים ומעלה
- החמרה לאחר מספר ימים של מחלה חריפה בדרכי נשימה עליונות

הפתוגנים השכיחים:

- *Streptococcus pneumoniae*
- *Haemophilus influenzae*
- *Moraxella catarrhalis*

טיפול

הוכח שטיפול אנטיביוטי אינו מונע סיבוכים או מקצר את המחלה

שם גנרי	שם מסחרי	מינון למנה (מ"ג)	מנות ביום	משך (ימים)	דרך מתן
קו ראשון					
Amoxicillin	Moxypen	1000-500	3	7-5	פומי
**Doxycycline	Doxylin	100	2	7-5	פומי
קו שני					
Co-Amoxiclav	Augmentin	875	2	7-5	פומי
**Doxycycline	Doxylin	100	2	7-5	פומי
Cefuroxime	Zinnat	500	2	7-5	פומי
*Levofloxacin	Tavanic	500	1	7	פומי

*** רק בכישלון טיפולי או אלרגיה לתרופות אחרות, יש צורך באישור מיוחד.**
**** אסור לנשים בהריון ובזמן הנקה.**

הערות

- אין צורך לבצע צילום רנטגן של מערות הפנים.
- משטח אף לתרבית אינו מומלץ בבדיקה או בירור של סינוסיטיס.
- לא מומלץ להשתמש באנטיהיסטמינים פומיים או מקומיים.
- לא מומלץ להשתמש בתכשירים נוגדי גודש כגון Phenylephrine, Pseudoephedrine.
- בחולים עם נזלת אלרגית ברקע ניתן להוסיף סטרואידים בתרסיס לאף.
- אין צורך לשלב β-Lactams עם אנטיביוטיקה נוספת.
- להפנות לרופא א.א.ג. במקרה של כישלון שני קורסים של טיפול אנטיביוטי או אם יש חזרה של המחלה יותר מ-3 פעמים בשנה.
- להפנות למיון במקרה של חשד לסיבוכים, כולל פריאורביטל/אורביטל צלוליטיס או מנינגיטיס.

- כן
- לא
- לא יודע

שאלה 5: אנא הענק ציון לכל אחד מההיגדים הבאים, בהתאם לתועלת הפוטנציאלית עבורך בדו"ח האישי ששלחנו לך שים לב, לא כל המשפטים/היגדים שאתה נשאל עליהם הופיעו בדו"ח האישי שקבלת. (מועיל מאוד, מועיל במידה מסוימת, מועיל במידה מועטה, כלל לא מועיל, לא יודע)

- אני יכול לראות כיצד דפוס רישום האנטיביוטיקה שלי משתנה לאורך זמן
- אני יכול להשוות את דפוס רישום האנטיביוטיקה שלי לאלה של עמיתיי
- אני יכול לקבל סקירה של דפוסי המרשם המקומיים
- קבלת דוח סקירה מקומי על רמות עמידות
- אני יכול להשוות את דפוס הרישום שלי לעומת הקווים המנחים של הכללית
- מאפשר לי לדון עם עמיתיי על מצב דפוסי המרשם המקומיים

שאלה 6: בסך הכל, איזה ציון היית מעניק לתועלת שאתה מפיק מהדו"ח האישי?
- מועיל מאוד
- מועיל במידה מסוימת
- מועיל במידה מועטה
- כלל לא מועיל
- לא יודע

שאלה 7: אנא הצע דרכים לשיפור הדו"ח האישי בנוגע להיבטים הבאים (עיצוב הגרף והפרמטרים).

[טקסט חופשי]

שאלה 8: הערות נוספות?

[טקסט חופשי]

Survey 2 (t_3)

שאלה 1: באיזו מידה הפריט הבא – "שיעור מרשמי האנטיביוטיקה שרשמת פר ביקור מטופל בהשוואה לעמיתיך" - סיפק לך את המידע הדרוש לך? (ראה דוגמה להלן)

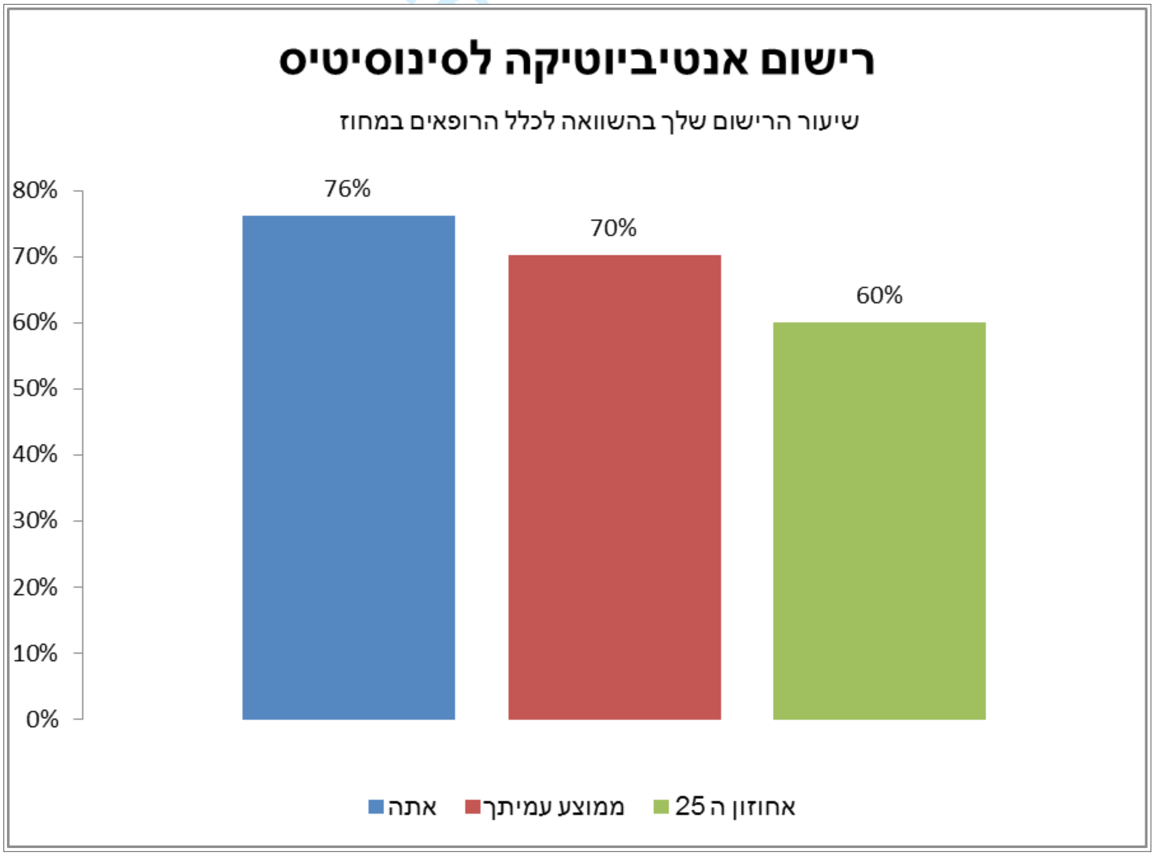

- מידה רבה
- במידה מסוימת
- במידה מועטה
- בכלל לא
- לא יודע

שאלה 2: האם במהלך שלושת החודשים האחרונים לקחת בחשבון את תוצאות הדוח הקודם לצורך קבלת החלטה על רישום אנטיביוטיקה למטופל?

- כן
- לא
- לא יודע

שאלה 3: אנא הענק ציון לכל אחד מההיגדים הבאים, בהתאם לתועלת הפוטנציאלית עבורך בדו"ח האישי ששלחנו לך. (מועיל מאוד, מועיל במידה מסוימת, מועיל במידה מועטה, כלל לא מועיל, לא יודע)

- אני יכול לראות כיצד דפוס רישום האנטיביוטיקה שלי משתנה לאורך זמן
- אני יכול להשוות את דפוס רישום האנטיביוטיקה שלי לאלה של עמיתיי
- אני יכול לקבל סקירה של דפוסי המרשם המקומיים
- אני יכול להשוות את דפוס הרישום שלי לעומת הקווים המנחים של הכללית
- מאפשר לי לדון עם עמיתיי על מצב דפוסי המרשם המקומיים

שאלה 4: בסך הכל, איזה ציון היית מעניק לתועלת שאתה מפיק מהדו"ח האישי?

- מועיל מאוד
- מועיל במידה מסוימת
- מועיל במידה מועטה
- כלל לא מועיל
- לא יודע

שאלה 5: אנא הצע דרכים לשיפור הדו"ח האישי בנוגע להיבטים הבאים (עיצוב הגרף והפרמטרים).

[טקסט חופשי]

שאלה 6: הערות נוספות?

[טקסט חופשי]

Survey 3 (t_6)

שאלה 1: באיזו מידה הפריט הבא – "שיעור מרשמי האנטיביוטיקה שרשמת פר ביקור מטופל בהשוואה לעמיתיך" - סיפק לך את המידע הדרוש לך? (ראה דוגמה להלן)

For peer review only

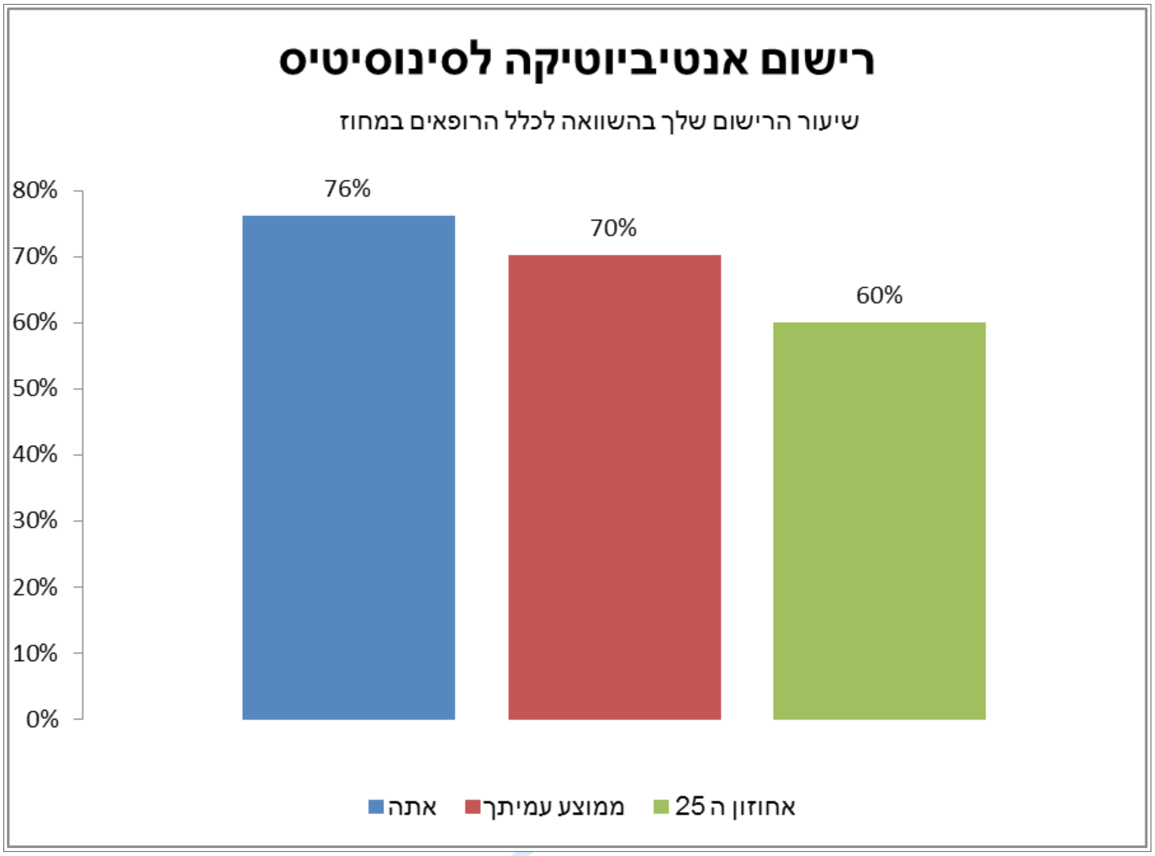

- מידה רבה
- במידה מסוימת
- במידה מועטה
- בכלל לא
- לא יודע

שאלה 2: האם במהלך שלושת החודשים האחרונים לקחת בחשבון את תוצאות הדוח הקודם לצורך קבלת החלטה על רישום אנטיביוטיקה למטופל?

- כן
- לא
- לא יודע

שאלה 3: בסך הכל, איזה ציון היית מעניק לתועלת שאתה מפיק מהדו"ח האישי?

- מועיל מאוד
- מועיל במידה מסוימת
- מועיל במידה מועטה
- כלל לא מועיל
- לא יודע

שאלה 4: באופן תיאורטי, מהי התדירות הרצויה לדעתך לקבלת הדו"ח האישי?

- אחת לחודש
- אחת לרבעו
- אחת לחצי שנה
- אחת לשנה
- בכלל לא

שאלה 5: האם מאגר מרכזי של קווים מנחים מקומיים עשוי להועיל לך בעבודתך?

- כן
- לא
- לא יודע

שאלה 6: האם אתה עשוי למצוא תועלת בקבלת גישה לפורטל אישי ברשת האינטרנט, בו תוכל למצוא את דפוסי רישום האנטיביוטיקה האישיים שלך וכן את דפוסי רישום האנטיביוטיקה המקומיים, בצירוף הקווים המנחים הרלוונטיים?

- כן
- לא
- לא יודע

שאלה 7: האם תהיה מוכן להשתתף בראיון עוקב (follow-up), בו תישאל על ניסיונך עם הדו"חות האישיים בצורה מפורטת יותר?

- כן
- לא
- לא יודע

שאלה 8: אנא הצע דרכים לשיפור הדו"ח האישי בנוגע להיבטים הבאים (עיצוב הגרף והפרמטרים).

[טקסט חופשי]

שאלה 9: הערות נוספות?

[טקסט חופשי]

For peer review only

Appendix 3. OPEN Stewardship feedback survey pilot protocol and interview guide.

OPEN Stewardship Survey: Pilot testing of questionnaires #1, #2 and #3
***** INTERVIEW GUIDE AND TEMPLATE FOR RESPONSES *****

Purpose of the pilots

- To identify issues (clarity, technical errors etc) in the questionnaires and find potential solutions to improve the questionnaires, key points being:
  - Do the respondents understand the purpose of the questionnaire?
  - Do the respondents find the questions relevant?
  - Do the respondents think that there are questions that are missing?
- To get a realistic estimate of the response time.

Before the interview

- **Identify respondents and book time**
- Identify two respondents from each country and sector (humans/companion animals/dairy) who are willing to pilot test the questionnaire, either among those that have been recruited to the OPEN Stewardship study or other similar persons (or a combination of both), do what is most feasible.
- If possible, identify pilot respondents who are geographically close to you so that you can sit next to the respondent when conducting the pilot interview. An interview over phone, Skype or similar is acceptable but a less preferred option.
- If possible, of the two persons per country and sector, select one person with more years with relevant practice and one with less.
- Each pilot respondent will go through two questionnaires: one which is identical to questionnaire #1 and one which includes only those questions that are added to questionnaire #2 and to questionnaire #3 (referred to as pilot questionnaire #2)
  Estimated time for the pilot session is 60 minutes plus introduction and wrap up time, in total up to 90 minutes, so book 1.5 hour for the interview.

- **Communicate with each respondent before the interview**
- Send a (generic) OPEN Stewardship report one week before the booked interview.
- Ask the respondent to spend about 10 minutes to read it at some point before the booked interview.
- Ask the respondent to make a note of the date they read the report.
- Inform the respondent that during the interview you will go through an online questionnaire on his or her computer/device. You will also email some information during the interview. The respondent must thus have internet access plus access to email.

- **Material the interviewer should bring to the interview**
- Laptop or similar, with:
  - This guide in electronic format
  - Draft emails with link to pilot questionnaires to send during the interview
- A printed version of the introductory text that will be sent with each questionnaire
- Pen and paper
- Watch.

During the interview

For peer review only

Use this template during the interview as a guideline for the information that should be given to the respondent. Record the responses to the interview questions in the boxes as specified.
Please fill out and save one template per respondent.
Name each file: COUNTRY_SECTOR_ABBREVIATED-NAME-OF-RESPONDENT (first two letters of given name and first two letters of family name).
You may of course use your own words whenever you wish.

Name of interviewer
Fill out here

Abbreviated name of respondent (first two letters of given name and family name respectively)
Fill out here

- **INTRODUCTION TO INTERVIEW**

Thank you for taking the time to do this interview. As I mentioned previously, I estimate that it will take about 60 minutes.
The aim of the OPEN Stewardship project is to improve appropriate antibiotic prescribing by generating customised reports of individual prescribing in relation to peers. We sent you one of these reports a week ago.
Surveys will be sent out to practitioners enrolled in the OPEN Stewardship intervention at three occasions, to improve the reports.
We are now doing interviews to find out what respondents think about the survey questions. This is to ensure that the surveys measure the right things, so that we will get as good results as possible in order to improve our system. The surveys will be sent out by email and will be filled out online.
Just so you know, I am not the one who have made the survey questions we are about to go through. All of your comments are greatly appreciated.
Approximately 10 people will be interviewed about the survey questions in this pilot. This is an international collaboration, and we work together with colleagues in Canada, Israel, Sweden and the USA [REMOVE YOUR OWN COUNTRY].
If you need a break at any time during the interview just let me know.
Any questions before we get started?

For peer review only

- **SAMPLE REPORT INFORMATION**

Have you had time to read the OPEN Stewardship report?
Interviewer fill out here

- *If not, allow for up to 10 minutes to look at it (but don't rush the person).*

Roughly, when did you read the report? *Or note if the respondent is reading it right now*
Interviewer fill out here

- **EMAIL WITH QUESTIONNAIRE INFORMATION**

So we will start by looking at the text that will accompany the questionnaire, which will be sent out through a link in an email. If you can please read through this email.

- Hand over the paper with the email text. *If the interview is done over the phone, email the text to the respondent now.*

Do you have any comments?
Interviewer fill out here
Is there anything you feel is unclear?
Interviewer fill out here

Do you think the text would motivate someone to answer the survey?
Interviewer fill out here
Any suggestions?
Interviewer fill out here

Thank you!

- **QUESTIONNAIRE 1**

We will now move on to the first questionnaire, and I will email you a link to this questionnaire.

- Email the link to pilot questionnaire 1.

I would like to ask you to fill out the whole questionnaire, but don't press "submit" at the end. Take your time. If you wish you can make notes if there is anything that you come to think of; we will later go through the questionnaire question by question.

For peer review only

- Give the respondent a piece of paper and a pen.
- Respondent fills out the questionnaire.
- Note how long it takes in minutes to complete questionnaire.

Interviewer fill out here

- Note if the respondent seems to spend a longer time reading/re-reading any of the questions.

Interviewer fill out here

Thanks!
So let us start from the beginning with the introduction to the survey.

Do you think this text is informative enough? Any suggestions?
Interviewer fill out here

We will now go through each question, one at a time. I am interested in your opinion, in this situation there is nothing that is right or wrong. So we start with the first question.

- In your discussion with the respondent, note also if anything he or she says that is unexpected/odd.

- **"Is the purpose of the OPEN Stewardship report clear?"**

Can you please describe why you gave this response?
Interviewer fill out here

- **"How informative did you find the following item in the OPEN Stewardship report: Your Prescribing Rate per Visit Compared to Peers"**

Can you please describe why you gave this response?
Interviewer fill out here

Is it clear to you what the various responses mean?
Interviewer fill out here

- **"Was it straightforward how to navigate through the OPEN Stewardship report?"**

Can you please describe why you gave this response?
Interviewer fill out here

For peer review only

- **"Is it useful to include Best Practice Guidelines in the OPEN Stewardship report"**

Can you please describe why you gave this response?
Interviewer fill out here

- **"Rate each of the following items according to its potential usefulness to you in the OPEN Stewardship report."**

- **"See how my prescribing changes over time"**

Can you please describe why you gave this response?
Interviewer fill out here

- **"Compare my own prescribing with peers"**

Can you please describe why you gave this response?
Interviewer fill out here

- **"Get an overview of local prescribing patterns"**

Can you please describe why you gave this response?
Interviewer fill out here

- **"Compare my own prescribing with guidelines"**

Can you please describe why you gave this response?
Interviewer fill out here

- **"Have discussions with my peers on the local situation … "**

Can you please describe why you gave this response?
Interviewer fill out here

Is it clear to you what the various responses mean?
Interviewer fill out here

- **"Overall, how would you rate the usefulness of the OPEN Stewardship report?"**

For peer review only

Can you please describe why you gave this response?
Interviewer fill out here

- **"Please suggest how to improve the OPEN Stewardship report:"**
- **"Layout of the graphs (such as colors, size, legends)."**

Is it clear to you what kind of responses could be given here?
Interviewer fill out here

- **"Information included (such as number of graphs, explanatory text)."**

Is it clear to you what kind of responses could be given here?
Interviewer fill out here

- **"Other comments."**

Is it clear to you what kind of responses could be given here?
Interviewer fill out here

I would now like to continue with the layout of the questionnaire.

- Note any potential changes that are indicated by the responses.

What was the most difficult part to navigate through?
Interviewer fill out here

For which questions did you have to use the scroll bar?
Interviewer fill out here

For peer review only

If you now think about the questionnaire as a whole,

What is your overall impression?
Interviewer fill out here

After reading these questions – what would you say is the purpose of this survey?
Interviewer fill out here

Are there any questions that are *not* relevant?
Interviewer fill out here

Are there any questions that should be phrased differently?
Interviewer fill out here

Is any type of question missing?
Interviewer fill out here

What about the length of the survey?
Interviewer fill out here

- **QUESTIONNAIRE 2**

The second and last questionnaire that I want you to go through contains some other questions that we want to ask when evaluating the project and the reports on prescription patterns. It contains 7 questions. I will now email you the link to this questionnaire.

- Email the link to pilot questionnaire 2.

I would like to ask you to now fill out the second questionnaire.

- Respondent fills out the questionnaire.

Again we will now go through each question one at a time.

- **"1. In the past three months, did you ever think about your previous OPEN Stewardship report when making an antibiotic prescribing decision?"**

Can you please describe why you gave this response?
Interviewer fill out here

For peer review only

- **“2. In general, how useful do you think the OPEN Stewardship report would be to other prescribers?”**

Can you please describe why you gave this response?
Interviewer fill out here

Is it clear to you what the various responses mean?
Interviewer fill out here

- **“3. Hypothetically, how often do you think it would be appropriate to receive OPEN Stewardship reports?”**

Can you please describe why you gave this response?
Interviewer fill out here

Is it clear to you what the various responses mean?
Interviewer fill out here

- **“4. Does the pdf format of the OPEN Stewardship report work well for you?”**

Can you please describe why you gave this response?
Interviewer fill out here

- **“5. Would a central repository of local treatment guidelines be useful for your practice?”**

What does this question mean, can you describe it in other words?
Interviewer fill out here

- **“6. Do you think it would be useful to have access to a personalized web-portal where you could browse your own and local patterns of antibiotic use, as well as best practice guidelines?”**

What does this question mean, can you describe it in other words?
Interviewer fill out here

For peer review only

- **“7. Would you be willing to participate in a follow-up interview where we would ask in more detail about your experiences with the OPEN Stewardship reports?”**

Can you please describe why you gave this response?
Interviewer fill out here

- **END OF INTERVIEW**

- Note which kind of device and browser the respondent uses.

Interviewer fill out here

We have now finished. On behalf of me and all my colleagues in the project, I would like to thank you so much for taking the time to help us to improve the evaluation. It has been most useful for our work. If there is anything that you come to think of afterwards, do not hesitate to contact me.

After the interview

Email the filled out templates (in English in case you have used another language for your notes) to the Public Health Agency of Sweden who will compile from all sites. Make sure that the file names are: COUNTRY_SECTOR_ABBREVIATED-NAME-OF-RESPONDENT.

For peer review only

BMJ Open

Evaluation of an automated feedback intervention to improve antimicrobial prescribing among primary care physicians (OPEN Stewardship): Protocol for an interrupted time-series and usability analysis in Ontario, Canada and Southern Israel

Journal:	*BMJ Open*
Manuscript ID	bmjopen-2020-039810.R2
Article Type:	Protocol
Date Submitted by the Author:	09-Nov-2020
Complete List of Authors:	Soucy, Jean-Paul; University of Toronto Dalla Lana School of Public Health, Division of Epidemiology<br>Low, Marcelo; Clalit Health Services<br>Acharya, Kamal; University of Guelph Ontario Veterinary College, Department of Population Medicine<br>Ellen, Moriah; University of the Negev Guilford Glazer Faculty of Business and Management, Department of Health Services Management<br>Hulth, Anette; Folkhalsomyndigheten<br>Löfmark, Sonja; Folkhalsomyndigheten<br>Garber, Gary; Public Health Ontario, Infection Prevention and Control; University of Ottawa Faculty of Medicine, Medicine/infectious diseases<br>Watson, William; University of Toronto; St Michael's Hospital<br>Moran-Gilad, Jacob ; Ben-Gurion University of the Negev Faculty of Health Sciences<br>Fisman, David N.; University of Toronto Dalla Lana School of Public Health, Division of Epidemiology<br>MacFadden, Derek; Ottawa Hospital Research Institute, Infectious Diseases
**Primary Subject Heading**:	Public health
Secondary Subject Heading:	General practice / Family practice, Infectious diseases
Keywords:	INFECTIOUS DISEASES, Public health < INFECTIOUS DISEASES, Epidemiology < INFECTIOUS DISEASES, PRIMARY CARE

SCHOLARONE™
Manuscripts

I, the Submitting Author has the right to grant and does grant on behalf of all authors of the Work (as defined in the below author licence), an exclusive licence and/or a non-exclusive licence for contributions from authors who are: i) UK Crown employees; ii) where BMJ has agreed a CC-BY licence shall apply, and/or iii) in accordance with the terms applicable for US Federal Government officers or employees acting as part of their official duties; on a worldwide, perpetual, irrevocable, royalty-free basis to BMJ Publishing Group Ltd ("BMJ") its licensees and where the relevant Journal is co-owned by BMJ to the co-owners of the Journal, to publish the Work in this journal and any other BMJ products and to exploit all rights, as set out in our licence.

The Submitting Author accepts and understands that any supply made under these terms is made by BMJ to the Submitting Author unless you are acting as an employee on behalf of your employer or a postgraduate student of an affiliated institution which is paying any applicable article publishing charge ("APC") for Open Access articles. Where the Submitting Author wishes to make the Work available on an Open Access basis (and intends to pay the relevant APC), the terms of reuse of such Open Access shall be governed by a Creative Commons licence – details of these licences and which Creative Commons licence will apply to this Work are set out in our licence referred to above.

Other than as permitted in any relevant BMJ Author's Self Archiving Policies, I confirm this Work has not been accepted for publication elsewhere, is not being considered for publication elsewhere and does not duplicate material already published. I confirm all authors consent to publication of this Work and authorise the granting of this licence.

For peer review only

Evaluation of an automated feedback intervention to improve antimicrobial prescribing among primary care physicians (OPEN Stewardship): Protocol for an interrupted time-series and usability analysis in Ontario, Canada and Southern Israel

Jean-Paul R. Soucy[1], Marcelo Low[2, 3], Kamal R. Acharya[4], Moriah Ellen[5, 6, 7], Anette Hulth[8,], Sonja Löfmark[8], Gary E. Garber[9, 10, 11], William Watson[12, 13], Jacob Moran-Gilad[14], David N. Fisman[1]*, Derek R. MacFadden[15]*

* co-senior authors

[1] Division of Epidemiology, Dalla Lana School of Public Health, University of Toronto, Toronto, Canada
[2] Clalit Health Services, Chief Physician's Office, Tel Aviv, Israel
[3] School of Public Health, University of Haifa, Haifa, Israel
[4] Department of Population Medicine, University of Guelph, Guelph, Canada
[5] Department of Health Systems Management, Guilford Glazer Faculty of Business and Management and Faculty of Health Sciences, Ben-Gurion University of the Negev, Beer Sheva, Israel
[6] McMaster Health Forum, McMaster University, Hamilton, Canada
[7] Institute for Health Policy, Management and Evaluation, University of Toronto, Toronto, Canada
[8] Public Health Agency of Sweden, Stockholm, Sweden
[9] Infection Prevention and Control, Public Health Ontario, Toronto, Canada
[10] Department of Medicine, University of Toronto, Toronto, Canada
[11] Department of Medicine, University of Ottawa, Ottawa, Canada
[12] St Michael's Hospital, Toronto, Canada
[13] Department of Family & Community Medicine, University of Toronto, Canada
[14] School of Public Health, Faculty of Health Sciences, Ben-Gurion University of the Negev, Beer Sheva, Israel
[15] Ottawa Hospital Research Institute, Ottawa, Canada

Corresponding author: Derek R. MacFadden (dmacfadden@toh.ca): Ottawa Hospital Research Institute, 1053 Carling Ave, Ottawa, ON, Canada K1Y 4E9

Word count: 3,332

Keywords: antimicrobial stewardship, antimicrobial resistance, antimicrobial prescribing, primary care, interrupted time-series analysis

For peer review only

ABSTRACT

Introduction

Antimicrobial resistance undermines our ability to treat bacterial infections, leading to longer hospital stays, increased morbidity and mortality, and a mounting burden to the healthcare system. Antimicrobial stewardship is increasingly important to safeguard the efficacy of existing drugs, as few new drugs are in the developmental pipeline. While significant progress has been made with respect to stewardship in hospitals, relatively little progress has been made in the primary care setting, where the majority of antimicrobials are prescribed. OPEN Stewardship is an international collaboration to develop an automated feedback platform to improve responsible antimicrobial prescribing among primary care physicians and capable of being deployed across heterogeneous healthcare settings. We describe the protocol for an evaluation of this automated feedback intervention with two main objectives: assessing changes in antimicrobial prescribing among participating physicians and determining the usability and usefulness of the reports.

Methods and analysis

A non-randomized evaluation of the automated feedback intervention (OPEN Stewardship) will be conducted among approximately 150 primary care physicians recruited from Ontario, Canada and Southern Israel, based on a series of targeted stewardship messages sent using the platform. Using a controlled interrupted time-series analysis and multilevel negative binomial modelling, we will compare the antimicrobial prescribing rates of participants before and after the intervention, and also to the prescribing rates of non-participants (from the same healthcare network) during the same period. We will examine outcomes targeted by the stewardship messages, including prescribing for antimicrobials with duration longer than seven days and prescribing for indications where antimicrobials are typically unnecessary. Participants will also complete a series of surveys to determine the usability and usefulness of the stewardship reports.

Ethics and dissemination

All sites have obtained ethics committee approval to recruit providers and access anonymized prescribing data. Dissemination will occur through open-access publication, stakeholder networks, and national/international meetings.

STRENGTH AND LIMITATIONS OF THIS STUDY

- Multinational pragmatic study
- Large number of providers studied with extended follow-up
- Comprehensive evaluation including pre-post design and concurrent controls
- Scalable and open concept with potential for broad generalizability to various care settings
- Differences in recruitment strategy for intervention group across the two study sites may result in heterogeneity of intervention effect

For peer review only

INTRODUCTION

The World Health Organization has called antimicrobial resistance “one of the biggest threats to global health, food security, and development today.” [1] Antimicrobial resistance undermines our ability to treat bacterial infections, leading to longer hospital stays, increased complications, higher mortality, and a mounting resource burden on healthcare systems globally [1–5]. It has been projected that resistant microbes could contribute to up to 10 million deaths per year by 2050, greater than the current annual burden of cancer deaths. Additionally, a cumulative loss in global production of trillions of USD in the same time frame is possible [6], hindering efforts to eliminate poverty [7]. Despite a widespread recognition of antimicrobial use as the leading driver of resistance, inappropriate prescribing remains common [8–10]. Given the paucity of new antimicrobials in the drug development pipeline [11], it is critical that we act to safeguard the effectiveness of existing drugs by reducing inappropriate antimicrobial prescribing.

Antimicrobial stewardship refers to the effort to promote the responsible use of antimicrobials and to ensure that patients receive the best choice, duration, and route of antimicrobial treatment, while minimizing potential side effects and the dissemination of antimicrobial resistance [12]. While progress has been made in advancing antimicrobial stewardship in hospitals, leading to significant cost savings and improved patient outcomes [13,14], progress in the primary care setting has been slower [15–17]. This is troubling, given that most antimicrobials are prescribed in the outpatient setting, rather than in hospitals (e.g., over 90% in Canada) [18,19]. Supporting general and family practitioners with prescribing is especially important, since they account for the majority of antimicrobials dispensed for human use in Canada [19]. The United States Centers for Disease Control and Prevention have issued a checklist identifying the key elements to a successful antimicrobial stewardship program in an outpatient setting, highlighting easy access to evidence-based prescribing guidelines and the technical capacity to track, evaluate, and report on antimicrobial prescribing [20]. An online, automated feedback system facilitating these elements would be a major asset to advancing stewardship in primary care.

OPEN Stewardship is an international collaboration with the goal of developing an open, web-based platform to improve antimicrobial prescribing among primary care physicians. This platform integrates with local antimicrobial prescribing data to provide participating physicians with regular, automated feedback (identified by a recent Cochrane review as an effective technique to enable appropriate antimicrobial prescribing in the hospital setting [14]) and connects users with locally relevant prescribing guidelines. The development of a standardized, accessible stewardship platform addresses a key gap for stewardship interventions in the primary care setting, namely the difficulty of expanding across heterogeneous, resource-constrained healthcare systems. As part of a One Health approach recognizing the interconnectedness of human and animal health [21], the platform is also being developed for use in veterinary care, a sector where antimicrobial stewardship has received less attention [22]. A study parallel to the present study will be occurring among veterinary care providers.

STUDY APPROACH AND OBJECTIVES

In this study, we will enrol primary care physicians on a voluntary basis from practices in two study sites: Ontario, Canada and Southern Israel. Non-participating physicians (from the same healthcare network) will be available as controls. The intervention group will receive three personalized electronic reports sent over the span of six months, benchmarking their prescribing rate to those of their peers and attaching locally relevant prescribing guidelines for specific indications. The messaging will target antimicrobial prescribing longer than seven days (few conditions require longer than this [23]), as well as two indications in particular: acute sinusitis and viral respiratory conditions. These common

For peer review only

infections are a promising target for stewardship [8,20,24,25]. In the US, acute respiratory conditions as a whole account for close to half of antimicrobials prescribed and a great majority of those prescribed inappropriately in the ambulatory care setting [8].

The aim of this project is to assess the impact of an automated feedback intervention on antimicrobial prescribing in primary care physicians. Specifically, for a cohort of primary care physicians in Ontario, Canada and Southern Israel, we will determine the impact of the stewardship intervention on:

1. The overall antimicrobial prescribing rate in the period following the intervention compared to the period preceding it and to a cohort of primary care physicians who did not receive the intervention.

2. The prescribing rate for antimicrobials with duration longer than seven days in the period following the intervention compared to the period preceding it and to a cohort of primary care physicians who did not receive the intervention.

3. The antimicrobial prescribing rate for viral respiratory conditions in the period following the intervention compared to the period preceding it and to a cohort of primary care physicians who did not receive the intervention.

4. The antimicrobial prescribing rate for acute sinusitis in the period following the intervention compared to the period preceding it and to a cohort of primary care physicians who did not receive the intervention.

We will also assess:

5. The usability and usefulness of each component of the antimicrobial stewardship reports, as well as the usefulness of the reports overall.

METHODS AND ANALYSIS

Objectives 1–4: Non-randomized evaluation of intervention with interrupted time-series analysis

Study design

Primary care physicians (providers) from two study sites will be voluntarily enrolled to participate in an antimicrobial stewardship intervention. The providers' rates of antimicrobial prescribing after the intervention will be compared to their rates prior to having received the reports, as well as the prescribing rates of their peers in the same network who did not participate in the intervention. This study design is know as a controlled interrupted time-series [26]. Our study design addresses the limitations of some previous studies of stewardship interventions, including small sample sizes, single-centre design, a lack of external controls, and short follow-up [16].

Participants

Approximately 100–150 general practitioners will be enrolled from two study sites: Ontario, Canada (50) and Southern Israel (50–100). Participants will be drawn from the University of Toronto Practice-Based Research Network (UTOPIAN) and Clalit Health Services (CHS), respectively. UTOPIAN is "one of the largest and most representative primary care research networks in North America" [27], including data for over 400 physicians with an average practice size of 1,018 patients [28]. This

For peer review only

includes family medicine practices and primary care practices in university-affiliated hospitals. CHS is the largest healthcare maintenance organization in Southern Israel [29] and covers nearly 80% of the population in Southern Israel through a network family medicine practices. Existing data storage and sharing facilities within both of these networks allow access to de-identified patient data for research in the primary care setting.

In Ontario, primary care physicians will be voluntarily enrolled, provided they worked for at least 12 months prior the intervention, expect to continue working for the 12 months following the start of the intervention, and are not actively participating in another stewardship intervention. Providers will be approached for enrolment via email or in-person presentations to groups of providers. Participants will begin the intervention in at least two waves in different months. In Southern Israel, half of the approximately 400 eligible physicians (average number of patients: 1,482) will be randomized (using random assignment in an Excel spreadsheet by the study coordinator) to be approached (via a letter from the Chief Physician, followed by contact by an interviewer) for voluntary enrolment in the study. Anonymized prescribing data of non-participating physicians within UTOPIAN and CHS will be available as an external control group (approximately 350 for UTOPIAN and 250–300 for CHS, with an expected 25–50% response rate for physicians approached to enrol).

It is possible that physicians who voluntarily enrol in the antimicrobial stewardship intervention will be more engaged with stewardship prior to the intervention, and thus may have less to gain from a stewardship intervention than less-engaged physicians. Differences in the recruitment strategy between sites may result in a heterogeneity in the observed effect, which will be accounted for in the analysis.

Intervention

The intervention will consist of three personalized reports generated by the OPEN Stewardship platform and emailed to participants in the intervention group. The first report will be sent at the beginning of the intervention period (t_0) and will consist of a welcome email followed by a benchmark of the provider's overall antimicrobial prescribing rate relative to the average and 25th percentile of participants in the same network (UTOPIAN or CHS) during the previous year (2019). This report will also include guidelines highlighting that most conditions do not require more than 7 days of antimicrobials and a benchmark of the provider's proportion of antimicrobial prescriptions with duration longer than 7 days relative to the average and 25th percentile of participants in the same network during the previous year. We chose to benchmark against other participants only (excluding non-participants) due to the expectation that those self-selecting into a stewardship intervention may already have lower rates of prescribing.

The two subsequent reports will be sent 3 (t_3) and 6 (t_6) months after the first report. These reports will target viral respiratory conditions (as defined by the primary ICD-9 code associated with the visit, see table S1) and acute sinusitis, respectively, and will consist of a benchmark of the provider's prescribing for the specified indication as well as site-specific best practice guidelines for the specified indication. The study concludes one year after the first report is sent (t_{12}). The composition of each report is summarized in table 1. An example figure is shown in figure 1.

Outcomes

Each of the 4 objectives corresponds to an outcome, measured for each provider as the monthly prescribing rate per 100 visits for a particular indication. This prescribing rate is calculated as the

For peer review only - http://bmjopen.bmj.com/site/about/guidelines.xhtml

number of prescriptions for a particular indication divided by the number of patient visits for that indication in a month, multiplied by 100.

1. Overall prescribing rate for antimicrobials.
2. The prescribing rate for antimicrobials lasting longer than seven days in duration.
3. The prescribing rate for visits classified as being for a viral respiratory condition (see table S1 for ICD-9 codes).
4. The prescribing rate for acute sinusitis visits.

While overall antimicrobial prescribing is a proxy for our desired measure, which is appropriate antimicrobial prescribing, the microbiological data do not exist to validate the appropriateness of each individual prescription. Nonetheless, given the ubiquity of inappropriate prescribing, particularly for acute respiratory tract infections, any reduction in prescribing as a result of the intervention is likely to be positive [8,30,31].

Covariates

The analysis will include potential predictors of a provider's baseline prescribing rate, as well as seasonality (table 2). These include a provider's age, sex, and employment status (full-time or part-time) [10] and monthly practice patient composition (by age and sex) [19,32]. We will also use nested random intercepts for provider, practice group (clinic), and study site, representing within-group prescribing norms and a provider's baseline willingness to prescribe after accounting for other predictors, as well as heterogeneity between study sites. Seasonality will be accounted for using a categorical variable for each month compared to January. An interaction term with study site will be included for each covariate to allow for differences in seasonal and demographic prescribing patterns between the two countries in our study.

Statistical analysis

We will perform a multilevel, controlled interrupted time-series analysis with the provider as the unit of analysis [33]. A negative binomial model (to allow for overdispersion of the outcome) will be used with prescribing rates as the outcome. Each provider in the intervention group will contribute 24 observations: a minimum of 12 months in the pre-intervention period (t_{-12} to t_0) and up to 12 months in the post-intervention period, depending on the objective. The pre-intervention and post-intervention periods of each outcome are defined by the time when the relevant report was delivered. The first report (t_0) corresponds to objectives 1 and 2, the second report (t_3) to objective 3, and the third report (t_6) to objective 4. The pre-intervention and post-intervention periods of each objective are summarized in table 3. Providers in the external control group will contribute 24 observations in the control period. The timeline of the study for each objective is shown in figure 2.

The intervention effect will be modelled as a level change and/or change in slope for the prescribing rate at the beginning of the post-intervention period [26]. An interaction term with study site will be included to allow for differences in the intervention effect between Ontario and Southern Israel. A key assumption of interrupted time-series analysis is that the pre-intervention time trend is assumed to be linear, which must be evaluated through visualization [34]. Otherwise, non-linear terms may be considered. Model fit can be assessed by comparing models containing intervention parameters to a null model not containing intervention parameters using a statistic such as Akaike information criterion (AIC).

For peer review only

Autocorrelation in each provider's time-series should be accounted for by the random intercept (provider nested within practice group nested within study site) and by controlling for seasonality (categorical variable for month) [26]. In case autocorrelation is present in the residual distribution, an autoregressive (AR1) covariance structure will be considered.

Stewardship interventions often result in a reversion to baseline behaviour after the intervention is discontinued [35,36]. To examine the durability of the intervention effect, we will contrast the coefficients estimated from models including 1) all months in the post-intervention period, 2) the first 3 months in the post-intervention period, 3) the first 6 months in the post-intervention period. Finally, since the first report at t_0 (highlighting overall prescribing) is likely to affect objectives targeted by later reports (at t_3 or t_6), for objectives 3 and 4, we will consider t_0 as an alternative start time of the post-intervention period.

As the COVID-19 pandemic represents a major disruption of primary care, its effect on prescribing will be modelled as one or more separate "intervention points" in the time-series analysis.

Sensitivity analyses

Alternative parameterizations of the study outcomes will be considered. For the prescribing rate of antimicrobials with courses longer than seven days (objective 2), we will consider total days of therapy (with the number of prescriptions as the offset) as an alternative outcome using a zero-truncated negative binomial model. There may be issues with how indications are coded in the electronic medical record, whereas the type of antimicrobial prescribed is very likely to be accurate. A sensitivity analysis will be performed using the prescribing rates (for all visits) for antimicrobials most often prescribed for viral respiratory conditions (e.g., amoxicillin, amoxicillin/clavulanic acid, azithromycin [37]) as the outcome, rather than the indication-specific prescribing rate of antimicrobials for viral respiratory conditions. Since acute respiratory tract infections make up a large proportion of total antimicrobial prescribing, the signal from the intervention may be evident using these outcomes as well.

To ascertain whether there could be negative effects of the stewardship intervention, we will evaluate (1) the frequency of bacterial pneumonia and pyelonephritis diagnoses and (2) the number of hospital admissions (including those related to infection) in the pre-intervention and post-intervention periods. Admissions data are only available for CHS (Israel), and thus will only be evaluated at this site only.

Including patient composition as predictors (e.g., percentage of patients 65 and older, percentage of female patients) may be insufficient to capture differences in prescribing occurring between strata of age and sex. A sensitivity analysis will be performed where the outcome will be stratified by age group and sex and modelled separately. Although stratification increases the noise in each individual time-series, these models may reveal differences in the effectiveness of the intervention across sex/age groups, which have different prescribing patterns.

As the offer to enrol in the study is randomized at one study site (Southern Israel), an intention-to-treat analysis will be performed as a supplementary analysis at this site only. The characteristics and prescribing patterns of responders and non-responders among those offered enrolment will also be compared.

Sample size

For peer review only

We provide an approximate sample size calculation for a difference in antimicrobial prescribing between two groups (intervention and control) in a Poisson framework using the method described by Whitehead [38] and implemented in an online calculator (http://www.obg.cuhk.edu.hk/ResearchSupport/StatTools/SSiz2Counts_Tab.php). In Fleming-Dutra's [8] study of outpatient prescribing, 12.6% of visits resulted in an antimicrobial prescription. Since most visits would only results in a single antimicrobial prescription, we take this as the baseline prescribing rate (0.126/visit). The American Academy of Family Physicians estimated in 2012 that their members had an average of 92 patient encounters per week (368 per month) [39]. We define a 15% reduction in prescribing rate as clinically meaningful (0.107/visit). To achieve a power of 90% (with $\alpha = 0.05$, one-tail), we would require approximately 15 physicians in each group to be observed for 1 month each, which is much smaller than our expected sample size at each study site. While the clustering and likely over-dispersion of prescribing rates in our sample would decrease our power, the fact that we will have 24 months of observations for each provider (rather than 1 month), as well as pre-intervention and post-intervention observations, greatly enhances the power of our study to detect an intervention effect.

Objective 5: Survey of usability among participating providers

Study design

Physicians enrolled in the antimicrobial stewardship intervention will receive three short surveys to assess the usability of the reports received and to provide feedback on the intervention.

Surveys

Surveys will be delivered by email to participants in the intervention group 3 days after the initial receipt of each report using Qualtrics (Qualtrics, Provo, UT) in Southern Israel and RedCAP [40] survey software in Ontario (due to differences in institutional policies regarding data collection). The report will be re-sent with the survey and the recipient will be given 7 days to respond. If the recipient fails to respond within this allotted time, the survey and report are re-sent again, giving the recipient an additional 7 days to respond. The first two surveys focus on the usability and usefulness of the emailed reports, with additional space for free text feedback. The final report adds additional questions to assess how often they would prefer to receive reports and whether particular additional features would be of use to him or her. Survey questions are given in English in Appendix 1; translated questions in Hebrew are given in Appendix 2. Questions were developed in accordance with experts at the Public Health Agency of Sweden. Questions were pilot tested and revised according to the protocol and interviewer guide in Appendix 3.

Statistical analysis

Participants' attitudes regarding the overall usefulness of the intervention and the informativeness of the figure included in each intervention (table 1) will be assessed in each survey and compared longitudinally with a multilevel linear model. Responses to other questions may be analyzed using ordinal regression and/or multinomial regression.

ETHICS AND DISSEMINATION

The following ethics committees reviewed and approved this study: University of Toronto REB (Canada), St. Michael's Hospital REB (Canada), Toronto East Health Network REB (Canada), Clalit REB (Israel). The veterinary study was further reviewed and approved by: University of Guelph REB

For peer review only

(Canada) and Internal Research Review Committee of the Koret School of Veterinary Medicine – Veterinary Teaching Hospital, Hebrew University (Israel).

All patient-level prescribing data will be anonymized prior to receipt, available only as the rate of antimicrobial prescribing per visit, stratified by month and reason for visit (and potentially sex and/or broad age category). Study data will be stored on a secure server and accessible only to study analysts. Study participants from Ontario will be compensated with a gift card. Study participants from Israel will be encouraged to participate by a letter from the Chief Physician's Office.

The OPEN Stewardship project includes collaborators affiliated with hospitals, universities, and public health authorities in four countries. In addition to dissemination through stakeholder networks, the results of this study will be published in an open-access journal and presented at national and/or international meetings.

PATIENT AND PUBLIC INVOLVEMENT

Primary care physicians were involved in the conception and design of this study, as well as the study surveys. They were also consulted to assess the appropriate time burden and adequate compensation for participants in the study.

For peer review only

REFERENCES

1 World Health Organization. Antibiotic resistance. 2019.http://www.who.int/mediacentre/factsheets/antibiotic-resistance/en/ (accessed 1 Jan 2019).

2 The Review on Antimicrobial Resistance. Tackling Drug-resistant Infections Globally: Final Report and Recommendations. 2016.https://amr-review.org/sites/default/files/160518_Final%20paper_with%20cover.pdf (accessed 27 May 2018).

3 World Health Organization. GLASS | Global antimicrobial resistance surveillance system (GLASS) report. Geneva: 2017.

4 Interagency Coordination Group on Antimicrobial Resistance. No Time to Wait: Securing the future from drug-resistant infections. Interagency Coordination Group on Antimicrobial Resistance 2019. https://www.who.int/antimicrobial-resistance/interagency-coordination-group/IACG_final_report_EN.pdf (accessed 18 Jul 2019).

5 Roberts RR, Hota B, Ahmad I, *et al.* Hospital and Societal Costs of Antimicrobial-Resistant Infections in a Chicago Teaching Hospital: Implications for Antibiotic Stewardship. *Clin Infect Dis* 2009;**49**:1175–84. doi:10.1086/605630

6 The Review on Antimicrobial Resistance. Antimicrobial Resistance: Tackling a Crisis For the Health and Wealth Of Nations. 2014.

7 Ahmed SA, Bariş E, Go DS, *et al.* Assessing the Global Economic and Poverty Effects of Antimicrobial Resistance. 2017.

8 Fleming-Dutra KE, Hersh AL, Shapiro DJ, *et al.* Prevalence of inappropriate antibiotic prescriptions among US ambulatory care visits, 2010-2011. *JAMA* 2016;**315**:1864–73.

9 Shively NR, Buehrle DJ, Clancy CJ, *et al.* Prevalence of Inappropriate Antibiotic Prescribing in Primary Care Clinics within a Veterans Affairs Health Care System. *Antimicrob Agents Chemother* 2018;**62**:e00337-18.

10 Cadieux G, Tamblyn R, Dauphinee D, *et al.* Predictors of inappropriate antibiotic prescribing among primary care physicians. *Can Med Assoc J* 2007;**177**:877–83.

11 Renwick MJ, Simpkin V, Mossialos E. Targeting innovation in antibiotic drug discovery and development: the need for a one health - one Europe - one world framework. 2016.http://www.ncbi.nlm.nih.gov/books/NBK447337/ (accessed 18 Feb 2020).

12 Dyar OJ, Huttner B, Schouten J, *et al.* What is antimicrobial stewardship? *Clin Microbiol Infect* 2017;**23**:793–8.

13 Nathwani D, Varghese D, Stephens J, *et al.* Value of hospital antimicrobial stewardship programs [ASPs]: a systematic review. *Antimicrob Resist Infect Control* 2019;**8**.

14 Davey P, Marwick CA, Scott CL, *et al.* Interventions to improve antibiotic prescribing practices for hospital inpatients. *Cochrane Database Syst Rev* Published Online First: 9 February 2017.

For peer review only

15 Bal AM, Gould IM. Antibiotic stewardship: Overcoming implementation barriers. *Curr Opin Infect Dis* 2011;**24**:357–62.

16 Allerberger F, Gareis R, Jindrák V, *et al.* Antibiotic stewardship implementation in the EU: the way forward. *Expert Rev Anti Infect Ther* 2009;**7**:1175–83.

17 Langford BJ, Schwartz KL, Garber GE. Antimicrobial stewardship by family physicians. *Can Fam Physician* 2018;**64**:168–9.

18 Drekonja D, Filice G, Greer N, *et al. Antimicrobial Stewardship Programs in Outpatient Settings: A Systematic Review*. Washington DC: Department of Veterans Affairs 2014. http://www.ncbi.nlm.nih.gov/books/NBK274571/ (accessed 19 Jul 2019).

19 Public Health Agency of Canada. Canadian Antimicrobial Resistance Surveillance System – 2017 Report. 2017.

20 Sanchez GV, Fleming-Dutra KE, Roberts RM, *et al.* Core Elements of Outpatient Antibiotic Stewardship. *MMWR Recomm Rep* 2016;**65**:1–12.

21 McEwen SA, Collignon PJ. Antimicrobial Resistance: a One Health Perspective. In: Schwarz S, Cavaco LM, Shen J, eds. *Antimicrobial Resistance in Bacteria from Livestock and Companion Animals*. Washington DC: ASM Press 2018. 521–47.

22 Lloyd DH, Page SW. Antimicrobial Stewardship in Veterinary Medicine. In: Schwarz S, Cavaco LM, Shen J, eds. *Antimicrobial Resistance in Bacteria from Livestock and Companion Animals*. Washington DC: ASM Press 2018. 675–97.

23 Pouwels KB, Hopkins S, Llewelyn MJ, *et al.* Duration of antibiotic treatment for common infections in English primary care: cross sectional analysis and comparison with guidelines. *BMJ* 2019;**364**:l440.

24 Smith SM, Fahey T, Smucny J, *et al.* Antibiotics for acute bronchitis. *Cochrane Database Syst Rev* Published Online First: 19 June 2017.

25 Little P, Stuart B, Francis N, *et al.* Effects of internet-based training on antibiotic prescribing rates for acute respiratory-tract infections: a multinational, cluster, randomised, factorial, controlled trial. *The Lancet* 2013;**382**:1175–82.

26 Bernal JL, Cummins S, Gasparrini A. Interrupted time series regression for the evaluation of public health interventions: a tutorial. *Int J Epidemiol* 2017;**46**:348–55.

27 University of Toronto Practice-Based Research Network. About UTOPIAN. 2017.http://www.dfcm.utoronto.ca/about-utopian (accessed 17 Feb 2019).

28 University of Toronto Practice-Based Research Network. UTOPIAN Data Safe Haven. 2019.http://www.dfcm.utoronto.ca/utopian-data-safe-haven (accessed 4 Sep 2019).

29 Clalit. The Story of Clalit Health Services. 2010.http://www.clalit-global.co.il/en/_the_story_of_clalit_health_services.html (accessed 21 Jul 2019).

For peer review only

30 Meeker D, Linder JA, Fox CR, *et al.* Effect of Behavioral Interventions on Inappropriate Antibiotic Prescribing Among Primary Care Practices: A Randomized Clinical Trial. *JAMA* 2016;**315**:562.

31 Groth A, Enoksson F, Hermansson A, *et al.* Acute mastoiditis in children in Sweden 1993–2007—No increase after new guidelines. *Int J Pediatr Otorhinolaryngol* 2011;**75**:1496–501.

32 Dosh SA, Hickner JM, Mainous AGI, *et al.* Predictors of Antibiotic Prescribing for Nonspecific Upper Respiratory Infections, Acute Bronchitis, and Acute Sinusitis. *J Fam Pract* 2000;**49**:409–15.

33 Taljaard M, McKenzie JE, Ramsay CR, *et al.* The use of segmented regression in analysing interrupted time series studies: an example in pre-hospital ambulance care. *Implement Sci* 2014;**9**.

34 Kontopantelis E, Doran T, Springate DA, *et al.* Regression based quasi-experimental approach when randomisation is not an option: interrupted time series analysis. *BMJ* 2015;**350**.

35 Arnold SR, Straus SE. Interventions to improve antibiotic prescribing practices in ambulatory care. *Cochrane Database Syst Rev* Published Online First: 2005.

36 Gerber JS, Prasad PA, Fiks AG, *et al.* Durability of Benefits of an Outpatient Antimicrobial Stewardship Intervention After Discontinuation of Audit and Feedback. *JAMA* 2014;**312**:2569–70.

37 Ebell MH, Radke T. Antibiotic Use for Viral Acute Respiratory Tract Infections Remains Common. *Am J Manag Care* 2015;**21**:9.

38 Whitehead J. *The design and analysis of sequential clinical trials*. Rev. 2nd ed. Chichester, UK: John Wiley & Sons 1997.

39 American Academy of Family Physicians. AAFP Member Survey Provides Valuable Perspective. 2017.https://www.aafp.org/news/blogs/inthetrenches/entry/20170801ITT_Survey.html (accessed 27 Feb 2020).

40 Harris PA, Taylor R, Thielke R, *et al.* Research electronic data capture (REDCap)—A metadata-driven methodology and workflow process for providing translational research informatics support. *J Biomed Inform* 2009;**42**:377–81.

For peer review only

AUTHORS' CONTRIBUTIONS

J-PRS, ML, KRA, ME, AH, SL, GEG, WW, JM-G, DNF, and DRM conceived of the study and the analysis. J-PRS wrote the initial manuscript and will conduct the data analysis. All authors contributed to and approved the final manuscript.

FUNDING STATEMENT

This work was supported by the European Commission's Joint Programming Initiative on Antimicrobial Resistance (Swedish Research Council: 2017-05972, Canadian Institutes of Health Research: AMR - 155212, Israeli Ministry of Health: 8762491).

COMPETING INTERESTS STATEMENT

The authors declare no conflicts of interest.

For peer review only

Tables

Table 1. Composition of the feedback reports (intervention) at each time point.

Feedback report	Content of report	Benchmarking figure	Guidelines
Report at t_0	1. Antimicrobial prescribing rate/100 visits in the previous year for all visits.<br>2. Proportion of antimicrobial prescriptions in the previous year with duration over 7 days.	Provider's own rate/proportion versus average rate/proportion and 25th percentile rate/proportion across all participants at the same study site.	Guidelines indicating that antimicrobial prescribing longer than 7 days is not necessary for many conditions.
Report at t_3	1. Antimicrobial prescribing rate/100 visits in the previous year for viral respiratory conditions.	Provider's own rate versus average rate and 25th percentile rate across all participants at the same study site.	Prescribing guidelines for viral respiratory conditions.
Report at t_6	1. Antimicrobial prescribing rate/100 visits in the previous year for acute sinusitis.	Provider's own rate versus average rate and 25th percentile rate across all participants at the same study site.	Prescribing guidelines for acute sinusitis.

For peer review only

Table 2. Covariates included in the interrupted time-series analysis of the OPEN Stewardship study. Excludes variables related to the effect of the COVID-19 pandemic.

Variable	Purpose	Form	Description
Number of prescriptions	Outcome	Integer	Number of antimicrobial prescriptions for given indication
Number of visits for indication	Offset	Integer	Number of patient visits for given indication
Study site	Covariate	Binary	Southern Israel versus Ontario, Canada (baseline)
Practice group	Random intercept	ID	Random intercept for practice group (nested within region)
Provider	Random intercept	ID	Random intercept for provider (nested within practice group)
Month	Covariate	Categorical	Calendar month (baseline = January)
Month × study site	Interaction	Interaction	Allows different value for coefficients for Southern Israel
Physician age	Covariate	Integer	Physician age at t_{-12}
Physician age × study site	Interaction	Interaction	Allows different value for coefficient for Southern Israel
Physician sex	Covariate	Binary	Female versus male (baseline)
Physician sex × study site	Interaction	Interaction	Allows different value for coefficient for Southern Israel
Physician employment status	Covariate	Binary	Part-time versus full-time (baseline)
Physician employment status × study site	Interaction	Interaction	Allows different value for coefficient for Southern Israel
Percent female patients	Covariate	Continuous	Percentage of visits by female patients
Percent female patients × study site	Interaction	Interaction	Allows different value for coefficient for Southern Israel
Percent patients 17 and under	Covariate	Continuous	Percentage of visits by patients 17 and younger
Percent patients 17 and under × study site	Interaction	Interaction	Allows different value for coefficient for Southern Israel
Percent patients 65 and older	Covariate	Continuous	Percentage of visits by patients 65 and older
Percent patients 65 and older × study site	Interaction	Interaction	Allows different value for coefficient for Southern Israel
Post-intervention	Intervention	Binary	1 if provider is in intervention group and intervention has occurred, 0 otherwise; level change of the outcome associated with intervention
Post-intervention × study site	Interaction	Interaction	Allows different value for level change of the outcome for Southern Israel
Time	Trend	Integer	Time since the beginning of the study; slope of the time trend of the outcome
Time × study site	Interaction	Interaction	Allows different value for slope of the time trend of the outcome for Southern Israel
Time × post-intervention	Intervention effect	Interaction	Change in slope of time trend in the intervention group after intervention is received
Time × post-intervention × study site	Interaction	Interaction	Allows different value for change in slope of time trend in the intervention group after intervention is received for Southern Israel

For peer review only

Table 3. Timing of analyses for objective 1–4 of the study. The definitions of the pre-intervention and post-intervention periods are dependant on the delivery time of the report (intervention) related to each objective.

Objective	Outcome	Unit of analysis	Intervention time	Number of observations in intervention cohort		Total observations/ observations in control cohort
				Pre-intervention	Post-intervention	
1	Prescribing for all indications	Provider	t_0	12	12	24
2	Prescribing (for all indications) with duration longer than 7 days	Provider	t_0	12	12	24
3	Prescribing for viral respiratory conditions	Provider	t_3	15	9	24
4	Prescribing for acute sinusitis	Provider	t_6	18	6	24

For peer review only

Figure legends

Figure 1. Example of chart sent in the first feedback report, representing the provider's prescribing rate/100 visits for any indication versus the average rate and 25th percentile rate across all providers in the study from the same study site.

Figure 2. Pre-intervention and post-intervention periods for objectives 1–4 of the OPEN Stewardship study. A: Objective 1, overall prescribing (report sent at t_0). B: Objective 2, prescribing with duration longer than 7 days (report sent at t_0). C: Objective 3, prescribing for viral respiratory conditions (report sent at t_3). D: Objective 4, prescribing for acute sinusitis (report sent at t_6). t_0 represents the time of the first report (intervention); t_{-12} represents 1 year prior to the first report; t_{12} marks the conclusion of the study period (1 year after the first report).

For peer review only

Number of Prescriptions of Any Antibiotic (Per 100 Visits) for Any Indication

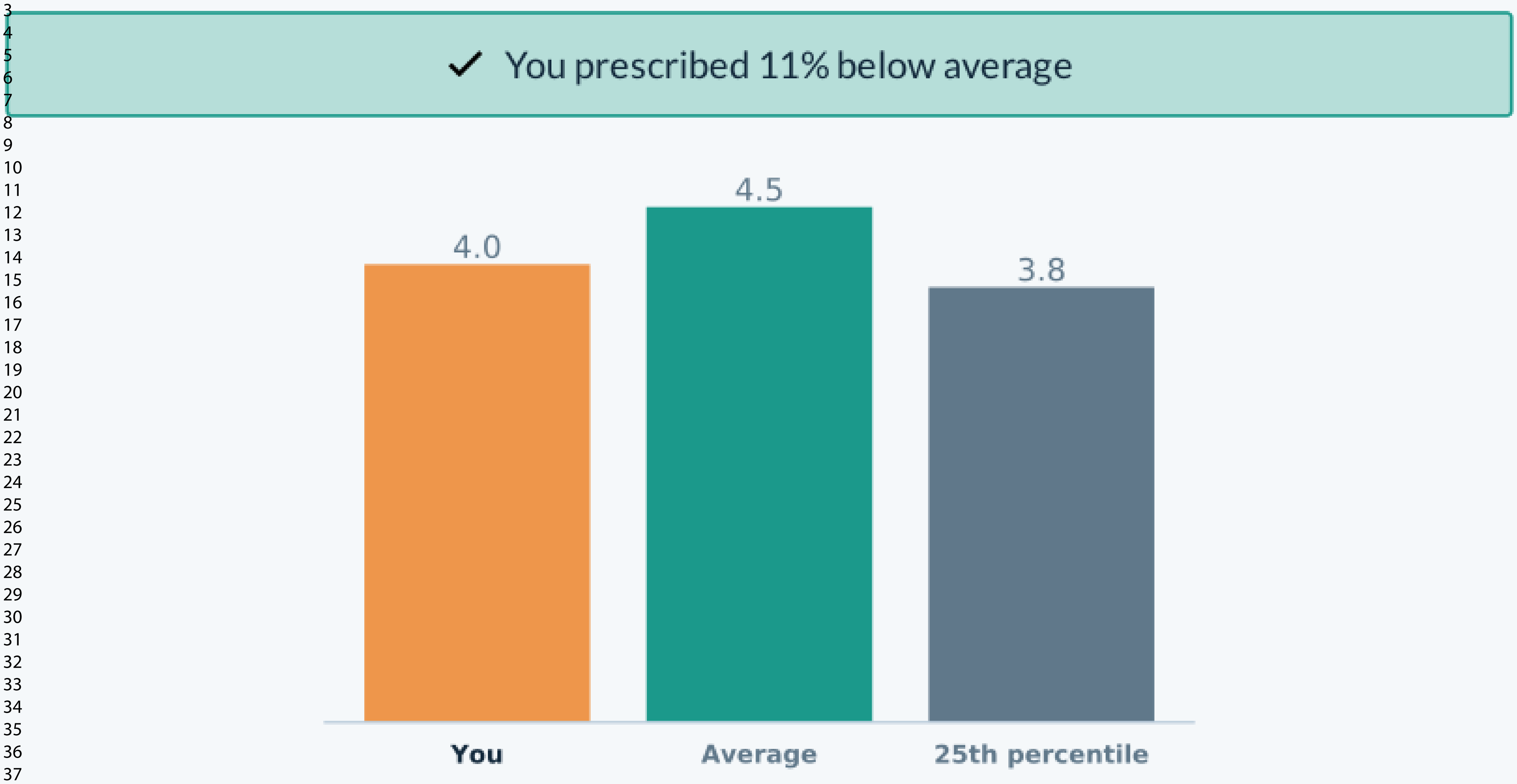

You had 5001 patient visits from Jan 01, 2019 to Dec 31, 2019 .

This figure shows how frequently you prescribed antibiotics for any indication in the previous year (2019). Your prescribing rate (prescriptions per 100 visits) is compared to the average (mean) of your colleagues and participants in Southern Ontario as well as showing your position relative to the 25th percentile. Generally, it is better to be below the average rate of prescribing and within the lowest 25th percentile.

These data are not adjusted for physician's practice characteristics, nor do they differentiate between delayed or non-filled prescriptions. These results are generated from claims data, and misclassification of the diagnosis may be present. As a result, our prescribing targets are referenced to peer benchmarks. Learn more about the data and its interpretation.

For peer review only - http://bmjopen.bmj.com/site/about/guidelines.xhtml

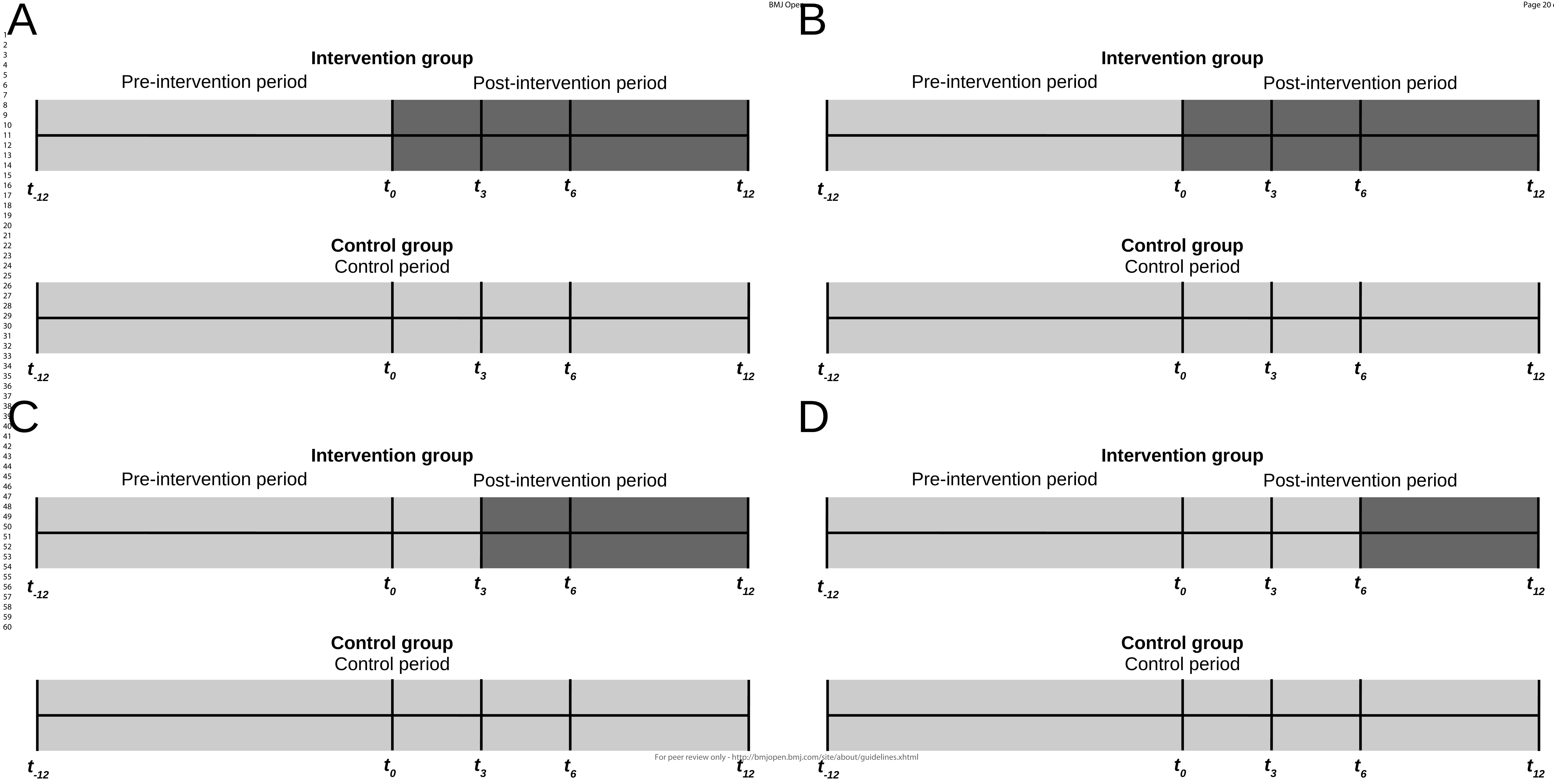

For peer review only - http://bmjopen.bmj.com/site/about/guidelines.xhtml

Table S1. ICD-9 codes for indications identified as acute sinusitis and viral respiratory conditions in the OPEN Stewardship platform.

OPEN Stewardship category	Indication	ICD-9 codes
Acute sinusitis	Acute sinusitis	461 Acute sinusitis
Viral respiratory conditions	Asthma/allergy	477 Allergic rhinitis, 493 Asthma, 995.3 Allergy, unspecified
	Bronchitis/bronchiolitis	466 Acute bronchitis and bronchiolitis, 490 Bronchitis, not specified as acute or chronic, 492 Emphysema, 496 Chronic airway obstruction, not elsewhere classified
	Influenza	487 Influenza, 488 Influenza due to identified avian influenza virus
	Viral pneumonia	480 Viral pneumonia
	Viral upper respiratory tract infection	460 Acute nasopharyngitis [common cold], 464 Acute laryngitis and tracheitis, 465 Acute upper respiratory infections of multiple or unspecified sites, 786.2 Cough

For peer review only

Appendix 1. Survey questions for participants in the OPEN Stewardship trial (English).

Introductory text: Thank you for agreeing to receive the OPEN Stewardship report on antibiotic prescribing. Help us improve future reports by filling out this short survey. It will only take 3-5 minutes. Questions are multiple choice with the option to provide comments at the end of the survey. Your feedback is important to us and your opinion matters!

Survey 1 (t_0)

Q1. Is the purpose of the OPEN Stewardship report clear?

- Yes
- No
- Don't know

Q2. How informative did you find the following item in the OPEN Stewardship report: Your Prescribing Rate per Visit Compared to Peers (example below - not your data).

For peer review only

Number of Prescriptions of Any Antibiotic (Per 100 Visits) for Any Indication

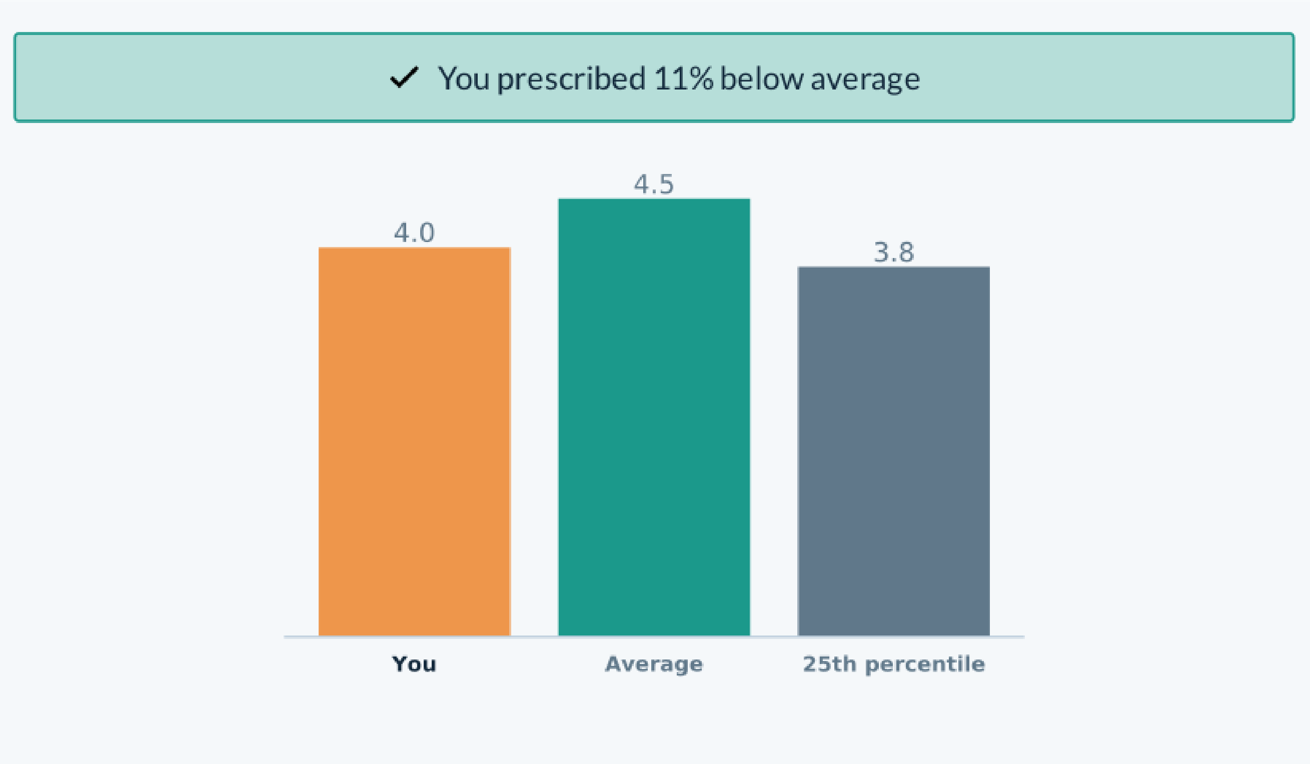

You had 5001 patient visits from Jan 01, 2019 to Dec 31, 2019 .

This figure shows how frequently you prescribed antibiotics for any indication in the previous year (2019). Your prescribing rate (prescriptions per 100 visits) is compared to the average (mean) of your colleagues and participants in Southern Ontario as well as showing your position relative to the 25th percentile. Generally, it is better to be below the average rate of prescribing and within the lowest 25th percentile.

These data are not adjusted for physician's practice characteristics, nor do they differentiate between delayed or non-filled prescriptions. These results are generated from claims data, and misclassification of the diagnosis may be present. As a result, our prescribing targets are referenced to peer benchmarks. Learn more about the data and its interpretation.

- Very informative
- Somewhat informative
- Not so informative
- Not at all informative
- Don't know

Q3. Was it straightforward how to navigate through the OPEN Stewardship report?

- Yes
- No
- Don't know

Q4. Is it useful to include *Best Practice Guidelines* in the OPEN Stewardship report (sample from most recent report below)?

Acute sinusitis (Adult)

COMMON PATHOGENS

Viruses | Streptococcus pneumoniae (non-meningitis) | Moraxella sp. | Haemophilus sp.

RECOMMENDED TREATMENT

First Line

Treatment Regimen Option

Drug	Dose	Frequency	Route	Duration
**No Antibiotic Treatment Indicated**	. mg	Once	PO	. days

Most acute sinusitis is viral in origin and does not require antibiotics. Antibiotics may be considered if symptoms are persistent and not improving after >10 days, are severe for >3 days, or improved then worsened and lasting >3 days. See below for 1st line antibiotic treatment if there is an indication.

Treatment Regimen Option

Drug	Dose	Frequency	Route	Duration
**Amoxicillin**	500 mg	TID	PO	5-7 days

- Yes
- No
- Don't know

Q5. Rate each of the following items according to its potential usefulness to you in an OPEN Stewardship report. Note that not all of these items were included in the report sent to you. (Very useful, Somewhat useful, Not so useful, Not at all useful, Don't know)

- See how my prescribing changes over time
- Compare my own prescribing with peers
- Get an overview of local prescribing patterns
- Get an overview of local resistance levels
- Compare my own prescribing with guidelines
- Have discussions with peers on the local situation regarding prescribing patterns

Q6. Overall, how would you rate the usefulness of the OPEN Stewardship report?

- Very useful
- Somewhat useful
- Not so useful
- Not at all useful
- Don't know

Q7. Please suggest how to improve the OPEN Stewardship report (such as layout of the graphs and information included).

[Free text box]

Q8. Any additional comments?

[Free text box]

Survey 2 (t_3)

Q1. How informative did you find the following item in the OPEN Stewardship report: Your Prescribing Rate per Visit Compared to Peers (example below - not your data).

For peer review only

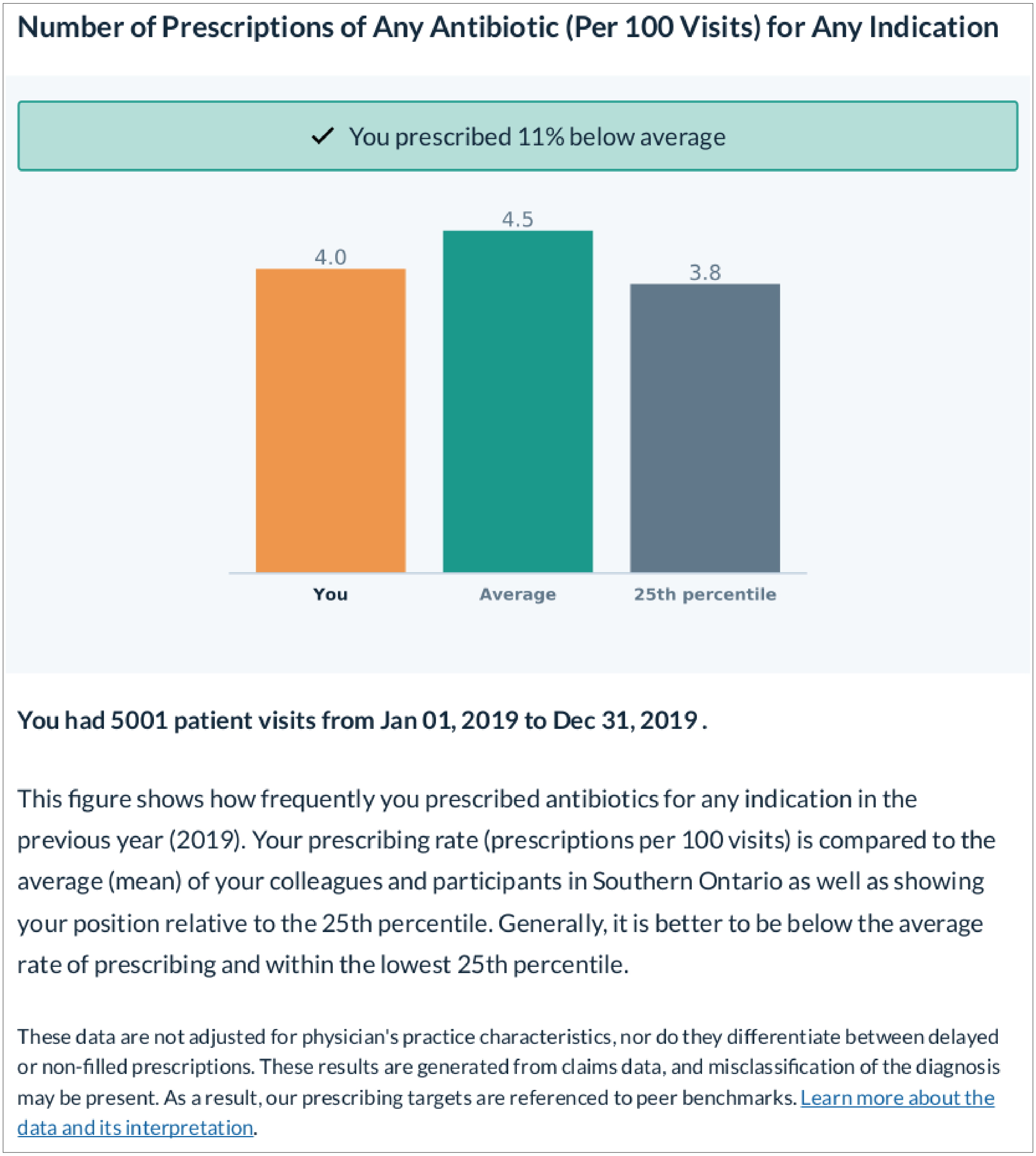

- Very informative
- Somewhat informative
- Not so informative
- Not at all informative
- Don’t know

Q2. In the past three months, did you ever consider the previous OPEN Stewardship report when making an antibiotic prescribing decision?

- Yes
- No
- Don’t know

Q3. Rate each of the following items according to its potential usefulness to you in the OPEN Stewardship report. (Very useful, Somewhat useful, Not so useful, Not at all useful, Don’t know)

- See how my prescribing changes over time
- Compare my own prescribing with peers
- Get an overview of local prescribing patterns
- Compare my own prescribing with guidelines
- Have discussions with peers on the local situation regarding prescribing patterns

Q4. Overall, how would you rate the usefulness of the OPEN Stewardship report?

- Very useful
- Somewhat useful
- Not so useful
- Not at all useful
- Don’t know

Q5. Please suggest how to improve the OPEN Stewardship report (such as layout of the graphs and information included).

[Free text box]

Q6. Any additional comments?

[Free text box]

Survey 3 (t_6)

Q1. How informative did you find the following item in the OPEN Stewardship report: Your Prescribing Rate per Visit Compared to Peers (example below - not your data).

For peer review only

Number of Prescriptions of Any Antibiotic (Per 100 Visits) for Any Indication

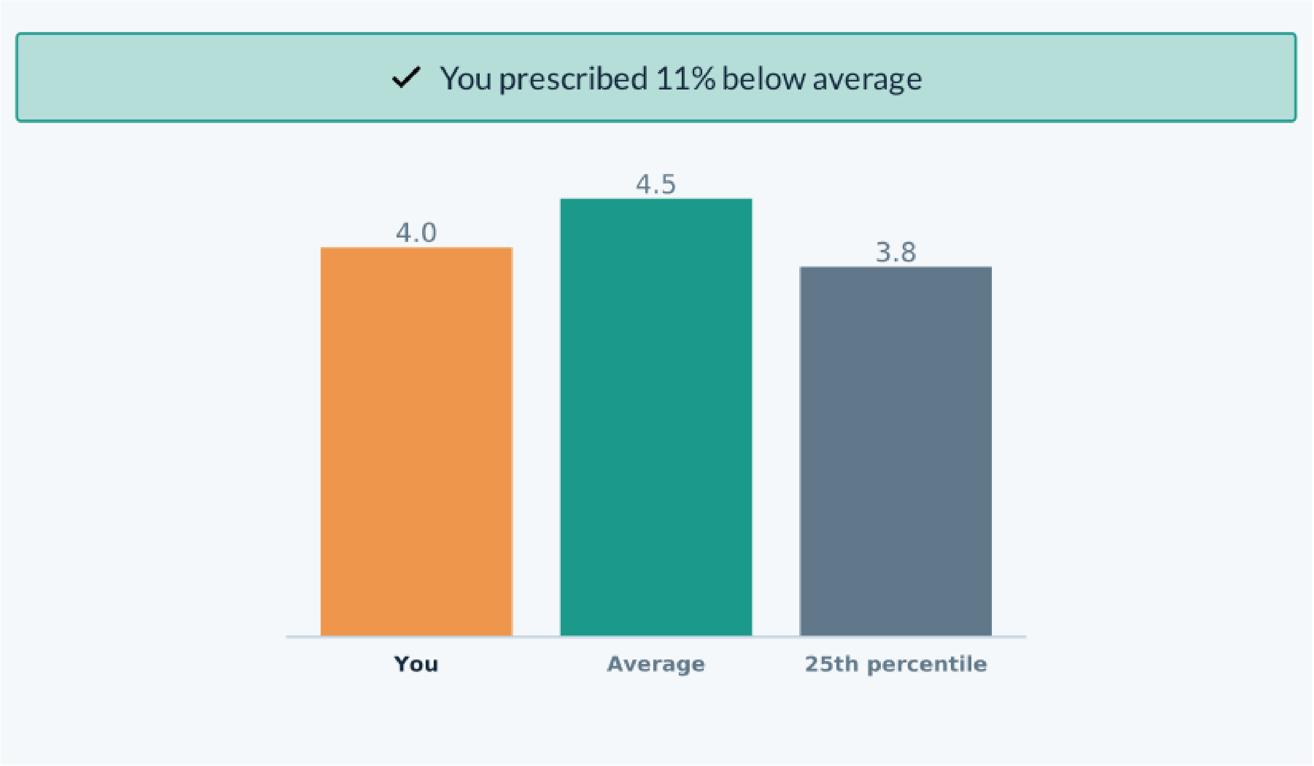

You had 5001 patient visits from Jan 01, 2019 to Dec 31, 2019 .

This figure shows how frequently you prescribed antibiotics for any indication in the previous year (2019). Your prescribing rate (prescriptions per 100 visits) is compared to the average (mean) of your colleagues and participants in Southern Ontario as well as showing your position relative to the 25th percentile. Generally, it is better to be below the average rate of prescribing and within the lowest 25th percentile.

These data are not adjusted for physician's practice characteristics, nor do they differentiate between delayed or non-filled prescriptions. These results are generated from claims data, and misclassification of the diagnosis may be present. As a result, our prescribing targets are referenced to peer benchmarks. Learn more about the data and its interpretation.

- Very informative
- Somewhat informative
- Not so informative
- Not at all informative
- Don’t know

Q2. In the past three months, did you ever consider the previous OPEN Stewardship report when making an antibiotic prescribing decision?

- Yes
- No
- Don’t know

Q3. Overall, how would you rate the usefulness of the OPEN Stewardship report?

- Very useful
- Somewhat useful
- Not so useful
- Not at all useful
- Don’t know

Q4. Hypothetically, how often do you think it would be appropriate to receive OPEN Stewardship reports?

- Monthly
- Quarterly
- Every six months
- Annually
- Not at all
- Don’t know

Q5. Would a central repository of local treatment guidelines be useful for your practice?

- Yes
- No
- Don’t know

Q6. Do you think it would be useful to have access to a personalized web-portal where you could browse your own and local patterns of antibiotic use, as well as best practice guidelines?

- Yes
- No
- Don’t know

Q7. Would you be willing to participate in a follow-up interview where we would ask in more detail about your experiences with the OPEN Stewardship reports? If so, please send an email to [study email address].

Q8. Please suggest how to improve the OPEN Stewardship report (such as layout of the graphs and information included).

[Free text box]

Q9. Any additional comments?

[Free text box]

For peer review only

Appendix 2. Survey questions for participants in the OPEN Stewardship trial (Hebrew).

Introductory text:

רופא יקר,

אנו מודים לך שוב על הסכמתך לקחת חלק במחקר המשותף של אגף רפואה בחטיבת הקהילה בשירותי בריאות כללית והמחלקה לניהול מערכות בריאות באוניברסיטת בן גוריון בנגב בנושא "דפוסי רישום תרופות אנטיביוטיות על-ידי רופאי משפחה".

נודה לך אם תוכל להקדיש שלוש עד חמש דקות מזמנך כדי לענות על השאלון שלהלן, המתייחס לדו"ח האישי שנשלח אליך לאחרונה בנושא מרשמי האנטיביוטיקה של מטופליך. תשובותיך יעזרו לנו לשפר את אופן העברת המידע לרופאים בהמשך.

השאלון נכתב בלשון זכר מטעמי נוחות, אך מיועד לשני המינים.

Survey 1 (t_0)

שאלה 1: האם מטרת הדו"ח האישי ששלחנו לך, ברורה, לדעתך?

- כן
- לא
- לא יודע

שאלה 2: באיזו מידה הפריט הבא – "שיעור מרשמי האנטיביוטיקה שרשמת פר ביקור מטופל בהשוואה לעמיתיך" - סיפק לך את המידע הדרוש לך? (הנתונים למטה לצורך דוגמא בלבד- אינם הנתונים שלך)

For peer review only

Number of Prescriptions of Any Antibiotic (Per 100 Visits) for Any Indication

✓ You prescribed 11% below average

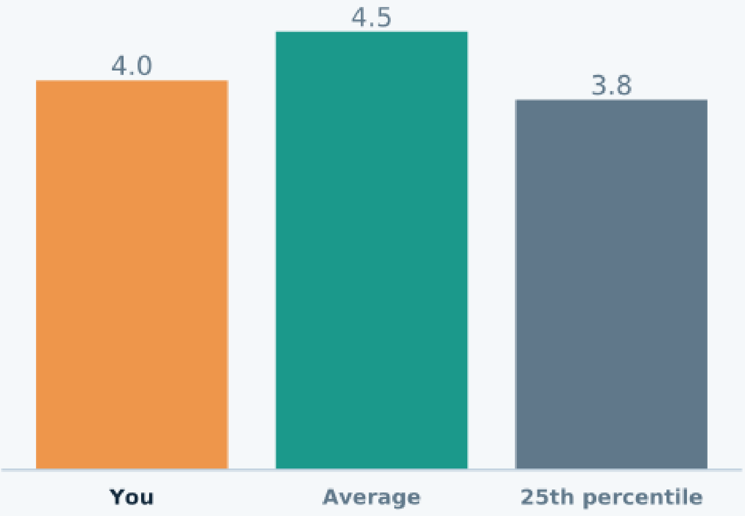

You had 5001 patient visits from Jan 01, 2019 to Dec 31, 2019 .

This figure shows how frequently you prescribed antibiotics for any indication in the previous year (2019). Your prescribing rate (prescriptions per 100 visits) is compared to the average (mean) of your colleagues and participants in Southern Ontario as well as showing your position relative to the 25th percentile. Generally, it is better to be below the average rate of prescribing and within the lowest 25th percentile.

These data are not adjusted for physician's practice characteristics, nor do they differentiate between delayed or non-filled prescriptions. These results are generated from claims data, and misclassification of the diagnosis may be present. As a result, our prescribing targets are referenced to peer benchmarks. Learn more about the data and its interpretation.

- מידה רבה
- במידה מסוימת
- במידה מועטה
- בכלל לא
- לא יודע

שאלה 3: האם הדוח האישי היה קל להבנה?

- כן

- לא
- לא יודע

שאלה 4: האם יש, לדעתך, תועלת בצירוף קווים מנחים לדו"ח האישי (ראה דוגמה להלן)?

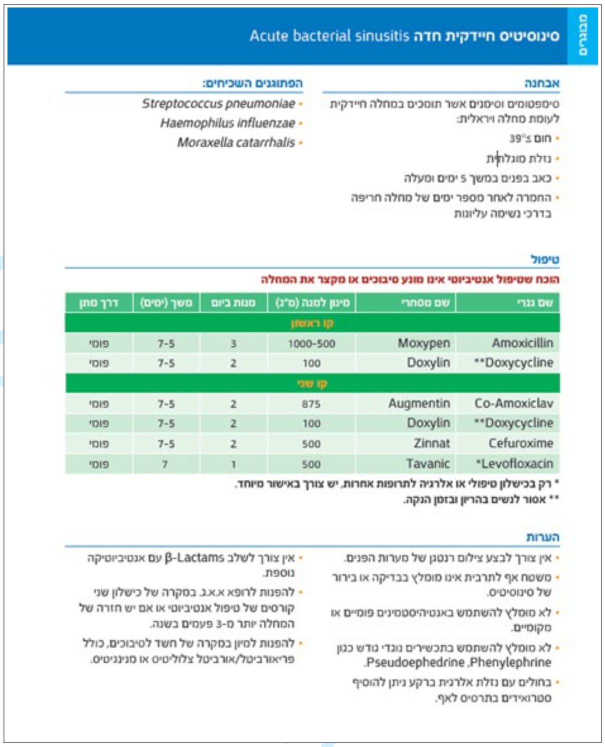
מבוגרים

סינוסיטיס חיידקית חדה Acute bacterial sinusitis

אבחנה

סימפטומים וסימנים אשר תומכים במחלה חיידקית לעומת מחלה ויראלית:

- חום ≥39°
- נזלת מוגלתית
- כאב בפנים במשך 5 ימים ומעלה
- החמרה לאחר מספר ימים של מחלה חריפה בדרכי נשימה עליונות

הפתוגנים השכיחים:

- *Streptococcus pneumoniae*
- *Haemophilus influenzae*
- *Moraxella catarrhalis*

טיפול

הוכח שטיפול אנטיביוטי אינו מונע סיבוכים או מקצר את המחלה

שם גנרי	שם מסחרי	מינון למנה (מ"ג)	מנות ביום	משך (ימים)	דרך מתן
קו ראשון					
Amoxicillin	Moxypen	1000-500	3	7-5	פומי
**Doxycycline	Doxylin	100	2	7-5	פומי
קו שני					
Co-Amoxiclav	Augmentin	875	2	7-5	פומי
**Doxycycline	Doxylin	100	2	7-5	פומי
Cefuroxime	Zinnat	500	2	7-5	פומי
*Levofloxacin	Tavanic	500	1	7	פומי

*** רק בכישלון טיפולי או אלרגיה לתרופות אחרות, יש צורך באישור מיוחד.**
**** אסור לנשים בהריון ובזמן הנקה.**

הערות

- אין צורך לבצע צילום רנטגן של מערות הפנים.
- משטח אף לתרבית אינו מומלץ בבדיקה או בירור של סינוסיטיס.
- לא מומלץ להשתמש באנטיהיסטמינים פומיים או מקומיים.
- לא מומלץ להשתמש בתכשירים נוגדי גודש כגון Phenylephrine, Pseudoephedrine.
- בחולים עם נזלת אלרגית ברקע ניתן להוסיף סטרואידים בתרסיס לאף.
- אין צורך לשלב β-Lactams עם אנטיביוטיקה נוספת.
- להפנות לרופא א.א.ג. במקרה של כישלון שני קורסים של טיפול אנטיביוטי או אם יש חזרה של המחלה יותר מ-3 פעמים בשנה.
- להפנות למיון במקרה של חשד לסיבוכים, כולל פריאורביטל/אורביטל צלוליטיס או מנינגיטיס.

- כן
- לא
- לא יודע

שאלה 5: אנא הענק ציון לכל אחד מההיגדים הבאים, בהתאם לתועלת הפוטנציאלית עבורך בדו"ח האישי ששלחנו לך שים לב, לא כל המשפטים/היגדים שאתה נשאל עליהם הופיעו בדו"ח האישי שקבלת. (מועיל מאוד, מועיל במידה מסוימת, מועיל במידה מועטה, כלל לא מועיל, לא יודע)

- אני יכול לראות כיצד דפוס רישום האנטיביוטיקה שלי משתנה לאורך זמן
- אני יכול להשוות את דפוס רישום האנטיביוטיקה שלי לאלה של עמיתיי
- אני יכול לקבל סקירה של דפוסי המרשם המקומיים
- קבלת דוח סקירה מקומי על רמות עמידות
- אני יכול להשוות את דפוס הרישום שלי לעומת הקווים המנחים של הכללית
- מאפשר לי לדון עם עמיתיי על מצב דפוסי המרשם המקומיים

שאלה 6: בסך הכל, איזה ציון היית מעניק לתועלת שאתה מפיק מהדו"ח האישי?

- מועיל מאוד
- מועיל במידה מסוימת
- מועיל במידה מועטה
- כלל לא מועיל
- לא יודע

שאלה 7: אנא הצע דרכים לשיפור הדו"ח האישי בנוגע להיבטים הבאים (עיצוב הגרף והפרמטרים).

[טקסט חופשי]

שאלה 8: הערות נוספות?

[טקסט חופשי]

Survey 2 (t_3)

שאלה 1: באיזו מידה הפריט הבא – "שיעור מרשמי האנטיביוטיקה שרשמת פר ביקור מטופל בהשוואה לעמיתיך" - סיפק לך את המידע הדרוש לך? (הנתונים למטה לצורך דוגמא בלבד- אינם הנתונים שלך)

For peer review only

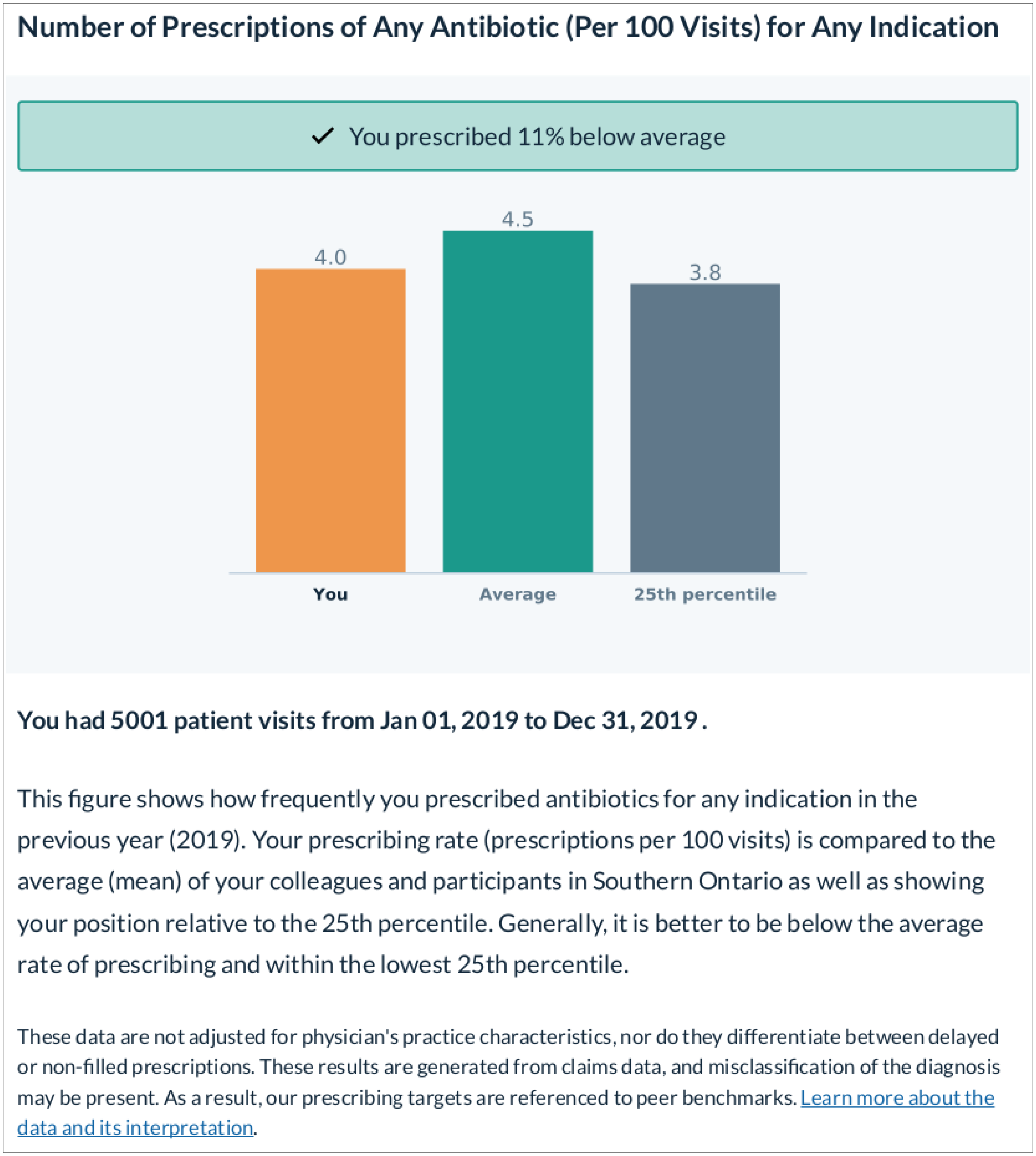

- מידה רבה
- במידה מסוימת
- במידה מועטה
- בכלל לא
- לא יודע

שאלה 2: האם במהלך שלושת החודשים האחרונים לקחת בחשבון את תוצאות הדוח הקודם לצורך קבלת החלטה על רישום אנטיביוטיקה למטופל?

- כן
- לא
- לא יודע

שאלה 3: אנא הענק ציון לכל אחד מההיגדים הבאים, בהתאם לתועלת הפוטנציאלית עבורך בדו"ח האישי ששלחנו לך. (מועיל מאוד, מועיל במידה מסוימת, מועיל במידה מועטה, כלל לא מועיל, לא יודע)

- אני יכול לראות כיצד דפוס רישום האנטיביוטיקה שלי משתנה לאורך זמן
- אני יכול להשוות את דפוס רישום האנטיביוטיקה שלי לאלה של עמיתיי
- אני יכול לקבל סקירה של דפוסי המרשם המקומיים
- אני יכול להשוות את דפוס הרישום שלי לעומת הקווים המנחים של הכללית
- מאפשר לי לדון עם עמיתיי על מצב דפוסי המרשם המקומיים

שאלה 4: בסך הכל, איזה ציון היית מעניק לתועלת שאתה מפיק מהדו"ח האישי?

- מועיל מאוד
- מועיל במידה מסוימת
- מועיל במידה מועטה
- כלל לא מועיל
- לא יודע

שאלה 5: אנא הצע דרכים לשיפור הדו"ח האישי בנוגע להיבטים הבאים (עיצוב הגרף והפרמטרים).

[טקסט חופשי]

שאלה 6: הערות נוספות?

[טקסט חופשי]

Survey 3 (t_6)

שאלה 1: באיזו מידה הפריט הבא – "שיעור מרשמי האנטיביוטיקה שרשמת פר ביקור מטופל בהשוואה לעמיתיך" - סיפק לך את המידע הדרוש לך? (הנתונים למטה לצורך דוגמא בלבד- אינם הנתונים שלך)

For peer review only

Number of Prescriptions of Any Antibiotic (Per 100 Visits) for Any Indication

✓ You prescribed 11% below average

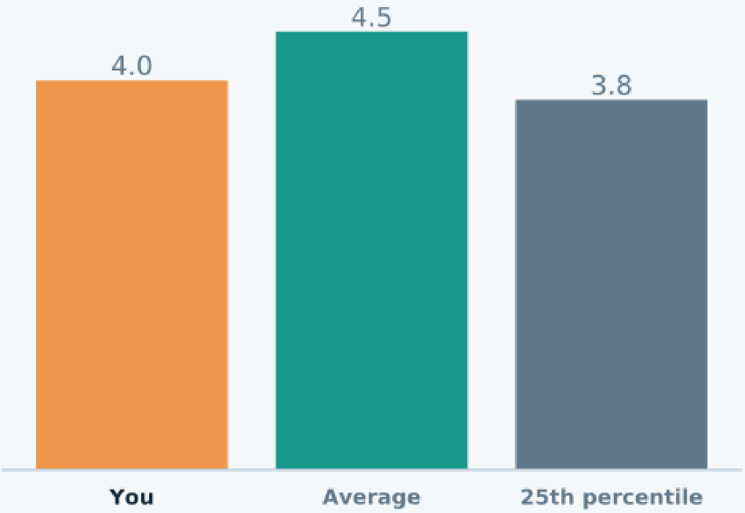

You had 5001 patient visits from Jan 01, 2019 to Dec 31, 2019 .

This figure shows how frequently you prescribed antibiotics for any indication in the previous year (2019). Your prescribing rate (prescriptions per 100 visits) is compared to the average (mean) of your colleagues and participants in Southern Ontario as well as showing your position relative to the 25th percentile. Generally, it is better to be below the average rate of prescribing and within the lowest 25th percentile.

These data are not adjusted for physician's practice characteristics, nor do they differentiate between delayed or non-filled prescriptions. These results are generated from claims data, and misclassification of the diagnosis may be present. As a result, our prescribing targets are referenced to peer benchmarks. Learn more about the data and its interpretation.

- מידה רבה
- במידה מסוימת
- במידה מועטה
- בכלל לא
- לא יודע

שאלה 2: האם במהלך שלושת החודשים האחרונים לקחת בחשבון את תוצאות הדוח הקודם לצורך קבלת החלטה על רישום אנטיביוטיקה למטופל?

- כן
- לא
- לא יודע

שאלה 3: בסך הכל, איזה ציון היית מעניק לתועלת שאתה מפיק מהדו"ח האישי?

- מועיל מאוד
- מועיל במידה מסוימת
- מועיל במידה מועטה
- כלל לא מועיל
- לא יודע

שאלה 4: באופן תיאורטי, מהי התדירות הרצויה לדעתך לקבלת הדו"ח האישי?

- אחת לחודש
- אחת לרבעו
- אחת לחצי שנה
- אחת לשנה
- בכלל לא

שאלה 5: האם מאגר מרכזי של קווים מנחים מקומיים עשוי להועיל לך בעבודתך?

- כן
- לא
- לא יודע

שאלה 6: האם אתה עשוי למצוא תועלת בקבלת גישה לפורטל אישי ברשת האינטרנט, בו תוכל למצוא את דפוסי רישום האנטיביוטיקה האישיים שלך וכן את דפוסי רישום האנטיביוטיקה המקומיים, בצירוף הקווים המנחים הרלוונטיים?

- כן
- לא
- לא יודע

שאלה 7: האם תהיה מוכן להשתתף בראיון עוקב (follow-up), בו תישאל על ניסיונך עם הדו"חות האישיים בצורה מפורטת יותר?

- כן
- לא
- לא יודע

שאלה 8: אנא הצע דרכים לשיפור הדו"ח האישי בנוגע להיבטים הבאים (עיצוב הגרף והפרמטרים).

[טקסט חופשי]

שאלה 9: הערות נוספות?

[טקסט חופשי]

For peer review only

Appendix 3. OPEN Stewardship feedback survey pilot protocol and interview guide.

OPEN Stewardship Survey: Pilot testing of questionnaires #1, #2 and #3
***** INTERVIEW GUIDE AND TEMPLATE FOR RESPONSES *****

Purpose of the pilots

- To identify issues (clarity, technical errors etc) in the questionnaires and find potential solutions to improve the questionnaires, key points being:
  - Do the respondents understand the purpose of the questionnaire?
  - Do the respondents find the questions relevant?
  - Do the respondents think that there are questions that are missing?
- To get a realistic estimate of the response time.

Before the interview

- **Identify respondents and book time**
- Identify two respondents from each country and sector (humans/companion animals/dairy) who are willing to pilot test the questionnaire, either among those that have been recruited to the OPEN Stewardship study or other similar persons (or a combination of both), do what is most feasible.
- If possible, identify pilot respondents who are geographically close to you so that you can sit next to the respondent when conducting the pilot interview. An interview over phone, Skype or similar is acceptable but a less preferred option.
- If possible, of the two persons per country and sector, select one person with more years with relevant practice and one with less.
- Each pilot respondent will go through two questionnaires: one which is identical to questionnaire #1 and one which includes only those questions that are added to questionnaire #2 and to questionnaire #3 (referred to as pilot questionnaire #2)
  Estimated time for the pilot session is 60 minutes plus introduction and wrap up time, in total up to 90 minutes, so book 1.5 hour for the interview.

- **Communicate with each respondent before the interview**
- Send a (generic) OPEN Stewardship report one week before the booked interview.
- Ask the respondent to spend about 10 minutes to read it at some point before the booked interview.
- Ask the respondent to make a note of the date they read the report.
- Inform the respondent that during the interview you will go through an online questionnaire on his or her computer/device. You will also email some information during the interview. The respondent must thus have internet access plus access to email.

- **Material the interviewer should bring to the interview**
- Laptop or similar, with:
  - This guide in electronic format
  - Draft emails with link to pilot questionnaires to send during the interview
- A printed version of the introductory text that will be sent with each questionnaire
- Pen and paper
- Watch.

During the interview

For peer review only

Use this template during the interview as a guideline for the information that should be given to the respondent. Record the responses to the interview questions in the boxes as specified.
Please fill out and save one template per respondent.
Name each file: COUNTRY_SECTOR_ABBREVIATED-NAME-OF-RESPONDENT (first two letters of given name and first two letters of family name).
You may of course use your own words whenever you wish.

Name of interviewer
Fill out here

Abbreviated name of respondent (first two letters of given name and family name respectively)
Fill out here

- **INTRODUCTION TO INTERVIEW**

Thank you for taking the time to do this interview. As I mentioned previously, I estimate that it will take about 60 minutes.
The aim of the OPEN Stewardship project is to improve appropriate antibiotic prescribing by generating customised reports of individual prescribing in relation to peers. We sent you one of these reports a week ago.
Surveys will be sent out to practitioners enrolled in the OPEN Stewardship intervention at three occasions, to improve the reports.
We are now doing interviews to find out what respondents think about the survey questions. This is to ensure that the surveys measure the right things, so that we will get as good results as possible in order to improve our system. The surveys will be sent out by email and will be filled out online.
Just so you know, I am not the one who have made the survey questions we are about to go through. All of your comments are greatly appreciated.
Approximately 10 people will be interviewed about the survey questions in this pilot. This is an international collaboration, and we work together with colleagues in Canada, Israel, Sweden and the USA [REMOVE YOUR OWN COUNTRY].
If you need a break at any time during the interview just let me know.
Any questions before we get started?

For peer review only

- **SAMPLE REPORT INFORMATION**

Have you had time to read the OPEN Stewardship report?
Interviewer fill out here

- *If not, allow for up to 10 minutes to look at it (but don't rush the person).*

Roughly, when did you read the report? *Or note if the respondent is reading it right now*
Interviewer fill out here

- **EMAIL WITH QUESTIONNAIRE INFORMATION**

So we will start by looking at the text that will accompany the questionnaire, which will be sent out through a link in an email. If you can please read through this email.

- Hand over the paper with the email text. *If the interview is done over the phone, email the text to the respondent now.*

Do you have any comments?
Interviewer fill out here
Is there anything you feel is unclear?
Interviewer fill out here

Do you think the text would motivate someone to answer the survey?
Interviewer fill out here
Any suggestions?
Interviewer fill out here

Thank you!

- **QUESTIONNAIRE 1**

We will now move on to the first questionnaire, and I will email you a link to this questionnaire.

- Email the link to pilot questionnaire 1.

I would like to ask you to fill out the whole questionnaire, but don't press "submit" at the end. Take your time. If you wish you can make notes if there is anything that you come to think of; we will later go through the questionnaire question by question.

For peer review only

- Give the respondent a piece of paper and a pen.
- Respondent fills out the questionnaire.
- Note how long it takes in minutes to complete questionnaire.

Interviewer fill out here

- Note if the respondent seems to spend a longer time reading/re-reading any of the questions.

Interviewer fill out here

Thanks!
So let us start from the beginning with the introduction to the survey.

Do you think this text is informative enough? Any suggestions?
Interviewer fill out here

We will now go through each question, one at a time. I am interested in your opinion, in this situation there is nothing that is right or wrong. So we start with the first question.

- In your discussion with the respondent, note also if anything he or she says that is unexpected/odd.

- **"Is the purpose of the OPEN Stewardship report clear?"**

Can you please describe why you gave this response?
Interviewer fill out here

- **"How informative did you find the following item in the OPEN Stewardship report: Your Prescribing Rate per Visit Compared to Peers"**

Can you please describe why you gave this response?
Interviewer fill out here

Is it clear to you what the various responses mean?
Interviewer fill out here

- **"Was it straightforward how to navigate through the OPEN Stewardship report?"**

Can you please describe why you gave this response?
Interviewer fill out here

For peer review only

- **"Is it useful to include Best Practice Guidelines in the OPEN Stewardship report"**

Can you please describe why you gave this response?
Interviewer fill out here

- **"Rate each of the following items according to its potential usefulness to you in the OPEN Stewardship report."**

- **"See how my prescribing changes over time"**

Can you please describe why you gave this response?
Interviewer fill out here

- **"Compare my own prescribing with peers"**

Can you please describe why you gave this response?
Interviewer fill out here

- **"Get an overview of local prescribing patterns"**

Can you please describe why you gave this response?
Interviewer fill out here

- **"Compare my own prescribing with guidelines"**

Can you please describe why you gave this response?
Interviewer fill out here

- **"Have discussions with my peers on the local situation … "**

Can you please describe why you gave this response?
Interviewer fill out here

Is it clear to you what the various responses mean?
Interviewer fill out here

- **"Overall, how would you rate the usefulness of the OPEN Stewardship report?"**

For peer review only

Can you please describe why you gave this response?
Interviewer fill out here

- **“Please suggest how to improve the OPEN Stewardship report:”**
- **“Layout of the graphs (such as colors, size, legends).”**

Is it clear to you what kind of responses could be given here?
Interviewer fill out here

- **“Information included (such as number of graphs, explanatory text).”**

Is it clear to you what kind of responses could be given here?
Interviewer fill out here

- **“Other comments.”**

Is it clear to you what kind of responses could be given here?
Interviewer fill out here

I would now like to continue with the layout of the questionnaire.

- Note any potential changes that are indicated by the responses.

What was the most difficult part to navigate through?
Interviewer fill out here

For which questions did you have to use the scroll bar?
Interviewer fill out here

For peer review only

If you now think about the questionnaire as a whole,

What is your overall impression?
Interviewer fill out here

After reading these questions – what would you say is the purpose of this survey?
Interviewer fill out here

Are there any questions that are *not* relevant?
Interviewer fill out here

Are there any questions that should be phrased differently?
Interviewer fill out here

Is any type of question missing?
Interviewer fill out here

What about the length of the survey?
Interviewer fill out here

- **QUESTIONNAIRE 2**

The second and last questionnaire that I want you to go through contains some other questions that we want to ask when evaluating the project and the reports on prescription patterns. It contains 7 questions. I will now email you the link to this questionnaire.

- Email the link to pilot questionnaire 2.

I would like to ask you to now fill out the second questionnaire.

- Respondent fills out the questionnaire.

Again we will now go through each question one at a time.

- **"1. In the past three months, did you ever think about your previous OPEN Stewardship report when making an antibiotic prescribing decision?"**

Can you please describe why you gave this response?
Interviewer fill out here

- **"2. In general, how useful do you think the OPEN Stewardship report would be to other prescribers?"**

Can you please describe why you gave this response?
Interviewer fill out here

Is it clear to you what the various responses mean?
Interviewer fill out here

- **"3. Hypothetically, how often do you think it would be appropriate to receive OPEN Stewardship reports?"**

Can you please describe why you gave this response?
Interviewer fill out here

Is it clear to you what the various responses mean?
Interviewer fill out here

- **"4. Does the pdf format of the OPEN Stewardship report work well for you?"**

Can you please describe why you gave this response?
Interviewer fill out here

- **"5. Would a central repository of local treatment guidelines be useful for your practice?"**

What does this question mean, can you describe it in other words?
Interviewer fill out here

- **"6. Do you think it would be useful to have access to a personalized web-portal where you could browse your own and local patterns of antibiotic use, as well as best practice guidelines?"**

What does this question mean, can you describe it in other words?
Interviewer fill out here

For peer review only

- **"7. Would you be willing to participate in a follow-up interview where we would ask in more detail about your experiences with the OPEN Stewardship reports?"**

Can you please describe why you gave this response?
Interviewer fill out here

- **END OF INTERVIEW**

- Note which kind of device and browser the respondent uses.

Interviewer fill out here

We have now finished. On behalf of me and all my colleagues in the project, I would like to thank you so much for taking the time to help us to improve the evaluation. It has been most useful for our work. If there is anything that you come to think of afterwards, do not hesitate to contact me.

After the interview

Email the filled out templates (in English in case you have used another language for your notes) to the Public Health Agency of Sweden who will compile from all sites. Make sure that the file names are: COUNTRY_SECTOR_ABBREVIATED-NAME-OF-RESPONDENT.

For peer review only
